# Piano-Stool Ruthenium(II) Complexes with Delayed Cytotoxic Activity: Origin of the Lag Time

**DOI:** 10.1021/acs.inorgchem.1c00507

**Published:** 2021-05-12

**Authors:** Laia Rafols, Dana Josa, David Aguilà, Leoní A. Barrios, Olivier Roubeau, Jordi Cirera, Vanessa Soto-Cerrato, Ricardo Pérez-Tomás, Manuel Martínez, Arnald Grabulosa, Patrick Gamez

**Affiliations:** † Departament de Química Inorgànica i Orgànica, Facultat de Química, Secció de Química Inorgànica, Universitat de Barcelona, Martí i Franquès, 1-11, 08028 Barcelona, Spain; ‡ Institute of Nanoscience and Nanotechnology (IN2UB), Universitat de Barcelona, 08028 Barcelona, Spain; § Instituto de Ciencia de Materiales de Aragón (ICMA), CSIC and Universidad de Zaragoza, Pedro Cerbuna 12, 50009 Zaragoza, Spain; ∥ Institut de Recerca de Química Teórica i Computacional, Universitat de Barcelona, 08028 Barcelona, Spain; ⊥ Department of Pathology and Experimental Therapeutics, Faculty of Medicine and Health Sciences, University of Barcelona, Campus Bellvitge, Feixa Llarga s/n, 08907 L’Hospitalet de Llobregat (Barcelona), Spain; # Oncobell Program, Institut d’Investigació Biomèdica de Bellvitge (IDIBELL), 08907 L’Hospitalet de Llobregat, Barcelona, Spain; ∇ Catalan Institution for Research and Advanced Studies, Passeig Lluís Companys 23, 08010 Barcelona, Spain

## Abstract

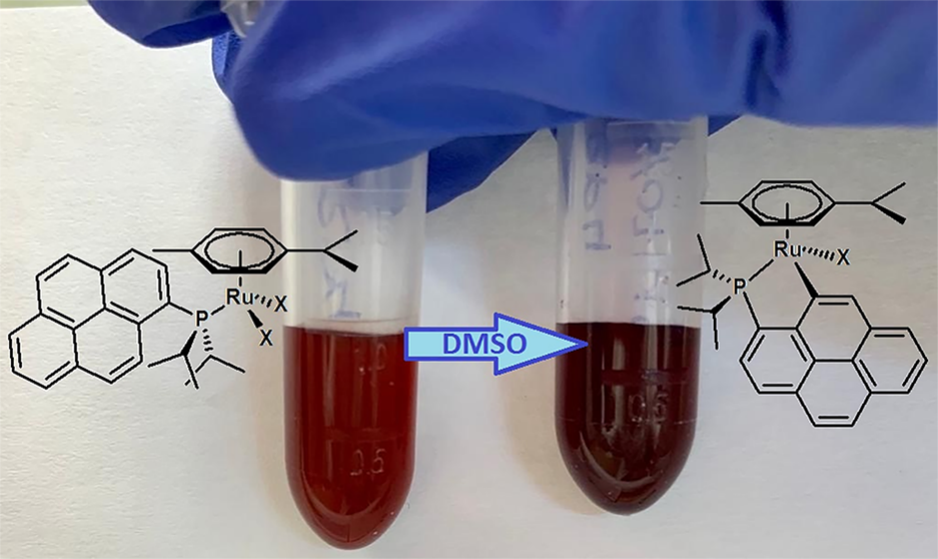

We have recently reported a series of piano-stool ruthenium(II) complexes of the general formula [RuCl_2_(η^6^-arene)(P(1-pyrenyl)R^2^R^3^)] showing excellent cytotoxic activities (particularly when R^2^ = R^3^ = methyl). In the present study, new members of this family of compounds have been prepared with the objective to investigate the effect of the steric hindrance of a bulky phosphane ligand, namely diisopropyl(1-pyrenyl)phosphane (**L**), on exchange reactions involving the coordinated halides (X = Cl, I). Two η^6^-arene rings were used, i.e. η^6^-methyl benzoate (mba) and η^6^-*p*-cymene (*p*-cym), and four complexes were synthesized, namely [RuCl_2_(mba)(**L**)] (**1**_Cl_2__^*iPr*^), [RuI_2_(mba)(**L**)] (**1**_I_2__^*iPr*^), [RuCl_2_(*p*-cym)(**L**)] (**2**_Cl_2__^*iPr*^), and [RuI_2_(*p*-cym)(**L**)] (**2**_I_2__^*iPr*^). Unexpectedly, all of the complexes exhibited poor cytotoxic activities after 24 h of incubation with cells, in contrast to the related compounds previously reported. However, it was observed that aged DMSO solutions of **2**_I_2__^*iPr*^ (from 2 to 7 days) exhibited better activities in comparison to freshly prepared solutions and that the activity improved over “aging” time. Thorough studies were therefore performed to uncover the origin of this lag time in the cytotoxicity efficiency. The data achieved clearly demonstrated that compounds **2**_I_2__^*iPr*^ and **2**_Cl_2__^*iPr*^ were undergoing a series of transformation reactions in DMSO (with higher rates for the iodido complex **2**_I_2__^*iPr*^), ultimately generating cyclometalated species through a mechanism involving DMSO as a coordinated proton abstractor. The cyclometalated complexes detected in solution were subsequently prepared; hence, pure [RuCl(*p*-cym)(κ^2^*C*-diisopropyl(1-pyrenyl)phosphane)] (**3**_Cl_^*iPr*^), [RuI(*p*-cym)(κ^2^*C*-diisopropyl(1-pyrenyl)phosphane)] (**3**_I_^*iPr*^), and [Ru(*p*-cym)(κ*S*-dmso)(κ^2^*C*-diisopropyl(1-pyrenyl)phosphane)]PF_6_ (**3**_dmso_^*iPr*^) were synthesized and fully characterized. Remarkably, **3**_Cl_^*iPr*^, **3**_I_^*iPr*^, and **3**_dmso_^*iPr*^ are all very efficient cytotoxic agents, exhibiting slightly better activities in comparison to the chlorido noncyclometalated complexes [RuCl_2_(η^6^-arene)(P(1-pyrenyl)R^2^R^3^)] described in an earlier report. For comparison purposes, the iodido compounds [RuI_2_(mba)(dimethyl(1-pyrenyl)phosphane)] (**1**_I_2__^*Me*^) and [RuI_2_(*p*-cym)(dimethyl(1-pyrenyl)phosphane)] (**2**_I_2__^*Me*^), bearing the less hindered dimethyl(1-pyrenyl)phosphane ligand, have also been prepared. The cytotoxic and chemical behaviors of **1**_I_2__^*Me*^ and **1**_I_2__^*Me*^ were comparable to those of their chlorido counterparts reported previously.

## Abstract 2

DMSO gradually converts half-sandwich, 1-pyrenyl-containing ruthenium(II) complexes into cyclometalated species showing notable cytotoxic properties.

## Introduction

Cancer has a major impact on society worldwide, as it represents one of the leading causes of death.^1,2^ Cisplatin is one of the most used drugs to treat various types of cancer.^3^ The remarkable chemotherapeutic properties of cisplatin have instigated tremendous research efforts in the area of platinum drugs.^4−6^ Nevertheless, cisplatin suffers from some severe side effects,^7^ and a decrease in its effectiveness may be observed with platinum-resistant tumors.^8^ Therefore, the development of more efficient and less toxic therapeutic agents is essential in this area of investigation. In that context, alternative transition metals have been used to generate new compounds.^9−14^ For instance, some ruthenium complexes have been reported that exhibit remarkable anticancer properties,^15^ making them potential drug candidates.^16−22^ Actually, two ruthenium compounds are currently undergoing clinical trials, namely BOLD-100 (Na[*trans*-RuCl_4_(Ind)_2_], Ind = indazole)^23−25^ and TLD1433 ([Ru(bpy)(IP-TT)]_2_^+^ (IP-TT = 2-(2′,2″:5′′,2‴-terthiophene)imidazo[4,5-*f*][1,10]phenanthroline).^26,27^ To date, there are no efficient molecules capable of targeting most types of disseminated tumor cells. NAMI-A shows interesting antimetastatic properties,^28^ as do some Ru(II)-arene complexes from the RAPTA family;^29,30^ for instance, RAPTA-T exerts antimetastatic activities.^31,32^ Hence, Ru-based compounds are increasingly being seen as potential next-generation anticancer metallodrugs.^33,34^

**Scheme 1:**
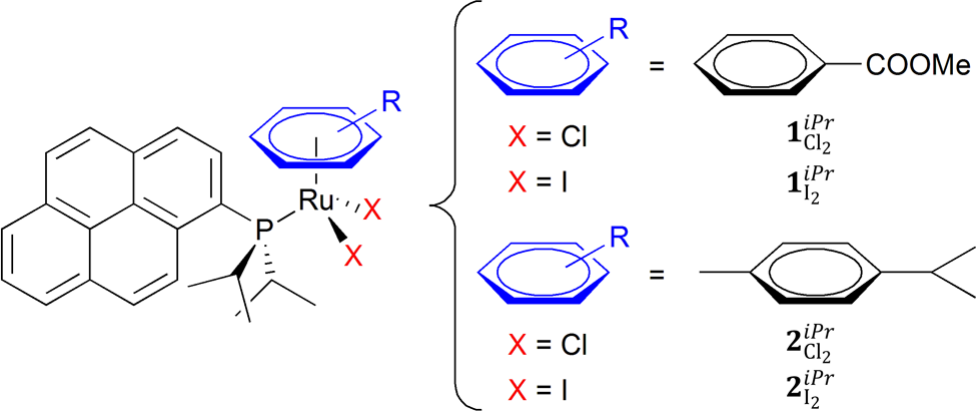
Representation of the Structure of the Piano-Stool Ruthenium Complexes Designed and Synthesized in the Present Study to Evaluate the Effect of Chloride and Iodide on the Cytotoxic Activity

Recently, we have reported a series of half-sandwich ruthenium(II) complexes of the general formula [RuX_2_(η^6^-arene)(P(1-pyrenyl)R^2^R^3^)] (with η^6^-arene = *p*-cymene, methyl benzoate, R^2^ = methyl, phenyl, and R^3^ = methyl, phenyl) displaying valuable cytotoxic behaviors.^35^ In that previous study, the effect of the nature of the η^6^-arene on the cytotoxic activity was examined (viz. *p*-cymene vs. methyl benzoate), as well as that of different R groups on the monophosphane P(1-pyrenyl)R^2^R^3^ ligand; a significant effect of these two parameters on cell toxicity was observed.^35^ Therefore, we decided to investigate the role played by the halide, i.e. X, on the biological activity of [RuX_2_(η^6^-arene)(P(1-pyrenyl)R^2^R^3^)] compounds and thus study and compare the cytotoxic properties of the four complexes, depicted in Scheme 1: viz., **1**_Cl_2__^*iPr*^, **1**_I_2__^*iPr*^, **2**_Cl_2__^*iPr*^, and **2**_I_2__^*iPr*^.

These Ru compounds all contain the ligand diisopropyl(1-pyrenyl)phosphane (**L**), purposely chosen for its steric hindrance that would favor the displacement of the halide ligands, for instance by water molecules.^36,37^ Two different arene rings were used, namely methyl benzoate and *p*-cymene, which have produced piano-stool ruthenium complexes with drastically distinct cytotoxic properties in a previous study.^35^

Surprisingly, cell viability assays with various cancer lines revealed that **1**_Cl_2__^*iPr*^, **1**_I_2__^*iPr*^, **2**_Cl_2__^*iPr*^, and **2**_I_2__^*iPr*^ were poorly active (mostly inactive) compounds after an incubation time of 24 h. However, complex **2**_I_2__^*iPr*^, namely [RuI_2_(η^6^-*p*-cymene)(diisopropyl(1-pyrenyl)phosphane)] (Scheme 1), exhibited a drastic improvement in its activity with time; for instance, **2**_I_2__^*iPr*^ was 4 times more active against lung adenocarcinoma cells after 7 days (in comparison to its activity after 1 day). This highly interesting behavior was thoroughly investigated to elucidate the origin of this time-dependent enhancement of the cytotoxic properties; the mechanistic studies carried out showed that **2**_I_2__^*iPr*^ (as well as its corresponding chlorido complex **2**_Cl_2__^*iPr*^) was gradually converted into a highly cytotoxic, cyclometalated species, through a three-step process (whose rate was halide-dependent: i.e., the conversion process was faster with iodido complex **2**_I_2__^*iPr*^ than with chlorido complex **2**_Cl_2__^*iPr*^).

## Results and Discussion

### Preparation of the Ru Compounds **1**_Cl_2__^*iPr*^, **1**_I_2__^*iPr*^, **2**_Cl_2__^*iPr*^, and **2**_I_2__^*iPr*^

The ligand, namely diisopropyl(1-pyrenyl)phosphane (**L**), was prepared by the reaction of lithiated 1-bromopyrene with chlorodiisopropylphosphane in THF at −78 °C (Scheme S1). Ligand **L** is unstable in air (an oxide of the phosphane is produced); therefore, **L** was protected by the formation of its borane adduct. **L**·BH_3_ can be deprotected, just before use (to prepare the Ru compounds), by reaction with the tetrafluoroboric acid diethyl ether adduct in dichloromethane (Scheme S1).

**Figure 1:**
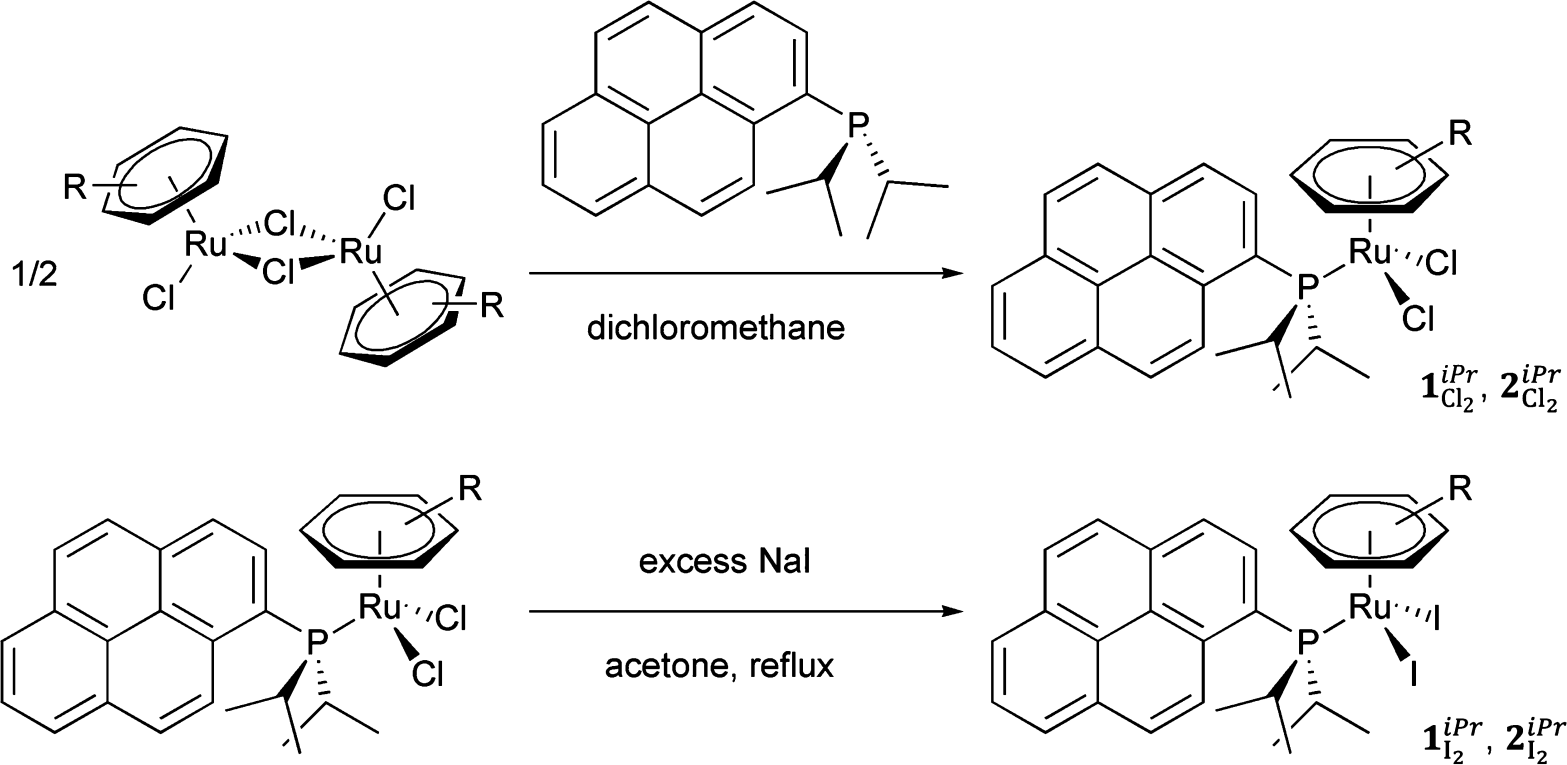
Synthetic procedures used to prepare the half-sandwich Ru(II) chlorido **1**_Cl_2__^*iPr*^ and **2**_Cl_2__^*iPr*^ and iodido complexes **1**_I_2__^*iPr*^ and **2**_I_2__^*iPr*^.

The chlorido complexes [RuCl_2_(η^6^-methyl benzoate)(**L**)] (**1**_Cl_2__^*iPr*^) and [RuCl_2_(η^6^-*p*-cymene)(**L**)] (**2**_Cl_2__^*iPr*^) were obtained in good yields by the reaction of ligand **L** with the corresponding ruthenium dimeric precursors: namely, [RuCl(μ-Cl)(η^6^-methyl benzoate)]_2_ for **1**_Cl_2__^*iPr*^ and [RuCl(μ-Cl)(η^6^-*p*-cymene)]_2_ for **2**_Cl_2__^*iPr*^ (Figure 1). The iodido compounds [RuI_2_(η^6^-methyl benzoate)(**L**)] (**1**_I_2__^*iPr*^) and [RuI_2_(η^6^-*p*-cymene)(**L**)] (**2**_I_2__^*iPr*^) can be generated in good yields from **1**_Cl_2__^*iPr*^ and **2**_Cl_2__^*iPr*^ in the presence of an excess of sodium iodide in refluxing technical acetone (Figure 1).^38^ It should be pointed out that the conversion of **2**_Cl_2__^*iPr*^ to **2**_I_2__^*iPr*^ was achieved in 1 h, whereas 16 h was required for the chloride to iodide exchange that generated **1**_I_2__^*iPr*^ from **1**_Cl_2__^*iPr*^ (see the Experimental Section). Thus, it appears that the bulkier and more electron donating *p*-cymene ring significantly favors the chloride to iodide substitution. This substitution can easily be monitored by ^31^P NMR spectroscopy. For instance, the ^31^P{^1^H} NMR spectrum of **1**_Cl_2__^*iPr*^ shows a singlet at +38.9 ppm, whereas the corresponding singlet is observed at +34.5 ppm for **1**_I_2__^*iPr*^ (Δδ −4.4 ppm). Similarly, the ^31^P chemical shift is observed at +36.3 ppm for chlorido **2**_Cl_2__^*iPr*^ and at higher field for the iodido complex **2**_I_2__^*iPr*^: viz., +31.3 ppm (Δδ −5.0 ppm). The lower electronegativity of iodine, in comparison to that of chlorine, may explain why the P atoms of the iodido complexes are more shielded.

All of the ruthenium compounds were characterized by common techniques, including X-ray diffraction, which confirmed their identity (see the Experimental Section for details).

### Crystal Structures of Ru Compounds **1**_Cl_2__^*iPr*^, **1**_I_2__^*iPr*^, **2**_Cl_2__^*iPr*^, and **2**_I_2__^*iPr*^

**Figure 2:**
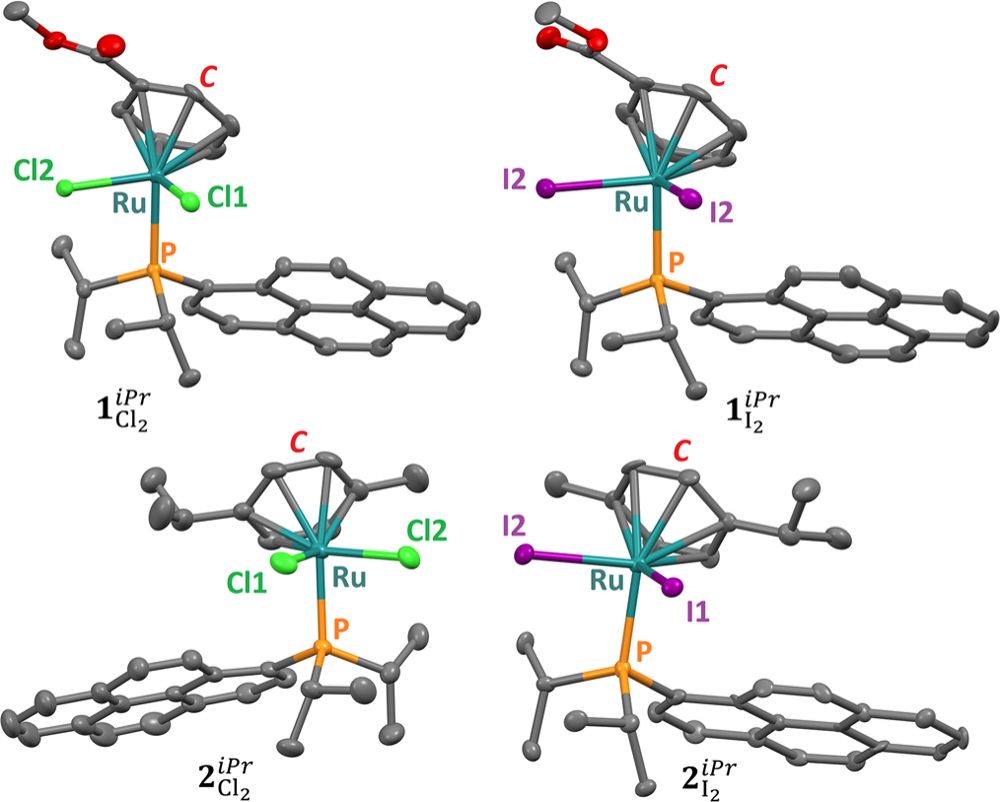
Representation of the crystal structures of complexes **1**_Cl_2__^*iPr*^, **1**_I_2__^*iPr*^, **2**_Cl_2__^*iPr*^, and **2**_I_2__^*iPr*^. The atoms bonded to the metal center are labeled, and ***C*** represents the centroid of the η^6^-arene ring. Hydrogen atoms are omitted for clarity.

Single crystals of the four compounds, suitable for X-ray diffraction studies, were obtained (see Experimental Section). Compounds **1**_Cl_2__^*iPr*^ and **1**_I_2__^*iPr*^ crystallize in the monoclinic space group *P*2_1_/*c*, compound **2**_Cl_2__^*iPr*^ crystallizes in the monoclinic space group *C*2/*c*, and **2**_I_2__^*iPr*^ crystallizes in the triclinic space group *P*1̅ (see Tables S1 and S2). The solid-state structures of the four complexes are shown in Figure 2; selected (coordination) bonds and angles are given in Table S3 (see also Figure S1).

The four ruthenium compounds exhibit the expected, typical “three-legged piano-stool” geometry for such systems. The centroid to metal distance varies from 1.69 to 1.72 Å, and the Ru–P length is in the range 2.39–2.41 Å (Table S3). The Ru–Cl bond distances are 2.41 and 2.42 Å for **1**_Cl_2__^*iPr*^ and 2.40 and 2.43 Å for **2**_Cl_2__^*iPr*^. As could be predicted, the Ru–I bonds are longer (of about 0.3 Å), with values at around 2.72 Å for **1**_I_2__^*iPr*^, and 2.72 and 2.73 Å for **2**_I_2__^*iPr*^ (Table S3). The coordination angles are similar for all four complexes (Table S3), and they are in the range expected for such molecules.^35,39,40^

### Effects of Compounds **1**_Cl_2__^*iPr*^, **1**_I_2__^*iPr*^, **2**_Cl_2__^*iPr*^, and **2**_I_2__^*iPr*^ on Cell Viability

The ability of the compounds to inhibit cell growth was evaluated next. Hence, their cytotoxic properties were first assessed in A549 cells (lung adenocarcinoma) at a fixed complex concentration of 10 μM. The corresponding cell viabilities (in percent) are given in Table 1.

**Table 1:** Cell Viability Values (%) of Complexes **1**_Cl_2__^*iPr*^, **1**_I_2__^*iPr*^, **2**_Cl_2__^*iPr*^ and **2**_I_2__^*iPr*^ (Fixed Concentration of 10 μM) for A549 (Lung Adenocarcinoma) Human Cells, after Incubation for 24 h

	**1**_Cl_2__^*iPr*^	**1**_I_2__^*iPr*^	**2**_Cl_2__^*iPr*^	**2**_I_2__^*iPr*^
cell viability (%)^a^^,^^b^	108 ± 2	104 ± 12	73 ± 18	37 ± 17

a After 24 h incubation at 37.5 °C.

b The results are expressed as mean values ± SD out of three independent experiments.

Surprisingly, the cytotoxic activities of the Ru compounds are mostly poor; for instance, **1**_Cl_2__^*iPr*^ and **1**_I_2__^*iPr*^ are inactive (cell viability of around 100%; Table 1), and **2**_Cl_2__^*iPr*^ only gives rise to some cell growth inhibition (73% cell viability; Table 1). Only compound **2**_I_2__^*iPr*^ is efficiently capable of eradicating A549 cells (37% cell viability; Table 1). An unusual phenomenon was observed during the replication experiments with **2**_I_2__^*iPr*^. Indeed, clearly different cell viability values after 24 h of incubation were obtained for the same stock solution of **2**_I_2__^*iPr*^ in DMSO, used after 1, 5 and 7 days after preparation. A significant improvement in the cytotoxic properties of **2**_I_2__^*iPr*^ was indeed noticed from day 0 of the preparation of the stock solution to, for instance, days 5 and 7; actually, the value of 37 ± 17% (Table 1) corresponds to the average value from three replicates carried out with a stock solution of **2**_I_2__^*iPr*^ used after 0, 5, and 7 days. The large deviations observed among the replicates are due to the significantly different activities of the aging solution. It was also noted that the aging DMSO solution of **2**_I_2__^*iPr*^ became slightly darker after a few days (see the graphical abstract). It thus appears that **2**_I_2__^*iPr*^ is gradually converted into a new and more active, “unknown” species.

Subsequently, half-maximum inhibitory concentrations (IC_50_) were determined for compounds **2**_Cl_2__^*iPr*^ and **2**_I_2__^*iPr*^, considering this feature (viz. the observed time-dependent evolution of the biological properties of **2**_I_2__^*iPr*^). IC_50_ values were not determined for **1**_Cl_2__^*iPr*^ and **1**_I_2__^*iPr*^, which did not show any cytotoxic behavior (see Table 1). The IC_50_ data obtained are given in Table 2.

**Table 2:** Half-Maximum Inhibitory Concentrations (IC_50_, μM) of Compounds **2**_Cl_2__^*iPr*^ and **2**_I_2__^*iPr*^ for A549 (Lung Adenocarcinoma) Human Cells, after Incubation of 24 h, Using Freshly Prepared Stock Solutions of the Complexes (Day 0), and 5- and 7-Day Aged Solutions of the Complexes^a^

cell line	compound	day 0	day 5	day 7
A549	**2**_Cl_2__^*iPr*^	24 ± 1	26 ± 10	27 ± 12
A549	**2**_I_2__^*iPr*^	48 ± 8	26 ± 8	9.5 ± 1.5
MCF7	**2**_I_2__^*iPr*^			12 ± 2

a The IC_50_ value (μM) of a 7-day-old DMSO solution of **2**_I_2__^*iPr*^ for MCF7 (breast carcinoma) human cells, after incubation for 24 h, is also included. The results are expressed as mean values ± SD out of three independent experiments.

Compound **2**_Cl_2__^*iPr*^ gives an IC_50_ value of 24 μM after 24 h incubation with A549 (lung adenocarcinoma) human cells (Table 2), using a stock solution of Ru compound (in DMSO) prepared the same day (viz. the day in which the compound was incubated with the cells, day 0). As already noticed with the cell viability studies, the use of a 5-day-old or 7-day-old stock solution of **2**_Cl_2__^*iPr*^ did not lead to different IC_50_ values (Table 2), suggesting that the integrity of the Ru compound is most likely maintained (in DMSO solution).

A completely different behavior was observed for **2**_I_2__^*iPr*^. Indeed, a freshly prepared solution of **2**_I_2__^*iPr*^ in DMSO (day 0) gave an IC_50_ value of 48 μM; hence, **2**_I_2__^*iPr*^ was 2 times less cytotoxic than **2**_Cl_2__^*iPr*^. However, if the stock solution of **2**_I_2__^*iPr*^ is used 5 days after its preparation, then an IC_50_ value of 26 μM is obtained; the cytotoxic behavior of **2**_I_2__^*iPr*^ is comparable with that of **2**_Cl_2__^*iPr*^(day 5, Table 1). Even more interestingly, after 7 days in DMSO, **2**_I_2__^*iPr*^ becomes very active, as illustrated by the IC_50_ value of 9.5 μM (day 7, Table 2). Clearly, the initial compound **2**_I_2__^*iPr*^ is slowly converted into a significantly more active species (the new “unknown” species is indeed 5 times more cytotoxic than the original Ru complex against A549 cells). The cytotoxicity of **2**_I_2__^*iPr*^ against MCF7 (breast carcinoma) human cells was evaluated as well, which gave an interesting IC_50_ value of 12 μM with a 7-day-old solution of the complex (Table 2).

The remarkable behavior of **2**_I_2__^*iPr*^ in DMSO, namely its progressive activation (i.e., its increased cytotoxicity), was subsequently investigated with the objective of elucidating the nature of the formed, more cytotoxic species.

### NMR Studies

**Figure 3:**
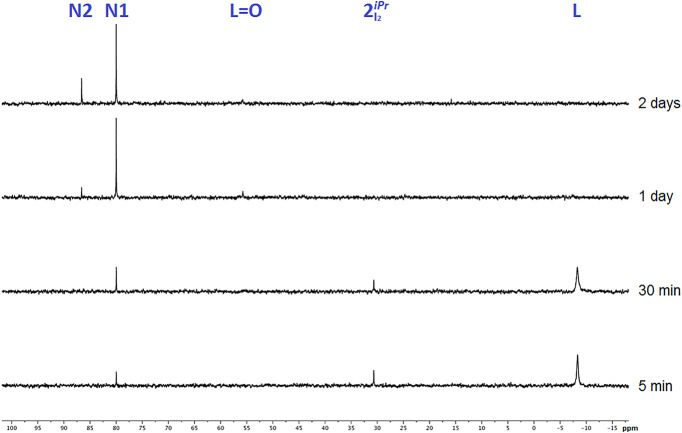
^31^P{^1^H} NMR spectra of complex **2**_I_2__^*iPr*^ in DMSO-*d*_6_ recorded during a period of 48 h, illustrating the progressive formation of new species.

Since the stock solutions of the complexes used for the cytotoxicity assays were prepared in DMSO, the potential modification/alteration of **2**_I_2__^*iPr*^ in this solvent was monitored by ^31^P{^1^H} NMR spectroscopy. The time-dependent corresponding spectra obtained after a period of 48 h are shown in Figure 3.

Already after 5 min, two new peaks, in addition to that corresponding to **2**_I_2__^*iPr*^(+30.7 ppm), are found at −8.3 and +80.0 ppm. The chemical shift at −8.3 ppm is due to the free ligand diisopropyl(1-pyrenyl)phosphane **L** that is released from the complex (in CDCl_3_ δ is found at −8.8 ppm; see the Experimental Section). The second new peak at +80.0 ppm arises from the development of a new species, denoted **N1** (Figure 3; X = I). On the basis of the chemical shift, a cyclometalation reaction involving the pyrenyl ring may be considered to explain the observed low-field value; indeed, a number of related cyclometalated (phosphane)ruthenium(II) complexes have been reported with chemical shifts in the range +70–90 ppm.^41−44^ After 30 min, the intensity of the peak corresponding to **N1** increases, whereas those of **2**_I_2__^*iPr*^ and **L** slightly decrease. After 24 h, two new peaks are detected at +55.7 and +86.6 ppm (Figure 3). The chemical shift at +55.7 ppm is attributable to the oxide of the ligand, i.e. diisopropyl(1-pyrenyl)phosphane oxide (**L=O**). Actually, **L=O** was purposely synthesized by oxidation of **L** with dihydrogen peroxide (see Figures S2 and S3 and Table S4 in the Supporting Information), and its ^31^P{^1^H} NMR spectrum gave a single signal at δ +56.9 ppm in CDCl_3_ (Figure S4). The second new peak at δ +86.6 ppm can be ascribed to another cyclometalated species, denoted **N2** (Figure 3). After 48 h in DMSO, the signals corresponding to **L** and **2**_I_2__^*iPr*^ disappeared completely, while traces of **L=O** could still be seen. The peak due to species **N1** slightly decreased, whereas that of **N2** clearly increased, hence suggesting that **N2** may be formed from **N1**.

**Scheme 2:**
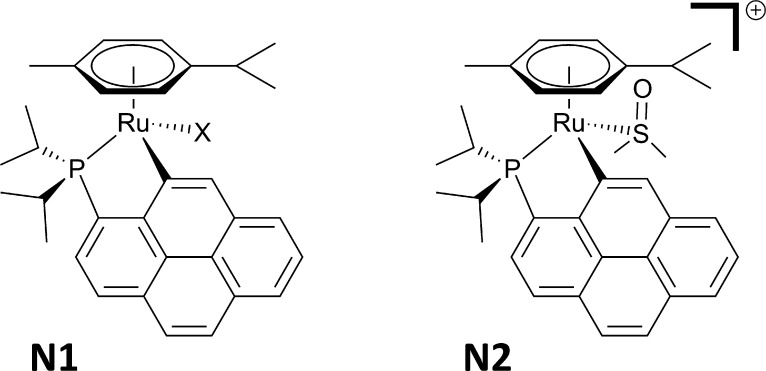
Possible Cyclometalated **N1** and **N2** Species Generated from **2**_Cl_2__^*iPr*^ (X = Cl) and **2**_I_2__^*iPr*^ (X = I)

The same time-course study was carried out for the chlorido complex **2**_Cl_2__^*iPr*^, for comparison. The corresponding NMR spectra in DMSO-*d*_6_ are shown in Figure S5. The same behavior is observed for this compound. An intense broad peak at −8.3 ppm, corresponding to free phosphine **L**, is detected instantly; such peak broadness can be explained by rapid exchange processes between different species in solution. The chemical shift of **2**_Cl_2__^*iPr*^ is found at +36.3 ppm and the oxidized ligand, viz. the phosphane oxide **L=O**, is detected at +55.7 ppm. As for **2**_I_2__^*iPr*^, two signals are observed above +80 ppm, in the “cyclometalated region”. By analogy with **2**_I_2__^*iPr*^ (see above), the peak observed at +80.8 ppm is attributed to **N1** (with X = Cl) and that at +86.6 ppm to **N2**. It can be stressed here that the **N2** species is formed in significantly lower amounts for **2**_Cl_2__^*iPr*^ in comparison to **2**_I_2__^*iPr*^ (see Figure 3 and Figure S5) for the same aging time. Also, in contrast to **2**_I_2__^*iPr*^, the development of a third new species, labeled **N3**, is observed at δ +45.7 ppm (Figure S5). On the basis of its chemical shift, **N3** may be associated with the cationic [RuCl(η^6^-*p*-cymene)(diisopropyl(1-pyrenyl)phosphane)(dmso)]^+^ species resulting from the substitution of one of the chlorido ligands of **2**_Cl_2__^*iPr*^ by a DMSO molecule. Actually, when the time-dependent NMR experiments are carried out for **2**_Cl_2__^*iPr*^ in pure CDCl_3_, the peaks corresponding to **N1** (X = Cl), **N2** and **N3** (X = Cl) are not observed (Figure S6), suggesting that DMSO is actively involved in the formation of these species. Similarly, the time-dependent study for **2**_I_2__^*iPr*^ in pure CDCl_3_ only shows the presence of the starting ruthenium(II) complex together with oxidized ligand **L=O** (Figure S7), in higher amounts than for **2**_Cl_2__^*iPr*^ (Figure S6). It thus appears that stable DMSO-containing species (Scheme 2, right) are generated from the cyclometalated complexes [RuX(η^6^-*p*-cymene)(κ^2^*C*-diisopropyl(1-pyrenyl)phosphane)] (X = Cl, I; Scheme 2, left) in the presence of DMSO and that the process is slower with the chlorido complex (see Study of the Solvation of the Cyclometalated Complexes).

For comparison purposes, ^31^P{^1^H} NMR time-resolved studies in DMSO-*d*_6_ were performed for η^6^-methyl benzoate containing complexes **1**_Cl_2__^*iPr*^ and **1**_I_2__^*iPr*^. The corresponding spectra are shown in Figures S8 and S9. After 24 h, **1**_Cl_2__^*iPr*^, free ligand **L** and **L=O** are observed together with two new compounds that are most likely cyclometalated species (Figure S8). After 48 h, the same species are present in solution. For **1**_I_2__^*iPr*^, the formation of a cyclometalated species is immediately observed (Figure S9). After 24 h, **1**_I_2__^*iPr*^ has completely disappeared; as for the η^6^-*p*-cymene-containing compounds, it appears that the cyclometalation reaction is favored for the iodido complex.

Subsequently, the effect of water on DMSO stock solutions of **2**_Cl_2__^*iPr*^ and **2**_I_2__^*iPr*^ was investigated by ^31^P{^1^H} NMR, as biological studies are performed in aqueous media. Therefore, 24 h aged, concentrated solutions of complexes **2**_Cl_2__^*iPr*^ and **2**_I_2__^*iPr*^ in DMSO-*d*_6_ were mixed with D_2_O, using a DMSO-*d*_6_ to D_2_O ratio of 25:75. Under these conditions, precipitation was observed; the solids were filtered off, and the NMR spectra of the filtrates were recorded. For both samples (i.e. prepared from **2**_Cl_2__^*iPr*^ and **2**_I_2__^*iPr*^), the same single peak was detected in the cyclometalated region, corresponding to that obtained for the **N2** intermediate (see above). Using 48 h aged, concentrated solutions of complexes **2**_Cl_2__^*iPr*^ and **2**_I_2__^*iPr*^, comparable data were obtained; an increase in dissolved sample over time was observed (Figure S10), hence suggesting both the polar nature of the new complex [Ru(η^6^-*p*-cymene)(κ^2^*C*-diisopropyl(1-pyrenyl)phosphane)(dmso)]^+^ in solution and its relatively slow formation process. The formation of an aquo complex of the type [Ru(η^6^-*p*-cymene)(κ^2^C-diisopropyl(1-pyrenyl)phosphane)(H_2_O)]^+^ could not be detected under the conditions applied for these NMR experiments.

### Cyclometalated Compounds

The cyclometalated complexes [RuX(η^6^-*p*-cymene)(κ^2^*C*-diisopropyl(1-pyrenyl)phosphane)] (X = Cl, I) and [Ru(η^6^-*p*-cymene)(κ^2^*C*-diisopropyl(1-pyrenyl)phosphane)(dmso)]^+^ (depicted in Scheme 2) are thus clearly important end species formed in solution from **2**_Cl_2__^*iPr*^ and **2**_I_2__^*iPr*^. Therefore, these compounds were purposely prepared in their pure form.

**Figure 4:**
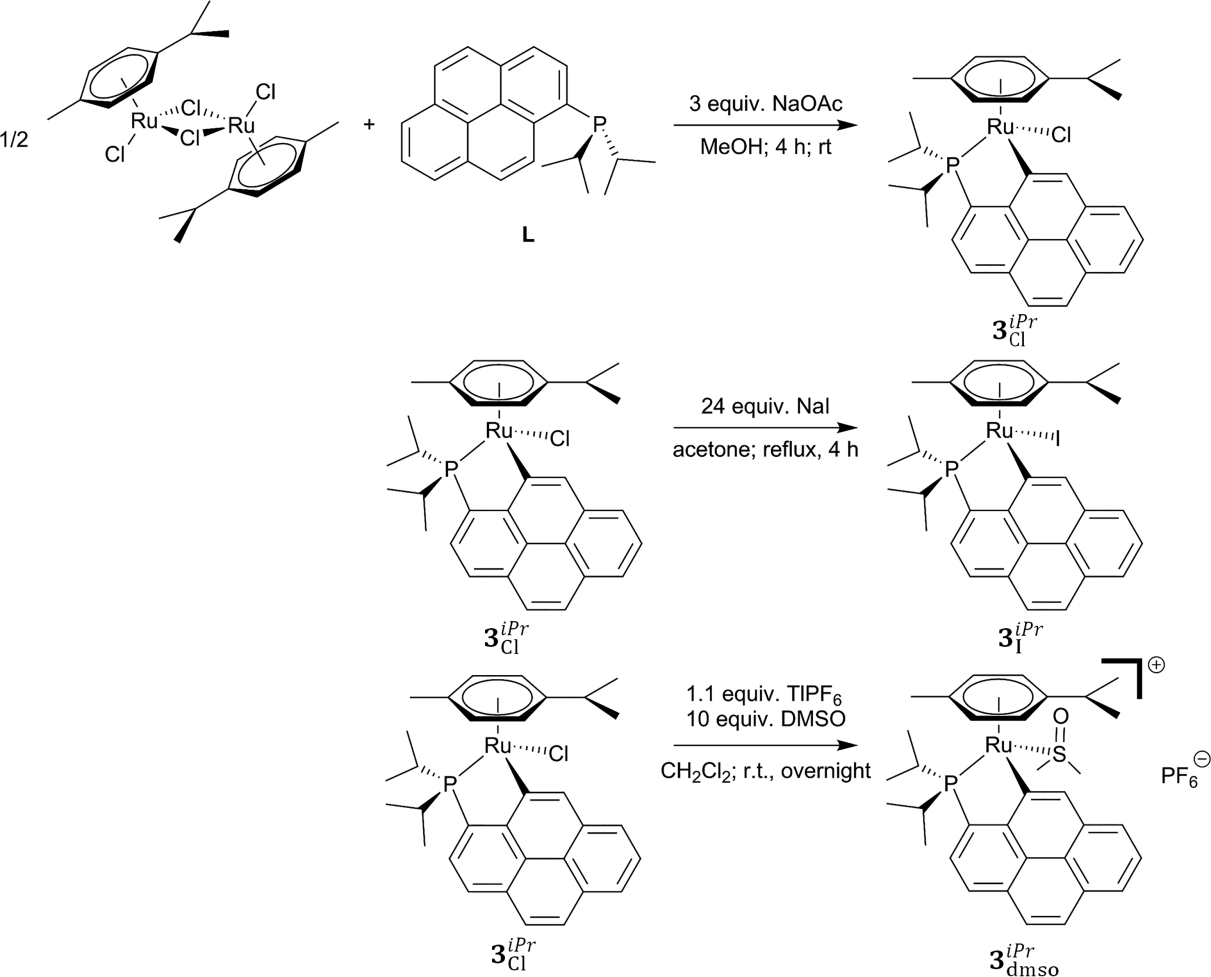
Synthetic procedures to prepare cyclometalated complexes **3**_Cl_^*iPr*^, **3**_I_^*iPr*^, and **3**_dmso_^*iPr*^.

The reaction of dichloro(*p*-cymene)ruthenium(II) dimer with diisopropyl(1-pyrenyl)phosphane (**L**) in methanol in the presence of sodium acetate^42,45^ produces the cycloruthenated complex [RuCl(η^6^-*p*-cymene)(κ^2^*C*-diisopropyl(1- pyrenyl)phosphane)] (**3**_Cl_^*iPr*^) with a yield of 52% (Figure 4). The iodido complex [RuI(η^6^-*p*-cymene)(κ^2^*C*-diisopropyl(1-pyrenyl)phosphane)] (**3**_I_^*iPr*^) is obtained from **3**_Cl_^*iPr*^ with a yield of 82%, by halide exchange in the presence of an excess of sodium iodide in refluxing technical acetone^38^ (Figure 4). Finally, the cationic cyclometalated complex [Ru(η^6^-*p*-cymene)(κ*S*-dmso)(κ^2^*C*-diisopropyl(1-pyrenyl)phosphane)]PF_6_ (**3**_dmso_^*iPr*^) can be prepared in nearly quantitative yield, viz. 95%, by reaction of an excess of DMSO with complex **3**_Cl_^*iPr*^ in the presence of thallium hexafluorophosphate in dichloromethane solution at room temperature (Figure 4).

It can be pointed out here that the ^31^P{^1^H} chemical shifts of **3**_Cl_^*iPr*^, **3**_I_^*iPr*^, and **3**_dmso_^*iPr*^ in CDCl_3_, respectively +80.8, +79.3, and +86.6 ppm (Experimental Section), are in the range of those mentioned in NMR Studies (see Figures 3 and 4), therefore corroborating the assumptions thus made regarding the potential nature of the “unknown” species labeled **N1** (X = Cl, I) and **N2**.

**Figure 5:**
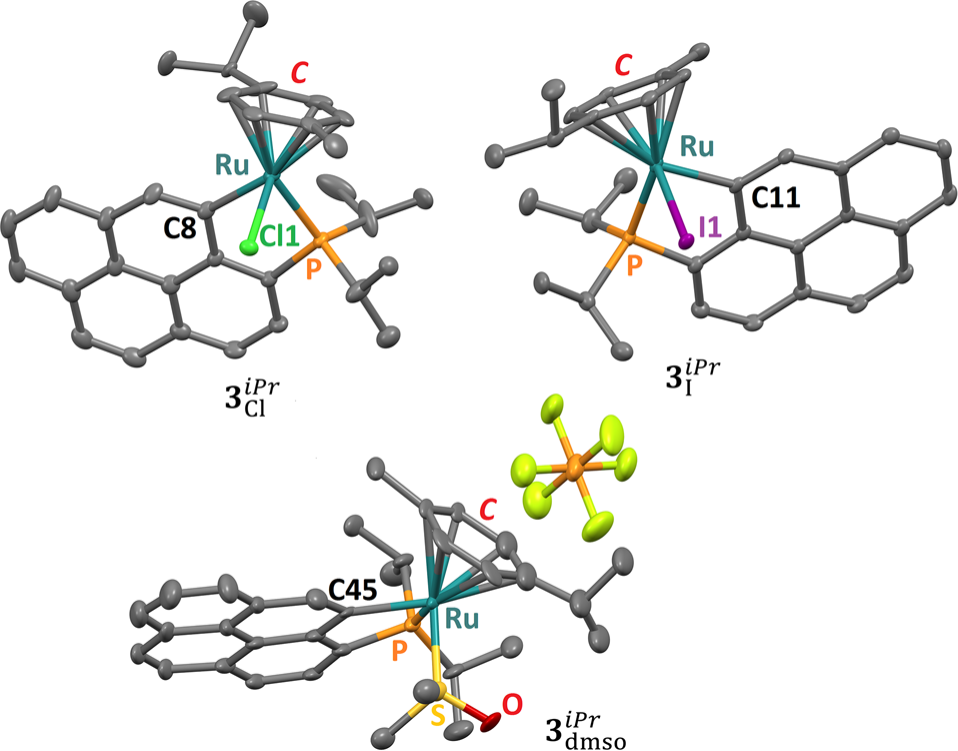
Representation of the crystal structures of cyclometalated **3**_Cl_^*iPr*^, **3**_I_^*iPr*^, and **3**_dmso_^*iPr*^. The atoms bonded to the Ru center are labeled, and ***C*** stands for the centroid of the *p*-cymene ring. Hydrogen atoms are omitted for clarity.

Single crystals of the three cyclometalated compounds, suitable for X-ray diffraction analyses, were obtained (see the Experimental Section); all three complexes crystallize in the monoclinic space group *P*2_1_/*c* (Tables S5 and S7). The crystal structures of the cyclometalated compounds are shown in Figure 5; selected (coordination) bonds and angles are given in Table S6 for **3**_Cl_^*iPr*^ and **3**_I_^*iPr*^ and Table S8 for **3**_dmso_^*iPr*^. The pseudo-octahedral geometry of the Ru center in the three compounds is significantly distorted due to the cyclometalation; for instance, the C–Ru–P angles for all complexes are close to 80° (Tables S5 and S7), while the corresponding X–Ru–P angles in **2**_Cl_2__^*iPr*^ and **2**_I_2__^*iPr*^ are closer to the ideal value, namely 90° (Table S3). The coordination bond lengths are in the range of those found for similar complexes.^34,42^

### Study of the Solvation of the Cyclometalated Complexes

Kinetic studies of the solvation of **3**_Cl_^*iPr*^, **3**_I_^*iPr*^, and **3**_dmso_^*iPr*^ by different solvents were carried out by UV–vis spectroscopy at distinct temperatures and pressures. Saturated solutions of **3**_Cl_^*iPr*^ and **3**_I_^*iPr*^ were prepared in the solvents to be investigated: namely, DMSO, DMSO saturated with NaCl, *n*-butanol (chosen for comparative purposes), and water.

Generally, suspensions were obtained, which were sonicated for 5–6 min and filtered over glass wool (to eliminate any remaining insolubilized compound); the filtrates were then transferred into UV–vis cells that were subsequently placed in a thermostated UV–vis spectrophotometer. After temperature equilibration, time-resolved spectra were collected at different time scales and intervals to warrant total conversion (4–6 half-lives) to the expected solvato complexes.

**Figure 6:**
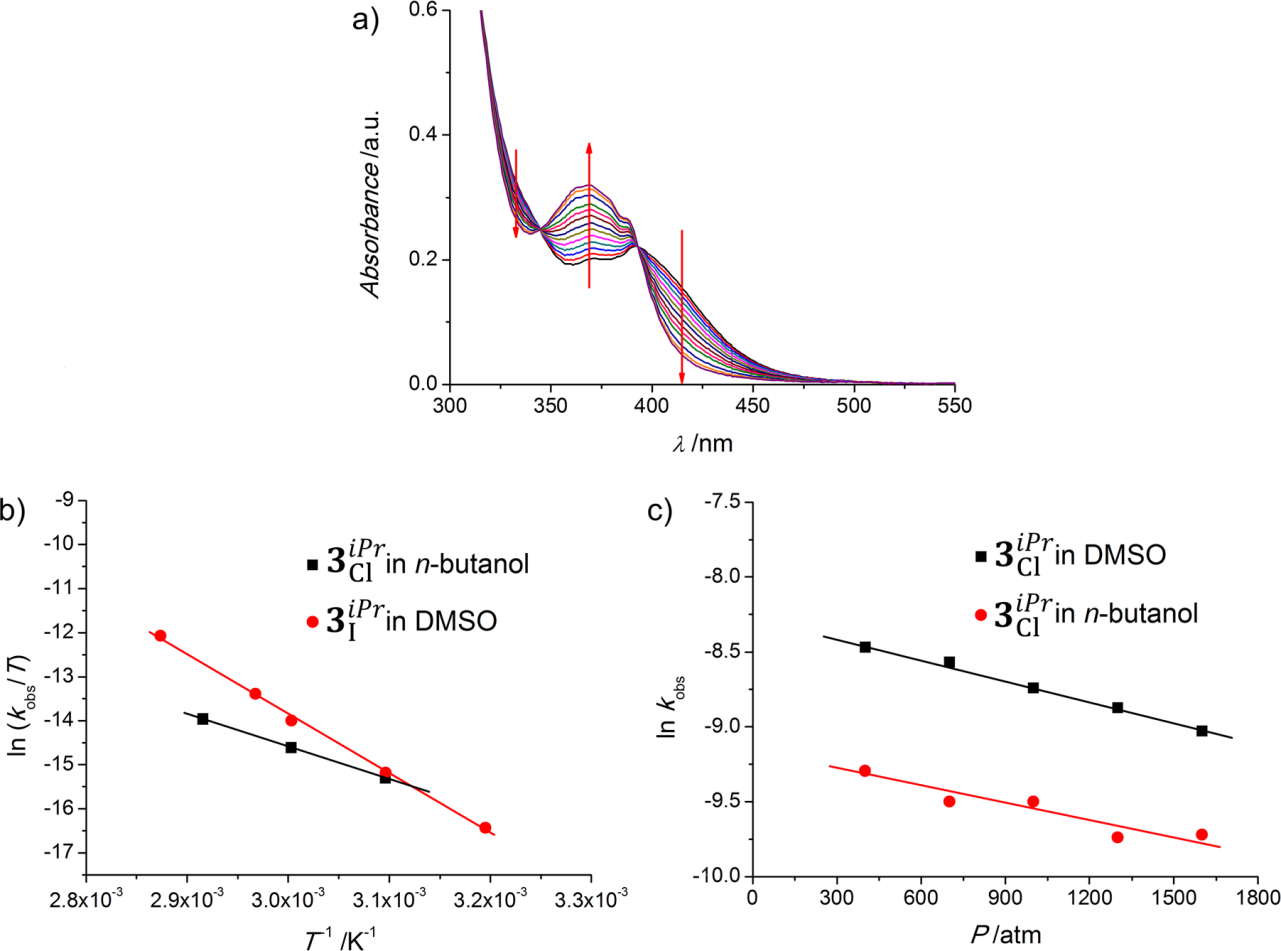
(a) Representative set of time-resolved spectral changes observed for a solution of **3**_Cl_^*iPr*^ in NaCl-saturated DMSO at 70 °C for 10 h. (b) Selected Eyring plots of the temperature dependence of *k*_obs_ for **3**_Cl_^*iPr*^ in *n*-butanol (black ■) and **3**_I_^*iPr*^ in DMSO (red ●). (c) Selected plots of the pressure dependence of ln *k*_obs_ for **3**_Cl_^*iPr*^ in DMSO (black ■) and **3**_Cl_^*iPr*^ in *n*-butanol (red ●).

The first-order rate constants obtained for all solvation experiments carried out are given in Tables S9 and S10. These constants were determined by fitting the time-resolved spectral data with Specfit^46^ or ReactLab,^47^ considering an A → B process. It should be noted that, upon dilution of the initial solution (1:1 and 1:5 dilutions), no variations in rate constants were observed, therefore indicating the nonactuation of multinuclear species during the process. Moreover, when DMSO saturated with NaCl was used (Figure 6a), no noticeable differences in *k*_obs_ values were found, hence suggesting the nonequilibrium nature of the solvation process under the conditions applied. Examples of the temperature and pressure dependence of the *k*_obs_ values for representative systems are shown in Figure 6b,c, respectively. The values determined for the thermal and pressure activation parameters for a series of solvation experiments with **3**_Cl_^*iPr*^, **3**_I_^*iPr*^, and **3**_dmso_^*iPr*^ are given in Table 3; the extrapolated *k*_obs_ and 6 × *t*_1/2_ values at 37 °C (310 K) are also given in Table 3. It should be noted that the data provided for **3**_Cl_^*iPr*^ in water were obtained from the time-resolved appearance of definite UV–vis spectra, resulting from the progressive aquation of the chlorido ligand producing polar ionic species, most likely [Ru(η^6^-*p*-cymene)(κ^2^*C*-diisopropyl(1-pyrenyl)phosphane)(H_2_O)]^+^, exhibiting higher (but still limited) water solubility in comparison to **3**_Cl_^*iPr*^ (see below).

**Table 3:** Summary of the Kinetic, Thermal, and Pressure Activation Parameters for the Solvation of **3**_Cl_^*iPr*^, **3**_I_^*iPr*^, and **3**_dmso_^*iPr*^ with Various Solvents^a^

compound	solvolysis by	10^5^ × ^310^*k*_obs_/s^–1^ (6 × *t*_1/2_/days)	Δ*H*^⧧^/kJ mol^–1^	Δ*S*^⧧^/J K^–1^ mol^–1^	Δ*V*^⧧^/cm^3^ mol^–1^
**3**_Cl_^*iPr*^	DMSO^d^	0.35 (330)	85 ± 5	–78 ± 14	6.2 ± 0.6
	*n*-butanol	2.4 (48)	63 ± 1	–133 ± 1	12 ± 2
	H_2_O^b^	5.2 (22)	52 ± 2	–162 ± 5	nd^c^
					
**3**_I_^*iPr*^^e^	DMSO^d^	1.6 (72)	113 ± 3	25 ± 8	13 ± 0.5
	*n*-butanol	12 (9.6)	62 ± 2	–123 ± 5	20 ± 1
					
**3**_dmso_^*iPr*^	H_2_O^f^	2.0 (58)	71 ± 4	–110 ± 13	–14 ± 2

a [Complex] = 10–50 μM.

b **3**_Cl_^*iPr*^ is poorly soluble in water; the values are derived from the rate of appearance of definite UV–vis spectra.

c Not determined.

d DMSO containing 0.005% of water was used ([H_2_O] = 2.77 × 10^–3^ M).

e **3**_I_^*iPr*^ is completely insoluble in water.

f Experiments performed using a saturated aqueous solution; [**3**_dmso_^*iPr*^] ≈ 10 μM (such a low concentration had to be used due to the very low solubility of this compound in water).

**Figure 7:**
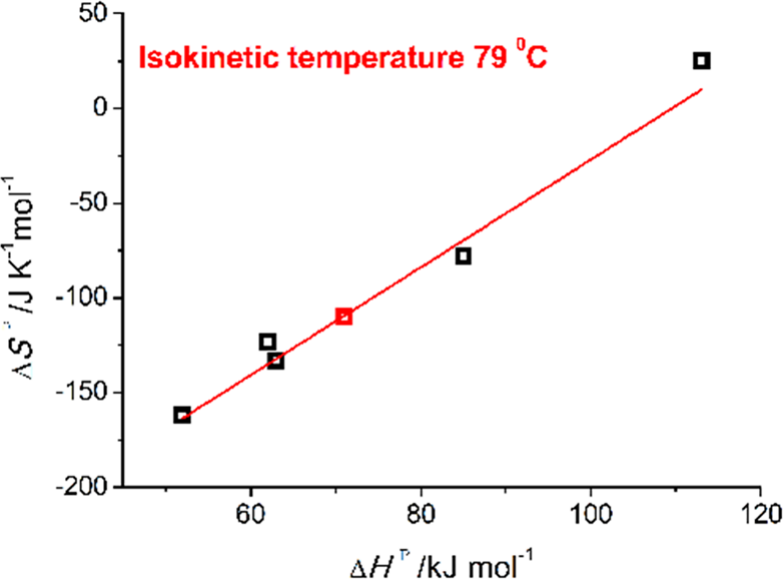
Isokinetic (79 °C) compensation plot (black squares) for the thermal activation parameters obtained for the solvolysis reactions of **3**_Cl_^*iPr*^ and **3**_I_^*iPr*^ (Table 3). The red square corresponds to the aquation of **3**_dmso_^*iPr*^.

Wide ranges of enthalpies (52–113 kJ mol^–1^) and entropies (−162 to +25 J K^–1^ mol^–1^) of activation are observed for these solvation processes (Table 3; compounds **3**_Cl_^*iPr*^ and **3**_I_^*iPr*^). These data suggest the operation of a rather uniform substitution mechanism showing a complete compensation between the activation entropies and enthalpies (Figure 7, black squares), with a clear isokinetic temperature, i.e. 79 °C, outside of the range experimentally used (**3**_Cl_^*iPr*^, Table S9; **3**_I_^*iPr*^, Table S6).^48−50^ The process is therefore explained by a rather pure interchange mechanism with a certain degree of dissociation and ordering, as indicated by the general trend of the set of Δ*V*^⧧^ (>0) and Δ*S*^⧧^ (<0) (see Table 3). On consideration that a neat charge separation is taking place during the process and that DMSO is less polar than *n*-butanol,^51^ the larger negative values of Δ*S*^⧧^ for the latter can be explained by solvent ordering of charge formation. Similarly, the more positive values of Δ*V*^⧧^ for *n*-butanol can be justified by a higher number of solvent molecules involved in the process. Furthermore, the more positive Δ*V*^⧧^ values for iodido complex **3**_I_^*iPr*^ in comparison with chlorido complex **3**_Cl_^*iPr*^ (Table 3) suggest that higher volume changes on charge separation occur with the less polar compound, as one would expect.^52^ The results obtained are rather surprising, given the accepted associativeness of substitution mechanisms observed with ruthenium(II) complexes,^53,54^ which is normally accepted as a positive factor for biological applications. The important covalent character of the M–L bonds in organometallic **3**_Cl_^*iPr*^ and **3**_I_^*iPr*^ most likely decreases the Lewis acidity of the metal center, by comparison with classical Werner-type complexes; consequently, the associative demand in the substitution process is reduced. Similar features were observed for substitution reactions with organometallic platinum(IV)^55−57^ and platinum(II) compounds.^58,59^

The behavior of **3**_dmso_^*iPr*^ in water was investigated next. As **3**_dmso_^*iPr*^ is very poorly soluble in water, 10 μm solutions of this compound were prepared by sonication and the higher temperatures given in Table S9, i.e. 40–80 °C, were used (since the compound was hardly water soluble at room temperature). Despite these solubility issues, the large values of the extinction coefficients of the absorptions in the range of 350–400 nm allowed us to record satisfactory time-resolved spectra, even at variable pressures. The values of the kinetic and activation parameters obtained (with larger associated errors, compared with those of **3**_Cl_^*iPr*^ and **3**_I_^*iPr*^) are given in Table 3. The data point shown in Figure 7 (red square) suggests that a similar substitution mechanism for the DMSO/water exchange is taking place in **3**_dmso_^*iPr*^. However, the values of both the volume and entropy of activation are negative, hence suggesting that the ordering in this reaction is associated with a volume decrease; for **3**_dmso_^*iPr*^, no charge variation is occurring during the DMSO/water exchange, in contrast to the halide/DMSO exchanges in **3**_Cl_^*iPr*^ and **3**_I_^*iPr*^. As a result, the ordering and contraction go in the same direction in the case of **3**_dmso_^*iPr*^. Furthermore, from the data obtained, a very interesting shift to the associative activation side of the interchange process seems to apply for these cationic ruthenium(II) species.

**Figure 8:**
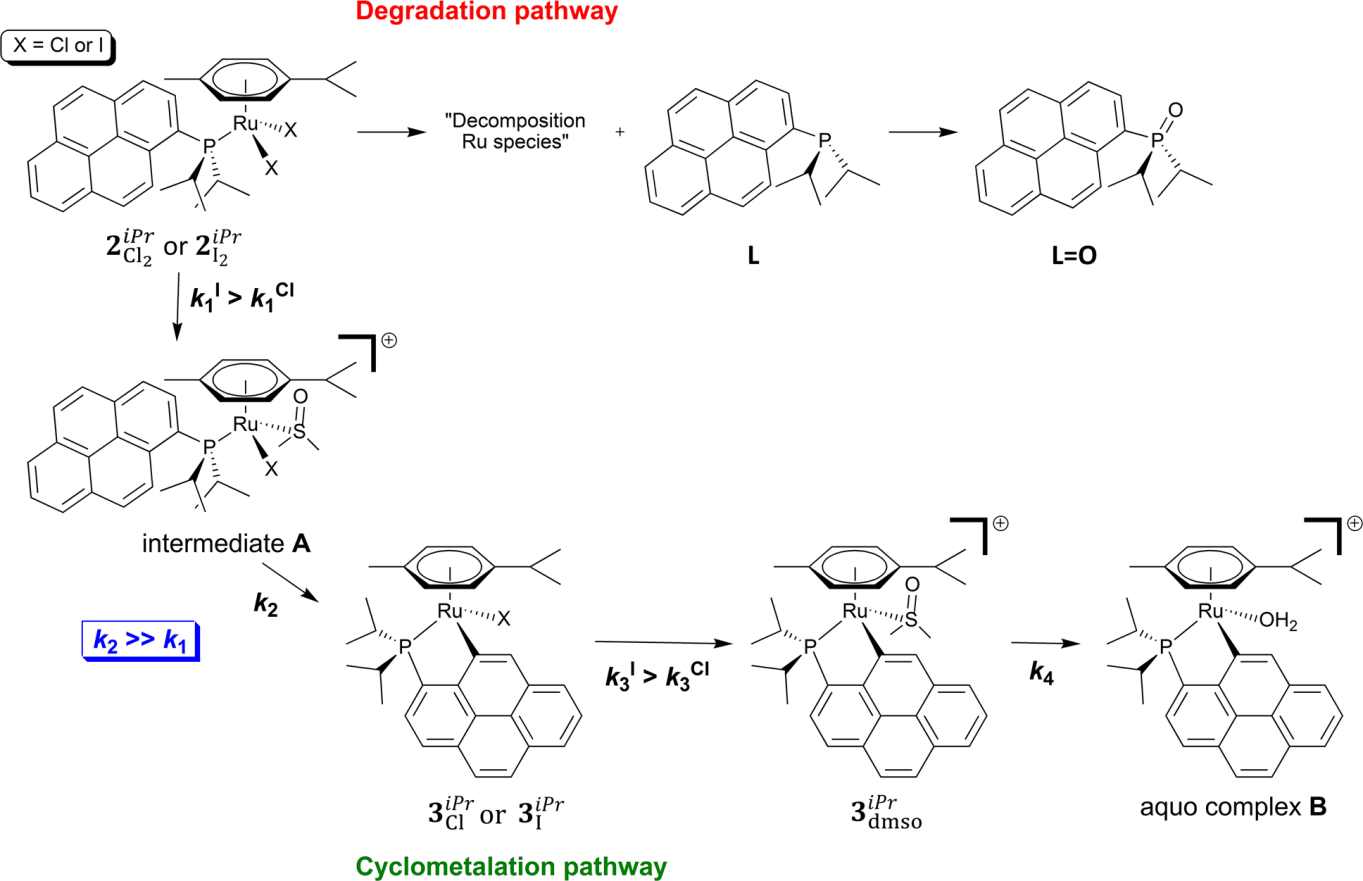
Representation of the different species generated in solution upon dissolution of **2**_CI_2__^*iPr*^ or **2**_I_2__^*iPr*^ in the presence of DMSO.

**Figure 9:**
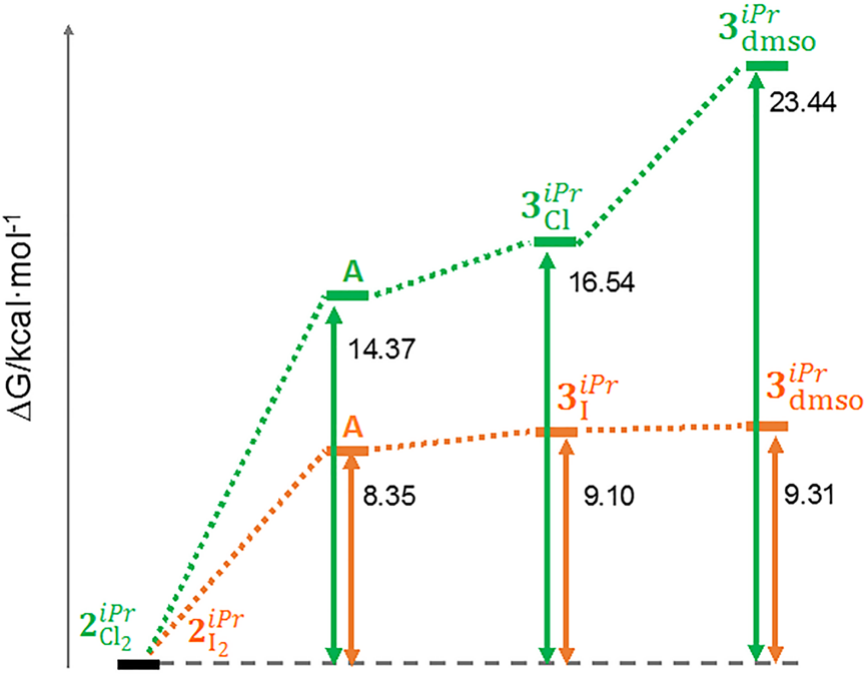
Energy profiles (in kcal mol^–1^) for intermediate **A** → **3**_X_^*iPr*^ → **3**_dmso_^*iPr*^ processes: orange, X = I; green, X = Cl.

In summary, once in solution, and especially in the presence of DMSO, compounds **2**_CI_2__^*iPr*^ and **2**_I_2__^*iPr*^ undergo a series of transformations, which are illustrated in Figure 8. Partial degradation of the compounds is observed, as the free phosphane ligand **L** is detected together with its oxidized form, namely phosphane oxide **L=O** (Figure 9, degradation pathway). In the absence of DMSO, cyclometalated species are not generated; therefore, this solvent clearly plays an important role in the transformation pathway. It is believed that the first step consists of the substitution of an halido ligand by a DMSO molecule (*k*_1_), converting **2**_CI_2__^*iPr*^ or **2**_I_2__^*iPr*^ into intermediate **A**. This intermediate is readily converted into **3**_Cl_^*iPr*^ or **3**_I_^*iPr*^(*k*_2_; Figure 8), through a cyclometalation reaction with DMSO acting as a Lewis base, in the so-called concerted metalation–deprotonation (CMD) mechanism.^60,61^ It is indeed proposed that coordinated DMSO makes an intramolecular hydrogen bond with the pyrenyl group, hence fostering the cyclometalation (Figure S11). **3**_Cl_^*iPr*^ and **3**_I_^*iPr*^ then undergo substitution of the second halido ligand by a DMSO molecule, producing the cationic cyclometalated compound **3**_dmso_^*iPr*^ (*k*_3_), which is finally aquated (i.e. DMSO is replaced with water, *k*_4_; Figure 8).

It should be noted that the *k*_1_ and *k*_2_ rate constants could not be determined. First, the degradation path masks the determination of the value of *k*_1_. Second, as *k*_2_ is clearly significantly higher than *k*_1_ (as indicated by the ^31^P{^1^H} NMR experiments), once **2**_Cl_2__^*iPr*^ and **2**_I_2__^*iPr*^ are converted into their respective intermediates **A**, the subsequent cyclometalation reaction to yield **3**_Cl_^*iPr*^ and **3**_I_^*iPr*^ is immediate (*k*_2_ ≫ *k*_1_), not allowing a determination of the value of *k*_2_ under the experimental conditions applied in the present study. It can be pointed out here that *k*_2_^I^ is most likely superior to *k*_2_^Cl^; indeed, NMR studies with **2**_Cl_2__^*iPr*^ have shown (see above) that a species labeled **N3** was forming in DMSO (δ +45.7 ppm; Figure S5), which may be ascribed to the chlorido intermediate **A** (X = Cl; Figure 8). In the case of **2**_I_2__^*iPr*^, such a species was not observed by NMR (Figure 3), suggesting that when it is formed it is very rapidly converted into compound **3**_I_^*iPr*^ (hence *k*_2_^I^ > *k*_2_^Cl^). The rate constants for the conversion of **3**_Cl_^*iPr*^ and **3**_I_^*iPr*^ into **3**_dmso_^*iPr*^, respectively *k*_3_^Cl^ and *k*_3_^I^, could be obtained (see Table 3), which showed that the iodido to DMSO substitution was almost 4.6 times faster than the chlorido to DMSO substitution (*k*_3_^I^ > *k*_3_^Cl^; Table 3). It should be stressed here that one may expect an analogous trend for the first halido to DMSO substitution, namely *k*_1_^I^ > *k*_1_^Cl^ (**2**_Cl_2__^*iPr*^ or **2**_I_2__^*iPr*^ → intermediate **A**; Figure 8); though additional in-depth studies are required to confirm this. Finally, the rate constant for the aquation of **3**_dmso_^*iPr*^, namely *k*_4_ (**3**_dmso_^*iPr*^ → aquo complex **B**; Figure 8), was also determined (Table 3), showing that it was a relatively slow process.

The changes in free energy (Δ*G*°) have been calculated for intermediates **A** (for X = Cl and X = I), **3**_Cl_^*iPr*^ and **3**_I_^*iPr*^, and **3**_dmso_^*iPr*^ (Figure 8), with respect to the corresponding starting compounds **2**_Cl_2__^*iPr*^ and **2**_I_2__^*iPr*^; these are shown in Figure 9. The energy profile is clearly more favorable when X = I (orange profile in Figure 9), corroborating the experimental data. For instance, the cyclometalation step (intermediate **A** → **3**_X_^*iPr*^) costs only 0.75 kcal mol^–1^ for X = I, whereas it is 2.17 kcal mol^–1^ for X = Cl. However, it can be noted that in both cases the cyclometalation is energetically inexpensive (although more feasible for X = I). The step **3**_X_^*iPr*^ → **3**_dmso_^*iPr*^ is costless for X = I (energy difference of only 0.21 kcal mol^–1^), while a difference in energy of 6.9 kcal mol^–1^ is found between **3**_Cl_^*iPr*^ and **3**_dmso_^*iPr*^ (Figure 9).

It is noteworthy to stress that the geometry optimization of intermediate **A** (Figure 8) shows a clear orientation of the oxygen atom of the DMSO ligand toward the hydrogen atom involved in the cyclometalation of the pyrenyl group (the O···H distances being 2.18 and 2.38 Å for X = Cl, I, respectively), as proposed in Figure S11, thus confirming the crucial involvement of the solvent in the cyclometalation pathway.

### Cytotoxicity Behaviors of Cyclometalated Compounds **3**_Cl_^*iPr*^, **3**_I_^*iPr*^, and **3**_dmso_^*iPr*^

IC_50_ values were then determined for compounds **3**_Cl_^*iPr*^, **3**_I_^*iPr*^, and **3**_dmso_^*iPr*^ at increasing “aging times”, using A549 (lung adenocarcinoma) human cells for comparison with the time-dependent IC_50_ values obtained for **2**_Cl_2__^*iPr*^ and **2**_I_2__^*iPr*^ for the same cell line (see Table 2). The results achieved are given in Table 4. It can be pointed out that a concentration range of 0.4–50 μM was used for these assays, because precipitation of the cyclometalated ruthenium compounds was observed at concentrations above 100 μM.

**Table 4:** Half-Maximum Inhibitory Concentrations^a^ (IC_50_, μM) of Compounds **3**_Cl_^*iPr*^, **3**_I_^*iPr*^, and **3**_dmso_^*iPr*^ for A549 (Lung Adenocarcinoma) Human Cells, after Incubation for 24 h (Day 0) and after 1, 2, and 7 days of Aging (before IC_50_ Determination)

compound	day 0^b^	day 1	day 2	day 7
**3**_Cl_^*iPr*^	2.26 ± 0.34	2.77 ± 0.38	4.92 ± 0.59	2.67 ± 1.61
**3**_I_^*iPr*^	5.82 ± 1.89	5.58 ± 0.47	5.61 ± 2.16	4.32 ± 2.54
**3**_dmso_^*iPr*^	1.72 ± 0.67	2.47 ± 0.57	2.56 ± 0.44	2.34 ± 0.61

a The results are expressed as mean values ± SD out of three independent experiments.

b Day 0 corresponds to the first determination of IC_50_, after 24 h of incubation with freshly prepared stock solutions of the compounds.

Remarkably, in each case, comparable IC_50_ values were obtained for the samples with different “aging times”. These data indicate that the cyclometalated complexes remain unchanged in solution (in contrast to **2**_Cl_2__^*iPr*^ and **2**_I_2__^*iPr*^). The cytotoxic activities of compounds **3**_Cl_^*iPr*^, **3**_I_^*iPr*^, and **3**_dmso_^*iPr*^ are clearly better than those of the “parent compounds” **2**_Cl_2__^*iPr*^ and **2**_I_2__^*iPr*^. It is interesting to note that the cytotoxic activities of **3**_dmso_^*iPr*^ are comparable to those of **3**_Cl_^*iPr*^ and **3**_I_^*iPr*^, suggesting that replacement of the halide by DMSO has no effect on the biological properties. Surprisingly, compound **3**_I_^*iPr*^ is less cytotoxic than **3**_Cl_^*iPr*^; from the IC_50_ data obtained with **2**_Cl_2__^*iPr*^ and **2**_I_2__^*iPr*^ (see Table 2), one would have expected compound **3**_I_^*iPr*^ to be more active than **3**_Cl_^*iPr*^. This can be justified by the significantly higher conversion rates for **2**_I_2__^*iPr*^ to form **3**_I_^*iPr*^ and ultimately **3**_dmso_^*iPr*^ (see above, Figure 8; *k*_1_, *k*_2_, and *k*_3_). Hence, the formation of the active cyclometalated species is significantly faster with **2**_I_2__^*iPr*^; thus, the little amounts of **3**_I_^*iPr*^ (and of **3**_dmso_^*iPr*^) progressively generated in solution are enough to give the increasing cytotoxicity observed for **2**_I_2__^*iPr*^ at days 5 and 7 (Table 2). Conversion of **2**_CI_2__^*iPr*^ to a cyclometalated species is much slower; therefore, evolution of the IC_50_ value is not observed within a period of 7 days (see Table 2). It can be pointed out that the observed IC_50_ values, ranging from 1.72 to 5.82 μM, are comparable to data reported in the literature for various types of piano-stool ruthenium(II) complexes.^62−65^ However, it can be stressed that the IC_50_ values for **3**_Cl_^*iPr*^, **3**_I_^*iPr*^ and **3**_dmso_^*iPr*^, given in Table 2, were determined after 24 h of incubation with cells, whereas most of the IC_50_ values found in the literature were obtained after a drug-exposure time of 48, 72, or 96 h;^66−68^ hence, the low-micromolar IC_50_ values achieved after 24 h of incubation with **3**_CI_^*iPr*^, **3**_I_^*iPr*^, and **3**_dmso_^*iPr*^ indicate that they are highly cytotoxic. Notable activities after 24 h of incubation have been described for tethered,^69^ acylpyrazolonato-containing,^70^ or amino-oxime-based half-sandwich ruthenium(II) complexes,^71^ but **3**_Cl_^*iPr*^, **3**_I_^*iPr*^, and **3**_*iPr*_^dmso^ are comparatively more efficient.

### Dimethyl(1-pyrenyl)phosphane versus Diisopropyl(1-pyrenyl)phosphane

As mentioned in the Introduction, the use of the new ligand diisopropyl(1-pyrenyl)phosphane (**L**) originates from previous studies, which have shown that P-ligands of the type PR^1^R^2^(1-pyrenyl), which give rise to [RuX_2_(η^6^-arene)(PR^1^R^2^(1-pyrenyl))] compounds, exhibit remarkable cytotoxic properties; in particular, the chlorido Ru complex with the ligand dimethyl(1-pyrenyl)phosphane and η^6^-methyl benzoate, namely [RuCl_2_(η^6^-methyl benzoate)(dimethyl(1-pyrenyl)phosphane)], exhibited IC_50_ values in the low micromolar range for various cancer cell lines.^35^ The aim of the investigation presented herein was to examine the effect of the halide, viz. iodide versus chloride, on the cytotoxic properties of the corresponding Ru complexes bearing a sterically hindered P-ligand (for instance, to favor aquation, namely replacement of the halides by water molecules). Surprisingly, the cytotoxicity data obtained for compounds **1**_Cl_2__^*iPr*^, **1**_I_2__^*iPr*^, **2**_Cl_2__^*iPr*^, and **2**_I_2__^*iPr*^ were low (Table 1), especially for **1**_Cl_2__^*iPr*^ and **1**_I_2__^*iPr*^ (which are not active at all), which include the η^6^-methyl benzoate ligand. Indeed, in the earlier study, this η^6^-arene moiety generated the most cytotoxic agent of the series described therein, namely [RuCl_2_(η^6^-methyl benzoate)(dimethyl(1-pyrenyl)phosphane)] (**1**_Cl_2__^*Me*^).^35^ For this reason, we decided to synthesize the iodido complexes of the ligand dimethyl(1-pyrenyl)phosphane,^35^ with both η^6^-methyl benzoate and η^6^-*p*-cymene, for comparison purposes. Hence, the iodide complexes [RuI_2_(η^6^-methyl benzoate)(dimethyl(1-pyrenyl)phosphane)] (**1**_I_2__^*Me*^) and [RuI_2_(η^6^-*p*-cymene)(dimethyl(1-pyrenyl)phosphane)] (**2**_I_2__^*Me*^) (Scheme S2) were prepared by halide exchange of their chlorido counterparts (chlorido Ru complexes are reported in ref (35)).

The solid-state structures of **1**_I_2__^*Me*^ and **2**_I_2__^*Me*^ could be obtained (Figure S12). **1**_I_2__^*Me*^ crystallizes in the monoclinic space group *P*2_1_/*c* and **2**_I_2__^*Me*^ in the orthorhombic space group *P*2_1_2_1_2_1_ (Table S11). Selected coordination bond lengths and angles are given in Table S12. The structural data of both compounds are comparable with those of **1**_Cl_2__^*iPr*^, **1**_I_2__^*iPr*^, **2**_Cl_2__^*iPr*^, and **2**_I_2__^*iPr*^, and are typical for such coordination geometry.^35,39,40^

IC_50_ values were then determined for **1**_I_2__^*Me*^ and **2**_I_2__^*Me*^ using three different cell lines: namely, A549 (lung adenocarcinoma), SW620 (colorectal adenocarcinoma) and MCF7 (breast carcinoma). The data are given in Table 5. **1**_I_2__^*Me*^ and **2**_I_2__^*Me*^ are significantly more cytotoxic than **1**_Cl_2__^*iPr*^, **1**_I_2__^*iPr*^, **2**_Cl_2__^*iPr*^, and **2**_I_2__^*iPr*^; moreover, no delay in their biological activity was observed (data not shown), in deep contrast to **2**_I_2__^*iPr*^. In fact, **1**_I_2__^*Me*^ and **2**_I_2__^*Me*^ are behaving as their chlorido counterparts, i.e. [RuCl_2_(η^6^-methyl benzoate)(dimethyl(1-pyrenyl)phosphane)] (**1**_Cl_2__^*Me*^) and [RuCl_2_(η^6^-*p*-cymene)(dimethyl(1-pyrenyl)phosphane)] (**2**_Cl_2__^*Me*^), although less efficiently.^35^ Therefore, replacement of the chlorides by iodides does not seem to produce drastic changes regarding the biological properties of the corresponding Ru complexes; only for cell line A549 are iodido **1**_I_2__^*Me*^ and **1**_I_2__^*Me*^ respectively 2 and 3 times more active than their chlorido counterparts (Table 5). It is important to note as well that, as observed in the earlier study,^35^ η^6^-methyl benzoate containing **1**_I_2__^*Me*^ is clearly more effective than η^6^-*p*-cymene containing **2**_I_2__^*Me*^ (Table 5). In contrast, with diisopropyl(1-pyrenyl)phosphane as the P-ligand, η^6^-*p*-cymene containing **2**_Cl_2__^*iPr*^ and **2**_I_2__^*iPr*^ affect the viability of the cells, whereas η^6^-methyl benzoate containing **1**_Cl_2__^*iPr*^ and **1**_I_2__^*iPr*^ are not active at all (Table 1).

**Table 5:** Half-Maximum Inhibitory Concentrations^a^ (IC_50_, μM) of Compounds **1**_I_2__^*Me*^ and **2**_I_2__^*Me*^ and their Chlorido Counterparts, Respectively [RuCl_2_(η^6^-methyl benzoate)(dimethyl(1-pyrenyl)phosphane)] (**1**_Cl_2__^*Me*^) and [RuCl_2_(η^6^-*p*-cymene)(dimethyl(1-pyrenyl)phosphane)] (**2**_Cl_2__^*Me*^),^35^ for A549 (Lung Adenocarcinoma), SW620 (Colorectal Adenocarcinoma) and MCF7 (Breast Carcinoma) Human Cells, after Incubation for 24 h

compound	A549	SW620	MCF7
**1**_I_2__^*Me*^	2.6 ± 0.4	9 ± 2	6 ± 1
**1**_Cl_2__^*Me*^^b^	5.0 ± 0.6	1.9 ± 0.1	5.1 ± 1.6
**2**_I_2__^*Me*^	6.3 ± 0.4	16 ± 4	17 ± 6
**2**_Cl_2__^*Me*^^b^	17.2 ± 0.5	6.5 ± 0.8	9.7 ± 0.1

a The results are expressed as mean values ± SD out of three independent experiments.

b Compounds described in ref (35).

These data again demonstrate the significance of the nature of the P-ligand on changing from dimethyl(1-pyrenyl)phosphane to the bulkier diisopropyl(1-pyrenyl)phosphane (that is from methyl to isopropyl substituents); the mechanism of action of the resulting Ru complexes is completely modified. When the bulkier ligand, namely ligand **L** (bearing two isopropyl groups), is combined with a bulkier η^6^ ring, namely *p*-cymene (1-methyl-4-isopropylbenzene), the corresponding complexes undergo a series of transformations in DMSO, leading to the formation of cytotoxic cyclometalated compounds. With the less sterically hindered ligand dimethyl(1-pyrenyl)phosphane, this cyclometalation pathway is minor, the noncyclometalated complexes obtained also being very cytotoxic, especially if the η^6^ ring is the less bulky η^6^-methyl benzoate. It can also be mentioned that ^31^P{^1^H} NMR studies revealed that cyclometalated species do not seem to be formed in DMSO with complex **1**_I_2__^*Me*^, which is mostly preserved in this solvent (Figure S13). Remarkably, for *p-*cymene containing **2**_I_2__^*Me*^, a slow cyclometalation reaction seems to take place; **2**_I_2__^*Me*^, however, remains the main species present in solution, even after 48 h (Figure S14). It can be pointed out that the cyclometalation is again favored in the presence of the bulkier *p*-cymene ring. This cyclometalation reaction with **2**_I_2__^*Me*^ will be investigated in the future.

In summary, the combination (i) of nonbulky ligands (phosphane + η^6^ ring) leads to typical piano-stool complexes and (ii) of bulky ligands generates cyclometalated complexes, both families of ruthenium(II) compounds exhibiting high cytotoxic activities.

## Conclusions

Following recent studies with ruthenium(II) complexes of the type [Ru(η^6^-arene)X_2_(P(1-pyrenyl)R^2^R^3^)] (with η^6^-arene = benzoate, *p*-cymene and R^2^, R^3^ = methyl, phenyl) that showed interesting cytotoxic behaviors,^35^ four new members of this family of piano-stool complexes have been prepared, namely **1**_Cl_2__^*iPr*^, **1**_I_2__^*iPr*^, **2**_Cl_2__^*iPr*^, and **2**_I_2__^*iPr*^, with the objective of investigating the role played by the bulkiness of the phosphane ligand (R^2^ = R^3^ = isopropyl) as well as the nature of the coordinated anions (X = Cl, I) on the cytotoxic properties. Unexpectedly, complexes **1**_Cl_2__^*iPr*^, **1**_I_2__^*iPr*^, **2**_Cl_2__^*iPr*^, and **2**_I_2__^*iPr*^ were not as biologically active as the previously reported compounds **1**_Cl_2__^*Me*^ and **2**_Cl_2__^*Me*^,^35^ bearing a less bulky ligand (i.e., R^2^ = R^3^ = methyl instead of isopropyl). However, compound **2**_I_2__^*iPr*^ exhibited a striking behavior, since it was observed that its cytotoxic activity was increasing over time (viz. aged DMSO solutions of **2**_I_2__^*iPr*^ gave lower IC_50_ values in comparison to freshly prepared solutions).

This surprising and very interesting feature should be due to a transformation of **2**_I_2__^*iPr*^ in solution. Comprehensive studies were therefore carried out to try to elucidate the origin of the observed lag time in activity. It was found that **2**_I_2__^*iPr*^ and **2**_CI_2__^*iPr*^ were undergoing a series of transformations in DMSO (not in CHCl_3_), ultimately producing stable cyclometalated species (involving the pyrene ring): i.e. compounds **3**_Cl_^*iPr*^, **3**_I_^*iPr*^ , and **3**_dmso_^*iPr*^. The cyclometalation reaction was significantly faster with iodido **2**_I_2__^*iPe*^ in comparison to chlorido **2**_Cl_2__^*iPr*^. Notably, compounds **1**_Cl_2__^*iPr*^ and **1**_I_2__^*iPr*^, containing η^6^-methyl benzoate (whereas **2**_Cl_2__^*iPr*^ and **2**_I_2__^*iPr*^ have η^6^-*p*-cymene), only showed the formation of traces of cyclometalated complexes. Hence, cyclometalation is favored when both the η^6^-arene ring and the phosphane ligand are bulky; moreover, the cyclometalation rate is higher for the iodido complex. The cyclometalated compounds exhibited good IC_50_ values, in the range of 1.72–5.82 μM, showing slightly better activities (for the same cell line, i.e. A549) than the ruthenium complexes with much lower cyclometalation rates, namely iodido compounds **1**_I_2__^*Me*^ and **2**_I_2__^*Me*^ (2.6 and 6.3 μM, respectively), as well as the previously reported compounds **1**_Cl_2__^*Me*^ and **2**_Cl_2__^*Me*^ (5.0 and 17.2 μM, respectively).^35^

All of the data obtained evidenced that steric hindrance provided by the phosphane ligand is the key with regard to cyclometalation. Therefore, future studies will be dedicated to the detailed investigation of the cyclometalation reaction, for instance using P(1-pyrenyl)R^2^R^3^ phosphane ligands bearing mixed R^2^ and R^3^ groups of various sizes (e.g., R^2^ = methyl and R^3^ = isopropyl).

## Experimental Section

### Materials and Methods

The ligands and ruthenium complexes were synthesized using standard Schlenk and vacuum-line techniques, under a purified dinitrogen atmosphere. All solvents were purified by using a solvent purification system or by applying standard procedures.^72^ DMSO containing 0.005% of water (Acros Organics, ref 34844) was used for the solvation studies. ^1^H, ^13^C{^1^H}, and ^31^P{^1^H}, and HSQC ^1^H–^13^C NMR spectra were recorded at 298 K in CDCl_3_ unless otherwise stated, using 400 MHz spectrometers. The chemical shifts (δ) are reported in parts per million (ppm) and are referenced to the nondeuterated solvent peak (usually CHCl_3_: 7.26 ppm for ^1^H spectra). IR spectra were recorded using a FT-IR spectrometer (in the range 4000–400 cm^–1^) equipped with an ATR unit, and the main absorption bands are reported in cm^–1^. High-resolution mass analyses (HRMS) were carried out at the Centres Cientfics i Tecnòlogics de la Universitat de Barcelona, with a time-of-flight instrument using electrospray ionization. Elemental analyses were carried out at the Centres Cientfics i Tecnòlogics de la Universitat de Barcelona; satisfactory elemental analyses were obtained for most of the organometallic compounds described. In the case of í**3**_dmso_^*iPr*^ the ^1^H NMR spectrum (Figure S43) revealed the presence of two molecules of uncoordinated dmso, and hence the analysis of the solvato complex is reported. It can be stressed here that the difficulty sometimes encountered in characterizing organometallic compounds with this technique has recently been commented in an Editorial Note of an ACS journal.^73^

### Preparation of Diisopropyl(1-pyrenyl)phosphane

#### Borane–diisopropyl(1-pyrenyl)phosphane Complex

1-Bromopyrene (1.12 g, 4.0 mmol) was dissolved in 20 mL of THF, and the resulting solution was cooled to −78 °C. A 1.6 M *n*-BuLi solution (2.4 mL, 3.8 mmol) was subsequently added using a syringe, and the mixture was stirred for 1 h. Chlorodiisopropylphosphane (0.51 mL, 534 mg, 3.5 mmol) was then added, and the reaction mixture was warmed to room temperature for 14 h. A 1 M borane·THF solution (7 mL, 7.0 mmol) was added, and the resulting solution was stirred for 1 h. Water (10 mL) was carefully added, and THF was removed under reduced pressure. The mixture was extracted with dichloromethane (3 × 10 mL), and the combined organic phases were washed with 20 mL of water. The final organic phase was dried over solid anhydrous sodium sulfate and filtered, and the solvent was removed under reduced pressure to give the crude product, which was purified by column chromatography (flash SiO_2_, hexane/ethyl acetate 95/5). The title product was obtained as a white solid. Yield: 419 mg (36%). ^1^H NMR (400 MHz, CDCl_3_): δ 8.86 (d, *J* = 9.2 Hz, 1H, Ar), 8.58 (dd, *J* = 11.2 Hz, 8.0 Hz, 1H, Ar), 8.29–8.17 (m, 5H, Ar), 8.11–8.05 (m, 2H, Ar), 2.94–2.85 (m, 2H, *i*-Pr), 1.40 (dd, ^3^*J*_HH_ + ^3^*J*_HP_ = 15.2 Hz, 7.2 Hz, 6H, *i*-Pr), 1.01 (dd, ^3^*J*_HH_ + ^3^*J*_HP_ = 14.8 Hz, 7.2 Hz, 6H, *i*-Pr) ppm (Figure S15). ^31^P{^1^H} NMR (162 MHz, CDCl_3_): δ +36.7 (s, br) ppm (Figure S16). HRMS (ESI): *m*/*z* calcd for C_22_H_30_BNP [M + NH_4_]^+^, 350.2203; found, 350.2207.

#### Diisopropyl(1-pyrenyl)phosphane (**L**)

The borane–diisopropyl(1-pyrenyl)phosphane complex (202 mg, 0.61 mmol) was dissolved in 20 mL of dichloromethane, and the resulting solution was cooled to 0 °C. HBF_4_·Et_2_O (0.43 mL, 3.1 mmol) was added, and the mixture was stirred for 1 h. A thoroughly deoxygenated solution of saturated aqueous NaHCO_3_ (10 mL) was carefully added to the mixture containing the formed phosphonium salt. The organic layer was then transferred to another flask, washed with thoroughly deoxygenated water, dried over sodium sulfate, filtered, and brought to dryness under reduced pressure. The title product was obtained as an air-sensitive solid. Yield: 181 mg (93%). ^1^H NMR (400 MHz, CDCl_3_): δ 9.19 (dd, *J* = 9.2 Hz, 6.0 Hz, 1H, Ar), 8.22–8.00 (m, 8H, Ar), 2.43 (s, br, 2H, *i*-Pr), 1.22 (dd, ^3^*J*_HH_ + ^3^*J*_HP_ = 15.6 Hz, 7.2 Hz, 6H, *i*-Pr), 0.98 (dd, ^3^*J*_HH_ + ^3^*J*_HP_ = 12.0 Hz, 6.8 Hz, 6H, *i*-Pr) ppm (Figure S17). ^31^P{^1^H} NMR (162 MHz, CDCl_3_): δ −8.8 (s) ppm (Figure S18).

### Preparation of Ruthenium Compounds

#### [RuCl_2_(η^6^-methyl benzoate)(diisopropyl(1-pyrenyl)phosphane)] (**1**_Cl_2__^*iPr*^)

Diisopropyl(1-pyrenyl)phosphane (180 mg, 0.57 mmol) was dissolved in 10 mL of dichloromethane, and [Ru(η^6^-methyl benzoate)Cl_2_]_2_ (184 mg, 0.30 mmol) was added. The resulting red solution was stirred for 1 h protected from light and filtered, and the solvent was removed under reduced pressure. The residue was recrystallized from dichloromethane/hexane to give the title product as a dark red solid. Yield: 250 mg (70%). IR: ν̅ 3056, 2960, 2927, 2872, 1732 (ν_C=O_), 1428, 1382, 1287, 1266, 1101, 1041, 850, 767, 735, 645, 605, 595 cm^–1^. ^1^H NMR (400 MHz, CDCl_3_): δ 8.85 (d, *J* = 9.6 Hz, 1H, Ar), 8.56 (t, *J* = 8.0 Hz, 1H, Ar), 8.33–8.28 (m, 4H, Ar), 8.22 (d, *J* = 8.8 Hz, 1H, Ar), 8.16–8.11 (m, 2H, Ar), 6.41 (s, br, 1H, *Ph*COOMe), 6.28 (s, br, 1H, *Ph*COOMe), 5.09 (s, br, 1H, *Ph*COOMe), 4.53 (s, br, 1H, *Ph*COOMe), 4.27 (s, br, 1H, *Ph*COOMe), 4.01 (s, 3H, PhCOO*Me*), 3.92 (s, br, 1H, *i*-Pr), 3.41 (s, br, 1H, *i*-Pr), 1.81 (d, *J* = 9.2 Hz, 3H, *i*-Pr), 1.74 (m, br 3H, *i*-Pr), 1.36 (d, *J* = 8.8 Hz, 6H, *i*-Pr), 0.66 (s, br, 3H, *i*-Pr) ppm (Figures S19 and S20). ^13^C{^1^H} NMR(101 MHz, CDCl_3_): δ 164.1 (C, Ph*C*OOMe), 133.2–123.4 (C, CH, Ar), 94.2 (s, 2CH, *Ph*COOMe), 91.1 (d, ^2^*J*_CP_ = 10.2 Hz, C, *Ph*COOMe), 87.9 (s, CH, *Ph*COOMe), 84.8 (s, br, CH, *Ph*COOMe), 80.9 (s, br, CH, *Ph*COOMe), 53.4 (s, br, CH_3_, PhCOO*Me*), 23.4 (s, CH, *i*-Pr), 23.2 (s, CH, *i*-Pr), 22.0 (s, CH_3_,*i*-Pr), 19.0 (s, 3CH_3_, *i*-Pr), 17.6 (s, 2CH_3_, *i*-Pr) ppm (Figures S20 and S21). ^31^P{^1^H} NMR (162 MHz, CDCl_3_): δ +38.9 (s) ppm (Figure S22). HRMS (ESI): *m*/*z* calcd for [M – 2Cl – H]^+^, 555.1021; found, 553.1025. Anal. Calcd for C_30_H_31_Cl_2_O_2_PRu: C 57.51; H 4.99. Found: C, 57.08; H 4.97. Single crystals of **1**_Cl_2__^*iPr*^ were obtained from CH_2_Cl_2_/*n*-hexane.

#### [RuI_2_(η^6^-methyl benzoate)(diisopropyl(1-pyrenyl)phosphane)] (**1**_I_2__^*iPr*^)

Compound **1**_I_2__^*iPr*^ was prepared from **1**_Cl_2__^*iPr*^ through halide exchange. Hence, a suspension of **1**_Cl_2__^*iPr*^ (470 mg, 0.75 mmol) and excess NaI (1.5 g, 10 mmol) in 40 mL of technical acetone was refluxed, protected from light. Complete halide substitution was achieved after 16 h of reflux. The solvent was removed under reduced pressure, and the crude solid obtained was extracted in CH_2_Cl_2_/water. After separation, the organic phase was dried with anhydrous Na_2_SO_4_ and filtered and dichloromethane was evaporated under reduced pressure. The powder obtained was recrystallized from dichloromethane/hexane, filtered, and washed with pentane. Compound **1**_I_2__^*iPr*^ was obtained as a brown crystalline solid with a yield of 72% (435 mg). Single crystals of **1**_I_2__^*iPr*^ could be obtained that were suitable for X-diffraction analysis. IR: ν̅ 3052, 2961, 2926, 2865, 1735 (ν_C=O_), 1430, 1296, 1265, 1104, 848, 761, 604 cm^–1^. ^1^H NMR (400 MHz, CDCl_3_): δ 9.03 (d, *J* = 9.2 Hz, 1H, Ar), 8.52 (t, *J* = 8.4 Hz, 1H, Ar), 8.34–8.20 (m, 5H, Ar), 8.15–8.10 (m, 2H, Ar), 6.49 (t, *J* = 5.6 Hz, 1H, *Ph*COOMe), 6.37 (d, *J* = 5.6 Hz, 1H, *Ph*COOMe), 5.24 (t, *J* = 5.6 Hz, 1H, *Ph*COOMe), 4.55 (dd, *J* = 9.6 Hz, *J* = 5.2 Hz, 1H, *Ph*COOMe), 4.39 (t, *J* = 5.2 Hz, 1H, *Ph*COOMe), 4.29–4.23 (m, 1H, *i*-Pr), 3.98 (s, 3H, PhCOO*Me*), 3.61–3.52 (m, 1H, *i*-Pr), 1.85 (dd, *J* = 16.4 Hz, *J* = 7.6 Hz, 3H, *i*-Pr), 1.71 (dd, *J* = 11.2 Hz, *J* = 7.2 Hz, 3H, *i*-Pr), 1.46 (dd, *J* = 16.0 Hz, *J* = 7.2 Hz, 3H, *i*-Pr), 0.66 (dd, *J* = 13.2 Hz, *J* = 7.2 Hz, 3H, *i*-Pr) ppm (Figures S23 and S24). ^13^C{^1^H} NMR (101 MHz, CDCl_3_): δ 164.1 (C, Ph*C*OOMe), 133.1–123.3 (C, CH, Ar), 93.8 (CH, *Ph*COOMe), 93.3 (CH, *Ph*COOMe), 90.5 (CH, *Ph*COOMe), 89.2 (d, *J* = 9.4 Hz, C, *Ph*COOMe), 87.1 (CH, *Ph*COOMe), 83.8 (CH, *Ph*COOMe), 53.3 (CH_3_, PhCOO*Me*), 30.5 (d, *J* = 25.7 Hz, CH, *i*-Pr), 29.6 (d, *J* = 25.3 Hz, CH, *i*-Pr), 23.4 (CH_3_, *i*-Pr), 20.3 (d, *J* = 7.0 Hz, CH_3_, *i*-Pr), 20.0 (d, *J* = 7.2 Hz, CH_3_, *i*-Pr), 18.4 (CH_3_, *i*-Pr) ppm (Figures S24 and S25). ^31^P{^1^H} NMR (162 MHz, CDCl_3_): δ +34.5 (s) ppm (Figure S26). HRMS (ESI): *m*/*z* calcd for [M – I]^+^, 683.0144; found, 683.0142. Anal. Calcd for C_30_H_31_I_2_O_2_PRu: C, 44.52; H, 3.86. Found: C, 43.26; H, 3.83. Single crystals of **1**_I_2__^*iPr*^ were obtained from CH_2_Cl_2_/*n*-hexane.

#### [RuCl_2_(η^6^-*p*-cymene)(diisopropyl(1-pyrenyl)phosphane)] (**2**_Cl_2__^*iPr*^)

Diisopropyl(1-pyrenyl)phosphane (180 mg, 0.57 mmol) was dissolved in 10 mL of dichloromethane, and [Ru(η^6^-*p*-cymene)Cl_2_]_2_ (139 mg, 0.23 mmol) was subsequently added. The resulting red solution was stirred for 1 h, protected from light, and the solvent was removed under reduced pressure. The residue was recrystallized from dichloromethane/hexane to give the title product as a dark red solid. Yield: 210 mg (74%). IR: ν̅ 3043, 2960, 2926, 2870, 1581, 1460, 1382, 1204, 1083, 1053, 1039, 852, 722, 645, 609, 508 cm^–1^. ^1^H NMR (400 MHz, CDCl_3_): δ 8.85 (d, *J* = 9.2 Hz, 1H, Ar), 8.56 (t, *J* = 8.0 Hz, 1H, Ar), 8.31–8.25 (m, 3H, Ar), 8.22 (d, *J* = 5.6 Hz, 1H, Ar), 8.19–8.08 (m, 3H, Ar), 5.49 (s, br, 1H, *p*-cym), 4.97 (s, br, 1H, *p*-cym), 4.67 (s, br, 1H, *p*-cym), 4.17 (s, br, 1H, *p*-cym), 3.92 (s, br, 1H, *i*-Pr), 3.42 (s, br, 1H, *i*-Pr), 3.02 (h, ^3^*J*_HH_ = 6.8 Hz, 1H, *p*-cym), 1.83 (s, br, 3H, *i*-Pr), 1.68 (s, br, 3H, *i*-Pr), 1.43 (s, 3H, *p*-cym), 1.32 (s, br, 6H, *i*-Pr), 1.17 (s, br, 3H, *i*-Pr), 0.66 (s, br, 3H, *i*-Pr) ppm (Figures S27 and S28). ^13^C{^1^H} NMR (101 MHz, CDCl_3_): δ 133.6–123.2 (C, CH, Ar), 111.6 (d, ^2^*J*_CP_ = 5.2 Hz, C, Ar), 98.2 (s, C, *p*-cym), 88.6 (s, br, CH, *p*-cym), 87.8 (s, br, CH, *p*-cym), 86.6 (s, br, CH, *p*-cym), 85.1 (s, br, CH, *p*-cym), 30.5 (s, 2CH, *i*-Pr), 23.5–18.1 (m, 2CH, 4CH_3_, *i*-Pr), 17.7 (s, CH_3_, *p*-cym) ppm (Figures S28 and S29). ^31^P{^1^H} NMR (162 MHz, CDCl_3_): δ +36.3 (s) ppm (Figure S30). HRMS (ESI): *m*/*z* calcd for [M – 2Cl – H]^+^, 553.1592; found, 553.1593. Anal. Calcd for C_32_H_37_Cl_2_PRu: C, 61.54; H, 5.97. Found: C, 61.76; H, 6.11. Single crystals of **2**_Cl_2__^*iPr*^ were obtained from CH_2_Cl_2_/*n*-hexane.

#### [RuI_2_(η^6^-*p*-cymene)(diisopropyl(1-pyrenyl)phosphane)] (**2**_I_2__^*iPr*^)

Compound **2**_I_2__^*iPr*^ was obtained from **2**_Cl_2__^*iPr*^, by applying the procedure described above for the preparation of **1**_I_2__^*iPr*^. With 423 mg (0.68 mmol) of **2**_CI_2__^*iPr*^ as the starting material and after 4 h of reflux, compound **2**_I_2__^*iPr*^ was obtained as a brown crystalline solid with a yield of 75% (410 mg). Single crystals of **2**_I_2__^*iPr*^ could be obtained that were suitable for X-ray diffraction analysis. IR: ν̅ 2952, 2926, 2870, 1448, 1200, 1026, 848, 609, 509 cm^–1^. ^1^H NMR (400 MHz, CDCl_3_): δ 8.96 (d, *J* = 9.6 Hz, 1H, Ar), 8.51 (t, *J* = 8.4 Hz, 1H, Ar), 8.32–8.23 (m, 4H, Ar), 8.20 (d, *J* = 8.8 Hz, 1H, Ar), 8.13 (d, *J* = 10.0 Hz, 1H, Ar), 8.09 (d, *J* = 7.6 Hz, 1H, Ar), 5.50 (s, br, 1H, *p*-cym), 4.86 (s, br, 1H, *p*-cym), 4.70 (s, br, 1H, *p*-cym), 4.40 (s, br, 1H, *p*-cym), 4.27 (s, br, 1H, *i*-Pr), 3.53 (s, br, 1H, *i*-Pr), 3.34 (sept, *J* = 6.8 Hz, 1H, *p*-cym), 1.85 (s, br, 3H, *i*-Pr), 1.69 (s, 3H, *p*-cym), 1.43 (s, br, 3H, *i*-Pr), 0.96 (s, br, 3H, *i*-Pr), 0.63 (s, br, 3H, *i*-Pr) ppm (Figures S31 and S32). ^13^C{^1^H} NMR (101 MHz, CDCl_3_): δ 133.9–122.8 (C, CH, Ar), 110.0 (C, *p*-cym), 101.3 (C, *p*-cym), 87.6–86.4 (4CH, *p*-cym), 31.4 (CH, *i*-Pr), 23.9 (br, 2CH_3_, *i*-Pr), 22.2 (br, CH_3_, *i*-Pr), 20.4 (br, CH_3_, *i*-Pr), 20.0 (br, CH_3_, *i*-Pr), 19.0 (CH_3_, *i*-Pr), 18.9 (br, CH_3_, *p*-cym) ppm (Figures S32 and S33). ^31^P{^1^H} NMR (162 MHz, CDCl_3_): δ +31.3 (s) ppm (Figure S34). HRMS (ESI): *m*/*z* calcd for [M – I]^+^, 681.0715; found, 681.0720. Anal. Calcd for C_32_H_37_I_2_PRu: C, 47.60; H, 4.62. Found: C, 46.44; H, 4.69. Single crystals of **2**_I_2__^*iPr*^ were obtained from CH_2_Cl_2_/*n*-hexane.

#### [RuCl(η^6^-*p*-cymene)(κ^2^*C*-diisopropyl(1-pyrenyl)phosphane)] (**3**_Cl_^*iPr*^)

A suspension of [RuCl(μ-Cl)(η^6^-*p*-cymene)]_2_ (643 mg, 1.05 mmol), ligand **L** (716 mg, 2.25 mmol), and NaOAc (492 mg, 5.94 mmol) in 160 mL of methanol was stirred for 4 h at room temperature, protected from light. The solvent was removed under reduced pressure, and the residue was extracted with dichloromethane/water. The combined organic phase was dried with anhydrous Na_2_SO_4_ and filtered. After removal of the solvent, the crude product was purified by column chromatography (SiO_2_, dichloromethane/ethyl acetate 99.5/0.5). The solvent was removed under reduced pressure to give the title product as an orange solid. Yield: 646 mg (52%). IR: ν̅ 3029, 2954, 2923, 2867, 1667, 1437, 1384, 1301, 1246, 1184, 1111, 1032, 836, 739, 615 cm^–1^. ^1^H NMR (CDCl_3_, 400 MHz): δ 8.93 (s, 1H, Ar), 8.11–7.93 (m, 7H, Ar), 6.23 (d, *J* = 4.8 Hz, 1H, *p*-cym), 6.22 (d, *J* = 5.2 Hz, 1H, *p*-cym), 5.33 (d, *J* = 6.4 Hz, 1H, *p*-cym), 4.94 (d, *J* = 6.0 Hz, 1H, *p*-cym), 3.05–2.94 (m, 1H, *i*-Pr), 2.94–2.77 (m, 2H, *i*-Pr), 1.97 (s, 3H, *p*-cym), 1.59 (dd, *J* = 14.4 Hz, 7.2 Hz, 3H, *i*-Pr), 1.43 (dd, *J* = 15.6 Hz, 7.2 Hz, 3H, *i*-Pr), 1.23 (d, *J* = 6.8 Hz, 3H, *i*-Pr), 1.12 (d, *J* = 6.8 Hz, 3H, *i*-Pr), 1.10 (dd, *J* = 14.4 Hz, 7.2 Hz, 3H, *i*-Pr), 1.06 (dd, *J* = 13.6 Hz, 6.8 Hz, 3H, *i*-Pr) ppm (Figures S35 and S36). ^13^C{^1^H} NMR (CDCl_3_, 101 MHz): δ 169.2–122.4 (C, CH, Ar), 110.9 (C, *p*-cym), 97.6 (d, *J*_CP_ = 6.1 Hz, CH, *p*-cym), 96.0 (C, *p*-cym), 92.0 (CH, *p*-cym), 88.6 (CH, *p*-cym), 84.9 (CH, *p*-cym), 30.8 (CH, *i*-Pr), 28.6 (d, *J*_CP_ = 25.1 Hz, CH, *i*-Pr), 26.7 (d, *J*_CP_ = 24.9 Hz, CH, *i*-Pr), 23.2 (CH_3_, *i*-Pr), 22.6 (CH_3_, *i*-Pr), 21.4 (d, *J*_CP_ = 1.8, CH_3_, *i*-Pr), 19.7 (d, *J*_CP_ = 2.5 Hz, CH_3_, *i*-Pr), 19.21 (CH_3_, *i*-Pr), 19.16 (CH_3_, *i*-Pr), 18.3 (CH_3_, *p*-cym) ppm (Figures S36 and S37). ^31^P{^1^H} NMR (CDCl_3_, 162 MHz): δ +80.8 (s) ppm (Figure S38). HRMS (ESI): *m*/*z* calcd for [M]^+^, 588.1281; found, 588.1305; calcd for [M – Cl]^+^, 533.1592; found, 533.1600. Anal. Calcd for C_32_H_36_ClPRu: C, 65.35; H, 6.17. Found: C, 65.35; H, 6.24. Single crystals of **3**_Cl_^*iPr*^ were obtained from CH_2_Cl_2_/*n*-hexane.

#### [RuI(η^6^-*p*-cymene)(κ^2^*C*-diisopropyl(1-pyrenyl)phosphane)] (**3**_I_^*iPr*^)

A suspension of **3**_Cl_^*iPr*^ (140 mg, 0.24 mmol) and NaI (893 mg, 6.00 mmol) in 20 mL of technical acetone was refluxed for 4 h, protected from light. The solvent was removed under reduced pressure, and the residue was extracted with dichloromethane/water. The combined organic phase was dried with anhydrous Na_2_SO_4_ and filtered. After evaporation of the solvent, the title product was obtained as a brown solid. Yield 133 mg (82%). IR: ν̅ 3033, 2960, 2921, 2866, 1707, 1568, 1436, 1382, 1259, 1079, 1017, 795, 737, 687, 653, 605 cm^–1^. ^1^H NMR (CDCl_3_, 400 MHz): δ 8.76 (s, 1H, Ar), 8.09–7.92 (m, 7H, Ar), 6.04 (d, *J* = 6.0 Hz, 2H, *p*-cym), 5.59 (d, *J* = 6.8 Hz, 1H, *p*-cym), 5.17 (d, *J* = 6.0 Hz, 1H, *p*-cym), 3.23–3.12 (m, 1H, *i*-Pr), 3.05 (sept, *J* = 6.8 Hz, 1H, *i*-Pr), 2.83–2.70 (m, 1H, *i*-Pr), 2.10 (s, 3H, *p*-cym), 1.64 (dd, *J* = 14.4 Hz, 7.2 Hz, 3H, *i*-Pr), 1.53 (dd, *J* = 16.0 Hz, 7.6 Hz, 3H, *i*-Pr), 1.29 (d, *J* = 6.8 Hz, 3H, *i*-Pr), 1.11 (d, *J* = 6.8 Hz, 3H, *i*-Pr), 1.01 (dd, *J* = 14.8 Hz, 7.2 Hz, 3H, *i*-Pr), 0.93 (dd, *J* = 13.2 Hz, 6.8 Hz, 3H, *i*-Pr) ppm (Figures S39 and S40). ^13^C{^1^H} NMR (CDCl_3_, 101 MHz): δ 165.1–122.3 (C, CH, Ar), 112.1 (C, *p*-cym), 98.0 (C, *p*-cym), 96.3 (d, *J*_CP_ = 5.5 Hz, CH, *p*-cym), 90.0 (d, *J*_CP_ = 3.5 Hz, CH, *p*-cym), 88.7 (CH, *p*-cym), 86.3 (CH, *p*-cym), 31.3 (CH, *i*-Pr), 30.2 (d, *J*_CP_ = 26.4 Hz, CH, *i*-Pr), 29.8 (d, *J*_CP_ = 24.5 Hz, CH, *i*-Pr), 23.7 (CH_3_, *i*-Pr), 23.1 (d, *J*_CP_ = 2.2 Hz, CH_3_, *i*-Pr), 22.6 (s, CH_3_, *i*-Pr), 19.8 (CH_3_, *i*-Pr), 19.7 (d, *J*_CP_ = 2.6 Hz, CH_3_, *i*-Pr), 19.0 (CH_3_, *i*-Pr), 18.7 (CH_3_, *p*-cym) ppm (Figures S40 and S41). ^31^P{^1^H} NMR (CDCl_3_, 162 MHz): δ +79.3 (s) ppm (Figure S42). HRMS (ESI): *m*/*z* calcd for [M + H]^+^, 681.0721; found, 681.0728; calcd for [M – I]^+^, 553.1592; found, 553.1593. Anal. Calcd for C_32_H_36_IPRu: C, 56.56; H, 5.34. Found: C 55.44; H 5.78. Single crystals of **3**_I_^*iPr*^ were obtained from CH_2_Cl_2_/*n*-hexane.

#### [Ru(η^6^-*p*-cymene)(κ*S*-dmso)(κ^2^*C*-diisopropyl(1-pyrenyl)phosphane)]PF_6_ (**3**_dmso_^*iPr*^)

Complex **3**_Cl_^*iPr*^ (300 mg, 0.51 mmol) was dissolved in dichloromethane (30 mL) and dmso (0.35 mL, 4.93 mmol). Thallium hexafluorophosphate (196 mg, 0.54 mmol) was added, and the mixture was stirred overnight, protected from light. The suspension was filtered, and the solvent was removed under reduced pressure. The crude product was recrystallized in dichloromethane/diethyl ether at −20 °C, yielding the title product as a fine yellow solid. Yield 375 mg (95%). IR: ν̅ 2963, 1440, 1384, 1293, 1242, 1106, 1013, 832, 740, 668, 597, 574 cm^–1^. ^1^H NMR (acetone-*d*_6_, 400 MHz): δ 8.88 (s, 1H, Ar), 8.43–8.35 (m, 2H, Ar), 8.31–8.23 (m, 4H, Ar), 8.09 (t, *J* = 7.06 Hz, 1H, Ar), 7.11 (d, *J* = 6.4 Hz, 1H, *p*-cym), 7.08 (d, *J* = 6.8 Hz, 1H, *p*-cym), 6.35 (d, *J* = 5.6 Hz, 1H, *p*-cym), 6.10 (d, *J* = 6.4 Hz, 1H, *p*-cym), 3.56–3.43 (m, 1H, *i*-Pr), 3.45 (s, 3H, dmso), 3.29 (sept, *J* = 6.8 Hz, 1H, *i*-Pr), 2.90–2.81 (m, 1H, *i*-Pr), 2.46 (s, 3H, *p*-cym), 1.76 (dd, *J* = 16.8 Hz, 7.2 Hz, 3H, *i*-Pr), 1.66 (s, 3H, dmso), 1.53 (dd, *J* = 17.2 Hz, 7.2 Hz, 3H, *i*-Pr), 1.45 (d, *J* = 6.8 Hz, 3H, *i*-Pr), 1.38 (dd, *J* = 13.2 Hz, 6.8 Hz, 3H, *i*-Pr), 1.18 (d, *J* = 6.8 Hz, 3H, *i*-Pr), 0.29 (dd, *J* = 16.4 Hz, 6.8 Hz, 3H, *i*-Pr) ppm (Figures S43 and S44). ^13^C{^1^H} NMR (CD_3_COCD_3_, 101 MHz): δ 160.3–123.4 (C, CH, Ar), 114.4 (C, *p*-cym), 98.6 (CH, *p*-cym), 97.3 (br, CH, *p*-cym), 95.5 (CH, *p*-cym), 93.9 (br, CH, *p*-cym), 53.7 (CH_3_, dmso), 47.2 (CH_3_, dmso), 32.1 (CH, *i*-Pr), 31.2 (d, *J*_CP_ = 27.7 Hz, CH, *i*-Pr), 26.4 (d, *J*_CP_ = 27.0 Hz, CH, *i*-Pr), 24.8 (CH_3_, *i*-Pr), 21.4 (CH_3_, *i*-Pr), 19.6 (CH_3_, *i*-Pr), 19.4 (CH_3_, *i*-Pr), 19.2 (d, *J*_CP_ = 2.4, CH_3_, *i*-Pr), 19.0 (CH_3_, *p*-cym), 18.1 (d, *J*_CP_ = 6.1, CH_3_, *i*-Pr) ppm (Figures S44 and S45). ^31^P{^1^H} NMR (CD_3_COCD_3_, 162 MHz): δ +86.6 (s), −144.2 (sept, *J*_PF_ = 708.8 Hz) ppm (Figure S46). ^19^F{^1^H} NMR (CD_3_COCD_3_, 377 MHz): δ −72.5 (d, *J*_FP_ = 708.8 Hz) ppm (Figure S47). HRMS (ESI): *m*/*z* calcd for [M – PF_6_]^+^, 631.1732; found, 631.1741; calcd for [M – PF_6_ – dmso]^+^, 553.1592, found, 553.1604. Anal. Calcd for C_38_H_54_F_6_O_3_P_2_RuS_3_ (**3**_dmso_^*iPr*^·2dmso): C, 48.97; H, 5.84. Found: C 48.89; H, 5.71. The presence of two molecules of dmso is observed as well in the ^1^H NMR spectrum of this compound (see Figure S43). Single crystals of **3**_dmso_^*iPr*^ were obtained from CH_2_Cl_2_/*n*-hexane.

#### [RuI_2_(η^6^-methyl benzoate)(dimethyl(1-pyrenyl)phosphane)] (**1**_I_2__^*Me*^)

Compound **1**_I_2__^*Me*^ was prepared from [RuCl_2_(η^6^-methyl benzoate)(dimethyl(1-pyrenyl)phosphane)] (**1**_Cl_2__^*Me*^), which was described previously.^35^ With 240 mg (0.42 mmol) of the chloro complex as the starting material, iodo complex **1**_I_2__^*Me*^ was obtained as a brown crystalline solid with a yield of 37% (116 mg), after 11 days of reflux in technical acetone. The conversion of **1**_I_2__^*Me*^ into **1**_I_2__^*Me*^ could be followed by ^31^P NMR (see Figure S48). Single crystals of **1**_I_2__^*Me*^ could be obtained that were suitable for X-ray diffraction analysis. IR: ν̅ 3065, 3035, 1935, 1733 (ν_C=O_), 1288, 1270, 913, 849 cm^–1^. ^1^H NMR (400 MHz, CDCl_3_): δ 9.31 (d, *J* = 9.6 Hz, 1H, Ar), 8.51–8.10 (m, 8H, Ar), 6.25 (s, br, 2H, *Ph*COOMe), 5.70 (t, *J* = 5.6 Hz, 1H, *Ph*COOMe), 4.91 (s, br, 2H, *Ph*COOMe), 3.90 (s, 3H, PhCOO*Me*), 2.49 (d, *J* = 9.6 Hz, 6H, *i*-Pr) ppm (Figure S49). ^13^C{^1^H} NMR (101 MHz, CDCl_3_): δ 165.4 (C, Ph*C*OOMe), 130.3–123.6 (C, CH, Ar), 95.0 (CH, *Ph*COOMe), 53.2 (CH_3_, PhCOO*Me*), 20.4 (CH_3_, *i*-Pr) ppm (Figure S50). ^31^P{^1^H} NMR (162 MHz, CDCl_3_): δ −5.2 (s) ppm (Figure S51). HRMS (ESI): *m*/*z* calcd for [M – I]^+^, 626.9518; found, 626.9518. Anal. Calcd for C_26_H_23_I_2_O_2_PRu: C, 41.45; H, 3.08. Found: C, 41.25; H, 3.11.

#### [RuI_2_(η^6^-*p*-cymene)(dimethyl(1-pyrenyl)phosphane)] (**2**_I_2__^*Me*^)

Compound **2**_I_2__^*Me*^ was prepared from [RuCl_2_(η^6^-*p*-cymene)(dimethyl(1-pyrenyl)phosphane)] (**2**_Cl_2__^*Me*^), which was described previously.^35^ Using 230 mg (0.40 mmol) of the chloro precursor, compound **2**_I_2__^*Me*^ was obtained with a yield of 68% (206 mg), after 24 h of reflux in technical acetone. Single crystals of **2**_I_2__^*Me*^ could be obtained that were suitable for X-ray diffraction analysis. IR: ν̅ 2957, 2917, 1624, 1430, 1383, 913, 845, 719, 698, 600, 532 cm^–1^. ^1^H NMR (101 MHz, CDCl_3_): δ 9.32 (d, *J* = 9.6 Hz, 1H, Ar), 8.39–8.26 (m, 5H, Ar), 8.21 (d, *J* = 8.0 Hz, 1H, Ar), 8.14 (d, *J* = 9.2 Hz, 1H, Ar), 8.11 (t, *J* = 8.0 Hz, 1H, Ar), 5.06 (d, *J* = 6.0 Hz, 2H, *p*-cym), 4.78 (s, br, 2H, *p*-cym), 3.14 (sept, *J* = 6.8 Hz, 1H, *p*-cym), 2.46 (d, *J* = 9.2 Hz, 6H, P*Me*), 1.98 (s, 3H, *p*-cym), 1.06 (d, *J* = 7.2 Hz, 6H, *p*-cym) ppm (Figures S52 and S53). ^13^C{^1^H} NMR (101 MHz, CDCl_3_): δ 133.3–121.1 (C, CH, Ar), 108.1 (C, *p*-cym) 96.2 (C, *p*-cym), 91.3 (d, *J* = 15.6 Hz, 2CH, *p*-cym), 86.2 (br, 2CH, *p*-cym), 31.9 (CH, *p*-cym), 22.6 (br, 2CH_3_, P*Me*), 20.0 (CH_3_, *p*-cym) ppm (Figures S53 and S54). ^31^P{^1^H} NMR (162 MHz, CDCl_3_): δ −5.8 (s) ppm (Figure S55). HRMS (ESI): *m*/*z* calcd for [M – I]^+^, 625.0089; found, 625.0090. Anal. Calcd for C_28_H_29_I_2_PRu: C, 44.76; H 3.89. Found: C, 44.78; H, 3.98.

### X-ray Crystallography

Data for compounds **1**_Cl_2__^*iPr*^, **1**_I_2__^*iPr*^, **2**_I_2__^*iPr*^, **3**_Cl_^*iPr*^, **3**_I_^*iPr*^, **3**_dmso_^*iPr*^, **1**_I_2__^*Me*^, and **2**_I_2__^*Me*^ (see the Supporting Information) were collected on a Bruker APEX II QUAZAR diffractometer equipped with a microfocus multilayer monochromator with Mo Kα radiation (λ = 0.71073 Å). Data for compound **2**_Cl_2__^*iPr*^ were collected using a Bruker D8 diffractometer with a Photon 100 detector on the Advanced Light Source beamline 11.3.1 at Lawrence Berkeley National Laboratory, from a silicon 111 monochromator (λ = 0.7749 Å). Data reduction and absorption corrections were performed by using SAINT and SADABS, respectively.^74^ The structures were solved using SHELXT^75^ and refined with full-matrix least squares on *F*^2^ by using SHELXL.^76^ Low-quality data for compound **3**_dmso_^*iPr*^ could not be improved due to small crystal dimensions (very thin plates of 80 μm). In this case, a void containing only diffuse electron density was analyzed and taken into account with Olex2/Solvent Mask.^77^ An estimated content of five diffuse lattice CH_2_Cl_2_ molecules per asymmetric unit cell was deduced and included in the formula. All details can be found in CCDC 2054649–2054657, which contain the supplementary crystallographic data for the present paper. These data can be obtained free of charge from The Cambridge Crystallographic Data Center via https://summary.ccdc.cam.ac.uk/structure.summary.form.

### Computational Details

All calculations were performed using the Gaussian 09 (revision D01)^78^ electronic structure package using a 10^–8^ convergence criterion for the density matrix elements. The PBE functional was used for both exchange and correlation functionals.^79,80^ The fully optimized contracted triple-ζ all-electron Gaussian basis set with added polarization functions developed by Ahlrichs and co-workers was used for all the elements in all molecules,^81^ except for the ruthenium and iodine atoms, for which the Stuttgart/Dresden effective core potential (SDD)^82−84^ basis set was used. Full system optimization was carried out for all compounds investigated, followed by the corresponding vibrational analysis to calculate the thermochemical properties. The dimethyl sulfoxide solvent properties were modeled using a polarizable continuum model (self-consistent reaction field approximation) with Truhlar’s SMD variation.^85^

### Cell Culture and Viability Assays

Human lung adenocarcinoma A549, colorectal adenocarcinoma SW620, and breast adenocarcinoma MCF7 cell lines were obtained from the American Type Culture Collection (ATCC, Manassas, VA, USA). A549 and SW620 cells were cultured in DMEM medium with 10% heat-inactivated fetal bovine serum (FBS; Life Technologies, Carlsbad, CA, USA), 100 U mL^–1^ penicillin, 100 μg mL^–1^ streptomycin, and 2 mM glutamine. The MCF-7 cell line was cultured in DMEM–F12 (HAM) media (1/1) with 10% FBS, 50 μM sodium pyruvate, 10 μg mL^–1^ insulin (Sigma-Aldrich Chemical Co., St. Louis, MO, USA), 100 U mL^–1^ penicillin, 100 μg mL^–1^ streptomycin, and 2 mM glutamine. All reagents not specified above were obtained from Biological Industries, Beit Haemek, Israel. Cells were grown at 37 °C under a 5% CO_2_ atmosphere.

Cell viability assays were conducted using the MTT (3-(4,5-dimethylthiazol-2-yl)-2,5-diphenyltetrazolium bromide) assay. Amounts of 10^4^ cells were seeded in 96-well plates (10^5^ cells mL^–1^) and allowed to grow for 24 h. For single-point experiments, the cells were treated with compounds **1**_Cl_2__^*iPr*^, **1**_I_2__^*iPr*^, **2**_Cl_2__^*iPr*^, and **2**_I_2__^*iPr*^ at 10 μM for 24 h. For dose–response assays, the cells were treated with different concentrations of compounds **2**_Cl_2__^*iPr*^, **2**_I_2__^*iPr*^, **1**_I_2__^*Me*^, and **2**_I_2__^*Me*^ (from 0.8 to 100 μM), and **3**_Cl_^*iPr*^, **3**_I_^*iPr*^, and **3**_dmso_^*iPr*^ (from 0.4 to 50 μM) for 24 h, using DMSO complex solutions of different “aging times”, Day 0 corresponds to a freshly prepared solution and days 1, 2, 5, and 7 correspond to 1-, 2-, 5- and 7-day-old solution, respectively. After the 24 h treatment, a 10 μM solution of MTT was added to each well and the plates were incubated for an additional 2 h at 37 °C. The medium was removed, and the purple formazan crystals were dissolved in 100 μL of DMSO. The absorbance was measured at 570 nm using a multiwell plate reader (Multiskan FC, Thermo Scientific). The cell viability was calculated according to the relation viability (%) = [(absorbance of treated wells)/(absorbance of control wells)] × 100. The IC_50_ values (corresponding to the compound concentrations that produce 50% reduction in cell viability) were obtained from the dose–response curves using GraphPad Prism V5.0 for Windows (GraphPad Software, San Diego, CA, USA). All data are shown as the mean value ± SD of at least three independent experiments for single-point assays and for the dose–response curves.

## Supporting Information Available

Synthetic procedures for **L** and **L=O**, X-ray crystallographic data, ^1^H, ^13^C{^1^H}, ^31^P{^1^H}, and ^1^H–^13^C HSQC NMR spectra, time-dependent NMR studies, schematic representation of the DMSO-mediated cyclometalation reaction, rate constants for the solvation of **3**_Cl_^*iPr*^, and Cartesian coordinates (computational calculations) (PDF)

## Accession Codes

CCDC 2054649–2054657 contain the supplementary crystallographic data for this paper. These data can be obtained free of charge via www.ccdc.cam.ac.uk/data_request/cif, or by emailing data_request@ccdc.cam.ac.uk, or by contacting The Cambridge Crystallographic Data Centre, 12 Union Road, Cambridge CB2 1EZ, UK; fax: +44 1223 336033.
