# Supplementary material for: Piano-Stool Ruthenium(II) Complexes with Delayed Cytotoxic Activity: Origin of the Lag Time
Source: Inorg Chem. 2021 May 12;60(11):7974–90. doi: 10.1021/acs.inorgchem.1c00507 (PMC8659375; doi:10.1021/acs.inorgchem.1c00507)
Supplement: Supplementary file 1 — ic1c00507_si_001.pdf [file ic1c00507_si_001.pdf]

Supporting Information

Piano-Stool Ruthenium(II) Complexes with Delayed Cytotoxic Activity: Origin of the Lag Time

Laia Rafols,[†] *Dana Josa,*[†‖] *David Aguilà,*[†‖] *Leoní A. Barrios,*[†‖] *Olivier Roubeau,*[‡] *Jordi Cirera,*[†▽] *Vanessa Soto-Cerrato,*[§⊥] *Ricardo Pérez-Tomás,*[§⊥] *Manuel Martínez,*[†‖] *Arnald Grabulosa**[†‖] *and Patrick Gamez **[†‖#].

† Departament de Química Inorgànica i Orgànica, Facultat de Química, Secció de Química Inorgànica, Universitat de Barcelona, Martí i Franquès, 1-11, 08028 Barcelona, Spain. E-mail: arnald.grabulosa@qi.ub.es, patrick.gamez@qi.ub.es

‖ Institute of Nanoscience and Nanotechnology (IN^{2}UB), Universitat de Barcelona, 08028 Barcelona, Spain

‡ Instituto de Ciencia de Materiales de Aragón (ICMA), CSIC and Universidad de Zaragoza, Pedro Cerbuna 12, 50009 Zaragoza, Spain

▽ Institut de Recerca de Química Teòrica i Computacional, Universitat de Barcelona, 08028 Barcelona, Spain

§ Department of Pathology and Experimental Therapeutics, Faculty of Medicine and Health Sciences, University of Barcelona, Campus Bellvitge, Feixa LLarga s/n, 08907 L'Hospitalet de Llobregat (Barcelona), Spain

⊥ Oncobell Program, Institut d'Investigació Biomèdica de Bellvitge (IDIBELL), 08907 L'Hospitalet de Llobregat, Barcelona, Spain

Catalan Institution for Research and Advanced Studies, Passeig Lluís Companys 23, 08010 Barcelona, Spain

Table of Contents

**Scheme S1.** Synthetic pathway for the preparation of diisopropyl(1-pyrenyl)-phosphane (**L**).	S4
**Table S1**. Crystallographic data for compounds $\mathbf{1}_{\mathrm{Cl_2}}^{iPr}$ and $\mathbf{1}_{I_2}^{iPr}$.	S5
**Table S2**. Crystallographic data for compounds $\mathbf{2}_{\mathrm{Cl_2}}^{iPr}$ and $\mathbf{2}_{\mathrm{I_2}}^{iPr}$.	S6
**Figure S1.** Schematic representation of the crystal structures of $\mathbf{1}_{\mathrm{Cl_2}}^{iPr}$, $\mathbf{1}_{\mathrm{I_2}}^{iPr}$, $\mathbf{2}_{\mathrm{Cl_2}}^{iPr}$ and $\mathbf{2}_{\mathrm{I_2}}^{iPr}$.	S7
**Table S3.** Selected bond distances (Å) and angles (°) for compounds $\mathbf{1}_{\mathrm{Cl_2}}^{iPr}$, $\mathbf{1}_{\mathrm{I_2}}^{iPr}$, $\mathbf{2}_{\mathrm{Cl_2}}^{iPr}$ and $\mathbf{2}_{\mathrm{I_2}}^{iPr}$.	S7
Synthetic procedure for the preparation of diisopropyl(1-pyrenyl)phosphane oxide.	S8
**Figure S2.** ^{1}H NMR spectrum of diisopropyl(1-pyrenyl)phosphane oxide.	S8
**Figure S3.** Representation of the crystal structure of diisopropyl(1-pyrenyl)phosphane oxide.	S9
**Figure S4.** ^{31}P{^{1}H} NMR spectrum of diisopropyl(1-pyrenyl)phosphane oxide.	S9

Table S4. Crystallographic data for diisopropyl(1-pyrenyl)phosphane oxide. S10
Figure S5. Time-dependent $^{31}P\{^1H\}$ NMR spectra of compound $\mathbf{2}_{Cl_2}^{iPr}$ in DMSO-d$_6$ S11
Figure S6. Time-dependent $^{31}P\{^1H\}$ NMR spectra of compound $\mathbf{2}_{Cl_2}^{iPr}$ in $CDCl_3$ S11
Figure S7. Time-dependent $^{31}P\{^1H\}$ NMR spectra of compound $\mathbf{2}_{I_2}^{iPr}$ in $CDCl_3$ S12
Figure S8. Time-dependent $^{31}P\{^1H\}$ NMR spectra of compound $\mathbf{1}_{Cl_2}^{iPr}$ in DMSO-d$_6$ S12
Figure S9. Time-dependent $^{31}P\{^1H\}$ NMR spectra of compound $\mathbf{1}_{I_2}^{iPr}$ in DMSO-d$_6$ S13
Figure S10. Time evolution of the $^{31}P\{^1H\}$ chemical shift of [Ru(η^6-*p*-cymene)(k^2C-diisopropyl(1-pyrenyl)phosphane)(dmso)]$^+$ for a 48 h-aged solution of complex $\mathbf{2}_{I_2}^{iPr}$ (spectra recorded in DMSO-d^6:D_2O 25:75). S13
Table S5. Crystallographic data for compounds $\mathbf{3}_{Cl}^{iPr}$ and $\mathbf{3}_{I}^{iPr}$. S14
Table S6. Selected bond distances (Å) and angles (°) for compounds $\mathbf{3}_{Cl}^{iPr}$ and $\mathbf{3}_{I}^{iPr}$. S15
Table S7. Crystallographic data for compound $\mathbf{3}_{dmso}^{iPr}$. S16
Table S8. Selected bond distances (Å) and angles (°) for compound $\mathbf{3}_{dmso}^{iPr}$. S17
Figure S11. Proposed mechanism for the DMSO-mediated (O-donor Lewis base) cyclometallation reaction. S18
Table S9. Observed rate constants for the solvation of compound $\mathbf{3}_{Cl}^{iPr}$ in various solvents as a function of the temperature and pressure. S18
Table S10. Observed rate constants for the solvation of compounds $\mathbf{3}_{I}^{iPr}$ and $\mathbf{2}_{C(dmso)}^{iPr}$ in various solvents as a function of the temperature and pressure. S19
Table S11. Crystallographic data for compounds $\mathbf{1}_{I_2}^{Me}$ and $\mathbf{2}_{I_2}^{Me}$. S20
Scheme S2. Schematic representation of complexes $\mathbf{1}_{I_2}^{Me}$ and $\mathbf{2}_{I_2}^{Me}$. S20
Figure S12. Representation of the crystal structures of compounds $\mathbf{1}_{I_2}^{Me}$ and $\mathbf{2}_{I_2}^{Me}$. S21
Table S12. Selected bond distances (Å) and angles (°) for compounds $\mathbf{1}_{I_2}^{Me}$ and $\mathbf{2}_{I_2}^{Me}$. S21
Figure S13. Time-dependent $^{31}P\{^1H\}$ NMR spectra of compound $\mathbf{1}_{I_2}^{Me}$ in DMSO-d^6. S22
Figure S14. Time-dependent $^{31}P\{^1H\}$ NMR spectra of compound $\mathbf{2}_{I_2}^{Me}$ in DMSO-d^6. S22
Figure S15. ^1H NMR spectrum of **L·BH₃**. S23
Figure S16. $^{31}P\{^1H\}$ NMR spectrum of **L·BH₃**. S23
Figure S17. ^1H NMR spectrum of **L**. S24
Figure S18. $^{31}P\{^1H\}$ NMR spectrum of **L**. S24
Figure S19. ^1H NMR spectrum of $\mathbf{1}_{Cl_2}^{iPr}$. S25
Figure S20. ^1H-^{13}C HSQC NMR spectrum of $\mathbf{1}_{Cl_2}^{iPr}$. S25
Figure S21. $^{13}C\{^1H\}$ NMR spectrum of $\mathbf{1}_{Cl_2}^{iPr}$. S26
Figure S22. $^{31}P\{^1H\}$ NMR spectrum of $\mathbf{1}_{Cl_2}^{iPr}$. S26
Figure S23. ^1H NMR spectrum of $\mathbf{1}_{I_2}^{iPr}$. S27
Figure S24. ^1H-^{13}C HSQC NMR spectrum of $\mathbf{1}_{I_2}^{iPr}$. S27
Figure S25. $^{13}C\{^1H\}$ NMR spectrum of $\mathbf{1}_{I_2}^{iPr}$. S28
Figure S26. $^{31}P\{^1H\}$ NMR spectrum of $\mathbf{1}_{I_2}^{iPr}$. S28
Figure S27. ^1H NMR spectrum of $\mathbf{2}_{Cl_2}^{iPr}$. S29
Figure S28. ^1H-^{13}C HSQC NMR spectrum of $\mathbf{2}_{Cl_2}^{iPr}$. S29

Figure S29. $^{13}C\{^1H\}$ NMR spectrum of $\mathbf{2}^{iPr}_{Cl_2}$. S30
Figure S30. $^{31}P\{^1H\}$ NMR spectrum of $\mathbf{2}^{iPr}_{Cl_2}$. S30
Figure S31. ^1H NMR spectrum of $\mathbf{2}^{iPr}_{I_2}$. S31
Figure S32. ^1H-^{13}C HSQC NMR spectrum of $\mathbf{2}^{iPr}_{I_2}$. S31
Figure S33. $^{13}C\{^1H\}$ NMR spectrum of $\mathbf{2}^{iPr}_{I_2}$. S32
Figure S34. $^{31}P\{^1H\}$ NMR spectrum of $\mathbf{2}^{iPr}_{I_2}$. S32
Figure S35. ^1H NMR spectrum of $\mathbf{3}^{iPr}_{Cl}$. S33
Figure S36. ^1H-^{13}C HSQC NMR spectrum of $\mathbf{3}^{iPr}_{Cl}$. S33
Figure S37. $^{13}C\{^1H\}$ NMR spectrum of $\mathbf{3}^{iPr}_{Cl}$. S34
Figure S38. $^{31}P\{^1H\}$ NMR spectrum of $\mathbf{3}^{iPr}_{Cl}$. S34
Figure S39. ^1H NMR spectrum of $\mathbf{3}^{iPr}_{I}$. S35
Figure S40. ^1H-^{13}C HSQC NMR spectrum of $\mathbf{3}^{iPr}_{I}$. S35
Figure S41. $^{13}C\{^1H\}$ NMR spectrum of $\mathbf{3}^{iPr}_{I}$. S36
Figure S42. $^{31}P\{^1H\}$ NMR spectrum of $\mathbf{3}^{iPr}_{I}$. S36
Figure S43. ^1H NMR spectrum of $\mathbf{3}^{iPr}_{dmso}$. S37
Figure S44. ^1H-^{13}C HSQC NMR spectrum of $\mathbf{3}^{iPr}_{dmso}$. S37
Figure S45. $^{13}C\{^1H\}$ NMR spectrum of $\mathbf{3}^{iPr}_{dmso}$. S38
Figure S46. $^{31}P\{^1H\}$ NMR spectrum of $\mathbf{3}^{iPr}_{dmso}$. S38
Figure S47. $^{19}F\{^1H\}$ NMR spectrum of $\mathbf{3}^{iPr}_{dmso}$. S39
Figure S48. ^{31}P NMR monitoring of the conversion of chlorido complex [$RuCl_2$(η^6-methylbenzoate)(dimethyl(1-pyrenyl)phosphane)] ($\mathbf{1}^{Me}_{Cl_2}$) to iodido complex [RuI_2(η^6-methylbenzoate)(dimethyl(1-pyrenyl)phosphane)] ($\mathbf{1}^{Me}_{I_2}$). S39
Figure S49. ^1H NMR spectrum of $\mathbf{1}^{Me}_{I_2}$. S40
Figure S50. $^{13}C\{^1H\}$ NMR spectrum of $\mathbf{1}^{Me}_{I_2}$. S40
Figure S51. $^{31}P\{^1H\}$ NMR spectrum of $\mathbf{1}^{Me}_{I_2}$. S41
Figure S52. ^1H NMR spectrum of $\mathbf{2}^{Me}_{I_2}$. S41
Figure S53. ^1H-^{13}C HSQC NMR spectrum of $\mathbf{2}^{Me}_{I_2}$. S42
Figure S54. $^{13}C\{^1H\}$ NMR spectrum of $\mathbf{2}^{Me}_{I_2}$. S42
Figure S55. $^{31}P\{^1H\}$ NMR spectrum of $\mathbf{2}^{Me}_{I_2}$. S43
Cartesian coordinates (computational calculations) S44-S63

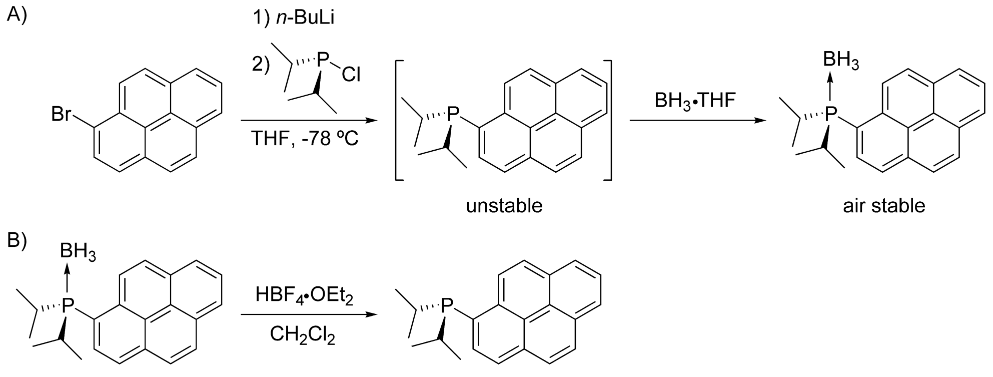

Scheme S1. A) Synthetic pathway for the preparation of diisopropyl(1-pyrenyl)-phosphane (**L**) and its air-stable borane complex; B) Procedure employed to deprotect **L**·BH_3.

Table S1. Crystal data and structure refinement for compounds $\mathbf{1}_{\mathrm{Cl_2}}^{iPr}$ (CCDC **2054649**) and $\mathbf{1}_{\mathrm{I_2}}^{iPr}$ (CCDC **2054650**).

Compound	$\mathbf{1}_{\mathrm{Cl_2}}^{iPr}$	$\mathbf{1}_{\mathrm{I_2}}^{iPr}$
Empirical formula	$C_{28.68}H_{29.68}Cl_2O_{1.67}PRu$, $0.16C_8H_8O_2$	$C_{30}H_{31}I_2O_2PRu$
Formula weight (g mol^{-1})	626.49	809.39
Temperature (K)	100(2)	100(2)
Crystal system	monoclinic	monoclinic
Space group	$P2_1$/c	$P2_1$/c
Crystal size (mm^3)	0.14 × 0.07 × 0.03	0.38 × 0.14 × 0.08
a (Å)	13.6602(4)	13.7725(5)
b (Å)	13.8972(3)	13.9773(5)
c (Å)	13.8290(3)	14.5403(5)
α (°)	90	90
β (°)	93.154(1)	93.528(2)
γ (°)	90	90
V (Å^3)	2621(1)	2794(1)
Z	4	4
ρ_{calcd}	1.587	1.924
μ (mm^{-1})	0.891	2.855
F(000)	1280	1568
ϑ for data collection (°)	2.987 – 30.034	2.964 – 22.986
Reflections collected / unique	30582 / 7668	15237 / 3866
Completeness to theta	0.998	0.996
Data / restraints / parameters	7668 / 322 / 330	4419 / 556 / 330
Goodness-of-fit on F^2	1.054	1.052
Final *R* indices [$I>2\sigma(I)$]	R1 = 0.0347, wR2 = 0.0887	R1 = 0.0388, wR2 = 0.0874
R indices (all data)	R1 = 0.0410, wR2 = 0.0927	R1 = 0.0444, wR2 = 1.077
largest diff. peak and hole (*e* Å^3)	2.284 and –0.815	2.619 and –1.447

Table S2. Crystal data and structure refinement for compounds $\mathbf{2}^{iPr}_{\mathrm{Cl_2}}$ (CCDC **2054651**) and $\mathbf{2}^{iPr}_{\mathrm{I_2}}$ (CCDC **2054652**).

Compound	$\mathbf{2}^{iPr}_{\mathrm{Cl_2}}$	$\mathbf{2}^{iPr}_{\mathrm{I_2}}$
Empirical formula	$C_{32}H_{37}Cl_2PRu$, CH_2Cl_2	$C_{32}H_{37}I_2PRu$
Formula weight (g mol^{-1})	709.48	807.45
Temperature (K)	200(2)	100(2)
Crystal system	monoclinic	triclinic
Space group	C_2/c	*P*−*1*
Crystal size (mm^3)	0.10 × 0.05 × 0.03	0.03 × 0.02 × 0.02
a (Å)	23.402(3)	10.6572(5)
b (Å)	15.368(2)	11.6371(6)
c (Å)	17.659(2)	13.0806(7)
α (°)	90	70.482(4)
β (°)	92.631(4)	78.707(4)
γ (°)	90	76.379(4)
V (Å^3)	6344.2(14)	1473.82(14)
Z	8	2
ρ_{calcd}	1.486	1.820
μ (mm^{-1})	1.131	2.701
F(000)	2912	788
ϑ for data collection (°)	2.113 – 33.606	1.889 − 26.019
Reflections collected / unique	54377 / 9678	9597 / 5694
Completeness to theta	0.999	0.980
Data / restraints / parameters	9678 / 16 / 373	5694 / 0 / 332
Goodness-of-fit on F^2	1.030	1.039
Final *R* indices [$I>2\sigma(I)$]	R1 = 0.0406, wR2 = 0.1142	R1 = 0.0508, wR2 = 0.1034
R indices (all data)	R1 = 0.0543, wR2 = 0.1223	R1 = 0.0798, wR2 = 0.1165
largest diff. peak and hole (*e* Å^3)	2.173 and −1.505	0.883 and −1.441

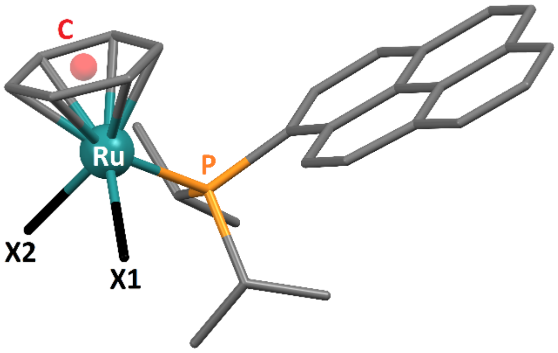

Figure S1. Representation of the crystal structures of compounds $\mathbf{1}^{iPr}_{\mathrm{Cl_2}}$, $\mathbf{1}^{iPr}_{\mathrm{I_2}}$, $\mathbf{2}^{iPr}_{\mathrm{Cl_2}}$ and $\mathbf{2}^{iPr}_{\mathrm{I_2}}$ with the atoms bond to the Ru centre labelled. ***C*** stands for the centroid of the arene ring.

Table S3. Selected bond distances (Å) and angles (°) for compounds $\mathbf{1}^{iPr}_{\mathrm{Cl_2}}$, $\mathbf{1}^{iPr}_{\mathrm{I_2}}$, $\mathbf{2}^{iPr}_{\mathrm{Cl_2}}$ and $\mathbf{2}^{iPr}_{\mathrm{I_2}}$. The atom labelling is shown in **Figure S1**.

	$\mathbf{1}^{iPr}_{\mathrm{Cl_2}}$	$\mathbf{1}^{iPr}_{\mathrm{I_2}}$	$\mathbf{2}^{iPr}_{\mathrm{Cl_2}}$	$\mathbf{2}^{iPr}_{\mathrm{I_2}}$
Ru–P	2.3893(5)	2.3975(10)	2.3877(7)	2.406(2)
Ru–X1	2.4198(5)	2.7247(4)	2.4303(9)	2.7232(8)
Ru–X2	2.4122(5)	2.7190(4)	2.4046(8)	2.7287(8)
Ru–C	1.6963(13)	1.7140(18)	1.7195(13)	1.725(3)
X1–Ru–X2	86.06(2)	87.20(1)	85.52(3)	87.35(2)
X1–Ru–P	90.43(2)	93.32(3)	86.84(3)	91.72(5)
X2–Ru–P	88.18(2)	90.29(3)	88.76(3)	90.58(5)
X1–Ru–C	123.85(4)	122.22(7)	125.62(5)	123.54(12)
X2–Ru–C	124.34(4)	123.80(7)	126.15(5)	123.42(12)
P–Ru–C	130.89(4)	128.72(7)	130.01(5)	128.70(12)

Preparation of diisopropyl(1-pyrenyl)phosphane oxide

Diisopropyl(1-pyrenyl)phosphine (**L**) (796 mg, 2.5 mmol) was dissolved in 25 mL of THF and aqueous 30% dihydrogen peroxide solution (1 mL, ~10 mmol) was added; the resulting reaction mixture was stirred for 1 h. The solvent was removed under reduced pressure and the residue was extracted with chloroform/water. The combined organic phase was dried with anhydrous Na_2SO_4 and filtered. After removal of the solvent, the crude was recrystallized in chloroform/diethyl ether. The title compound was obtained as a white solid with a yield of 62% (520 mg).

IR: 2965, 2930, 2869, 1595, 1580, 1459 ($n_{P=O}$), 1253, 1170, 1138, 1023, 979, 868, 726, 691, 608 cm^{-1}. **$^{31}P\{^1H\}$ NMR** ($CDCl_3$, 162 MHz): δ +56.9 (s) ppm. **^1H NMR** ($CDCl_3$, 400 MHz): δ 9.55 (d, *J* = 9.2, 1H), 8.28-8.04 (m, 8H), 2.65 (sept, *J* = 7.2, 2H), 1.34 (dd, *J* = 14.8, 6.8, 6H), 1.11 (dd, *J* = 15.6, 7.2, 6H) ppm. **HRMS**: m/z calcd. for $[M+H]^+$ 335.1559, found 335.1561.

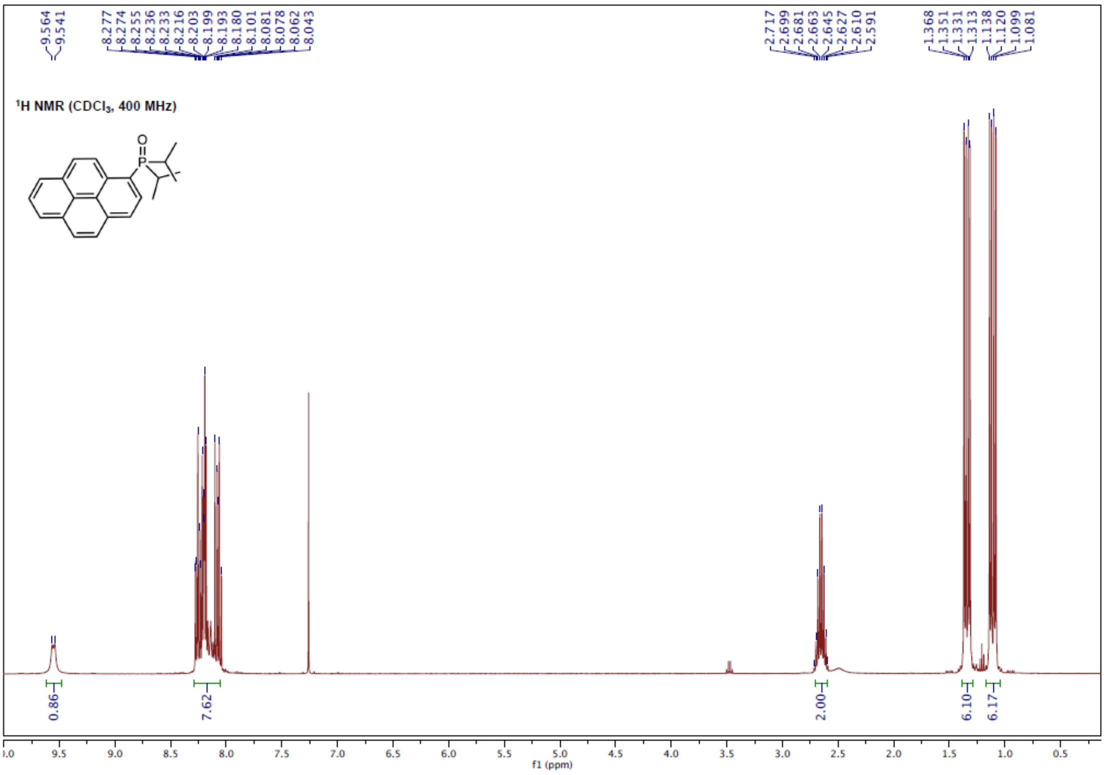

Figure S2. ^1H NMR spectrum of diisopropyl(1-pyrenyl)phosphane oxide.

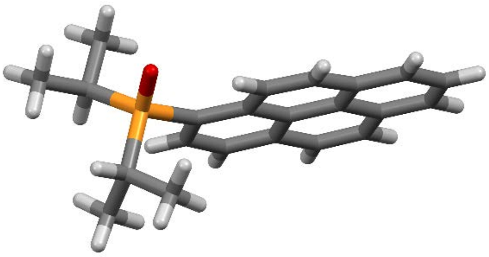

Figure S3. Representation of the crystal structure of diisopropyl(1-pyrenyl)phosphane oxide.

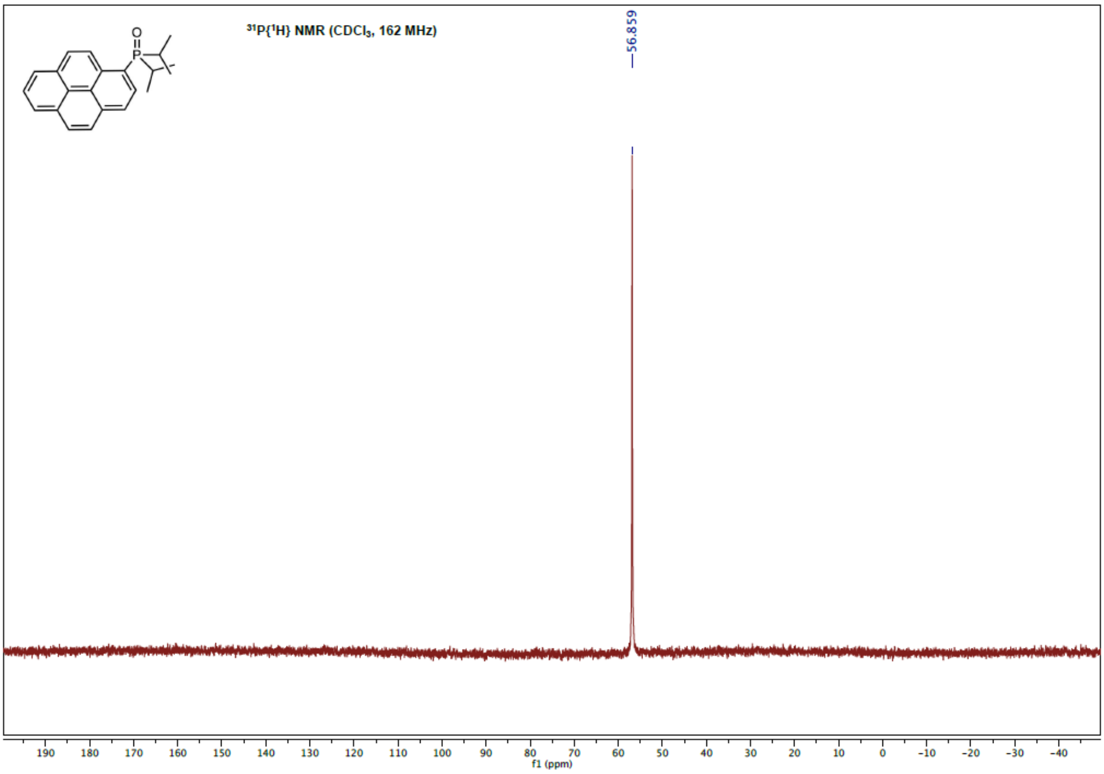

Figure S4. $^{31}P\{^{1}H\}$ NMR spectrum of diisopropyl(1-pyrenyl)phosphane oxide.

Table S4. Crystal data and structure refinement for diisopropyl(1-pyrenyl)phosphane oxide.

Compound	diisopropyl(1-pyrenyl)phosphane oxide
Empirical formula	$C_{22}H_{23}OP$
Formula weight (g mol^{-1})	334.37
Temperature (K)	296(2)
Crystal system	monoclinic
Space group	$P2_1/n$
Crystal size (mm^3)	0.22 × 0.13 × 0.12
a (Å)	12.5962(9)
b (Å)	10.4833(8)
c (Å)	14.0306(11)
α (°)	90
β (°)	109.794(5)
γ (°)	90
V (Å^3)	1743.3(2)
Z	4
ρ_{calcd}	1.274
μ (mm^{-1})	0.163
F(000)	712
ϑ for data collection (°)	2.59 – 26.92
Reflections collected / unique	14845 / 3850
Completeness to theta	0.997
Data / restraints / parameters	3850 / 0 / 221
Goodness-of-fit on F^2	1.063
Final *R* indices [I>2$\sigma(I)$]	R1 = 0.0452, wR2 = 0.1156
R indices (all data)	R1 = 0.0610, wR2 = 0.1243
largest diff. peak and hole (*e* Å^3)	0.259 and –0.290

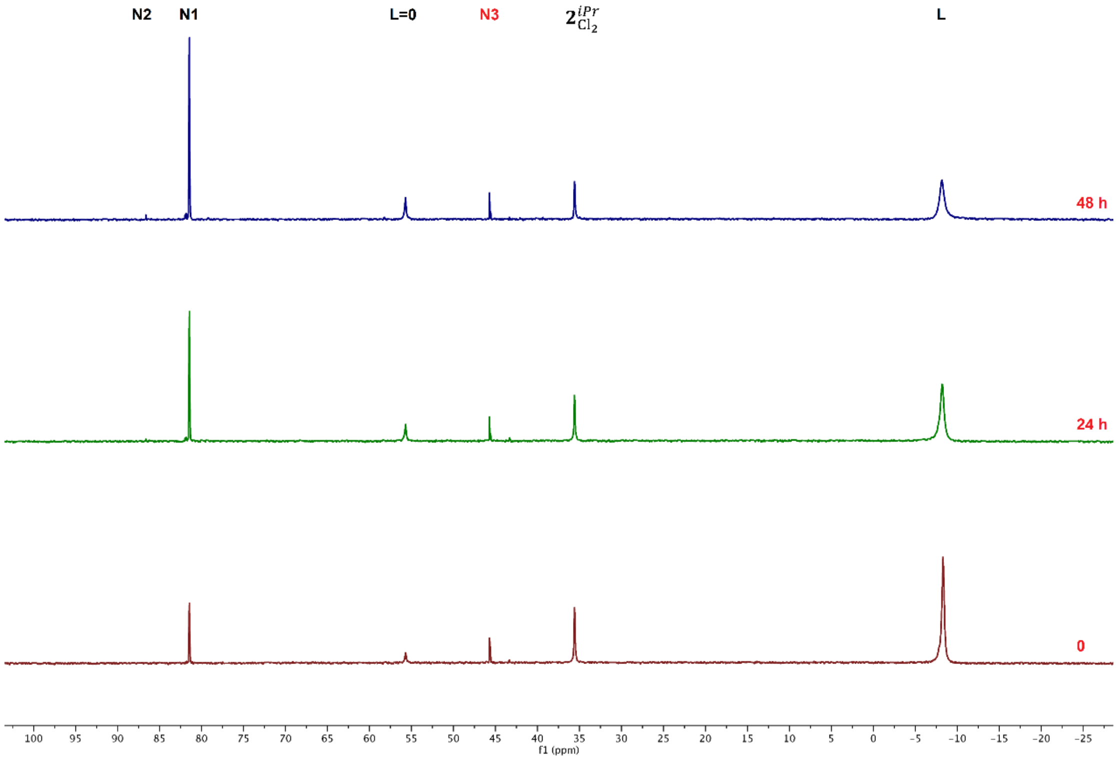

Figure S5. Time-dependent $^{31}P\{^1H\}$ NMR spectra of compound $\mathbf{2}^{iPr}_{Cl_2}$ in DMSO-d$_6$, recorded for two days.

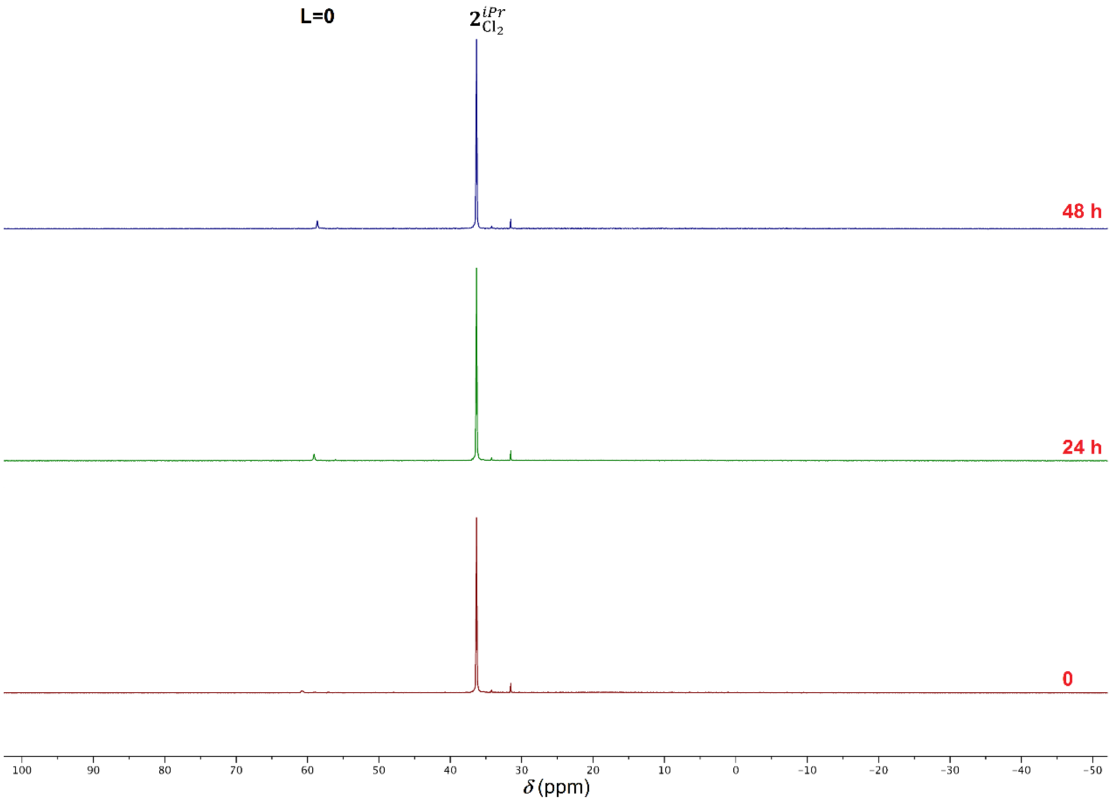

Figure S6. Time-dependent $^{31}P\{^1H\}$ NMR spectra of compound $\mathbf{2}^{iPr}_{Cl_2}$ in $CDCl_3$, recorded for two days.

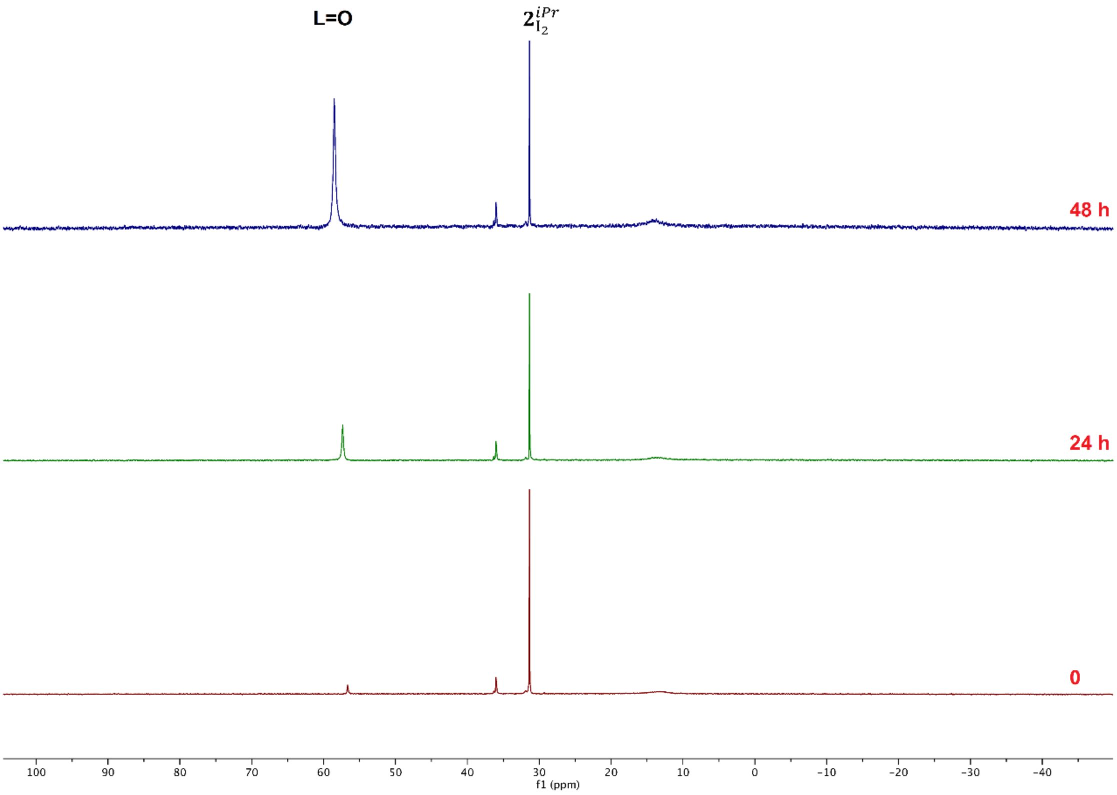

Figure S7. Time-dependent $^{31}P\{^1H\}$ NMR spectra of compound $\mathbf{2}_{I_2}^{iPr}$ in $CDCl_3$, recorded for two days.

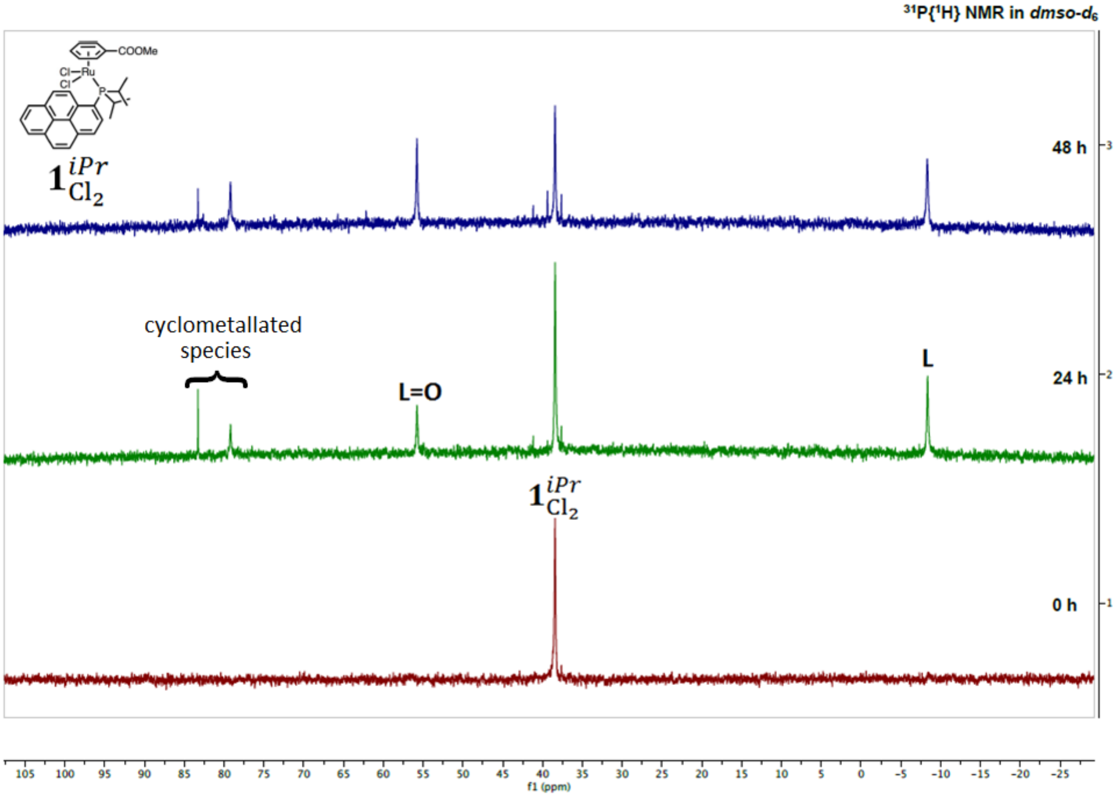

Figure S8. Time-dependent $^{31}P\{^1H\}$ NMR spectra of compound $\mathbf{1}_{Cl_2}^{iPr}$ in DMSO-d[6], recorded for two days.

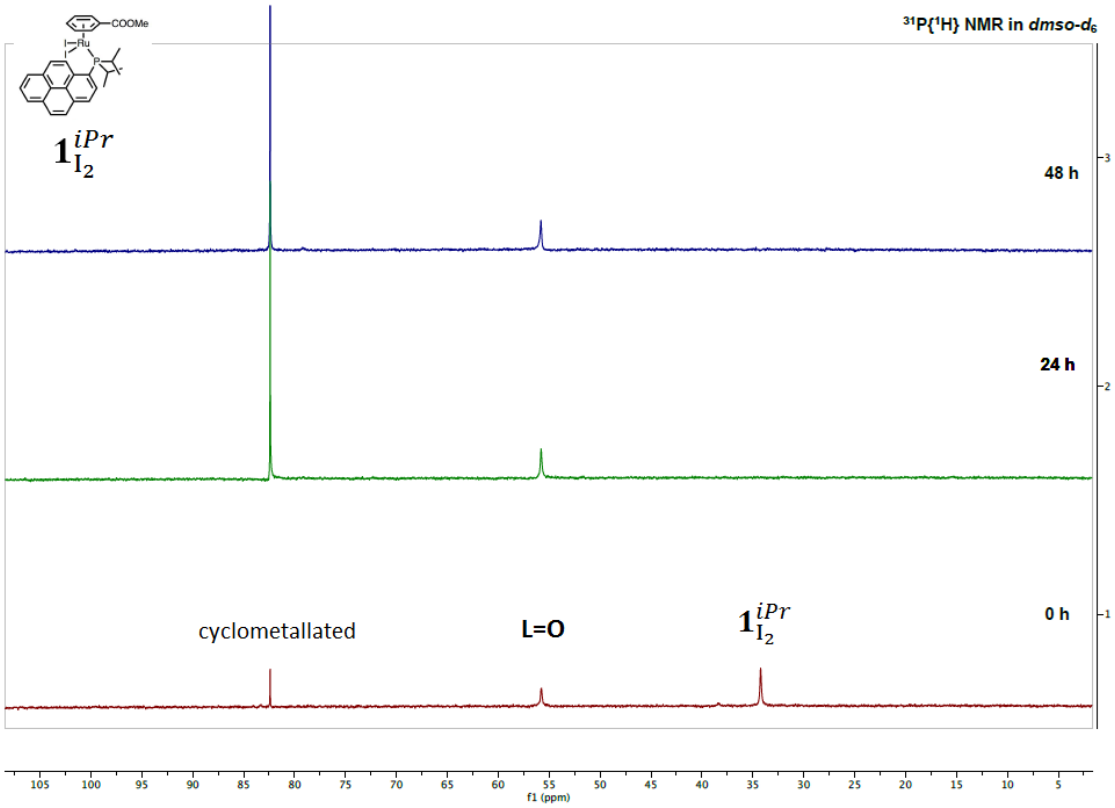

Figure S9. Time-dependent $^{31}P\{^{1}H\}$ NMR spectra of compound $\mathbf{1}^{iPr}_{I_2}$ in DMSO-d^6, recorded for two days.

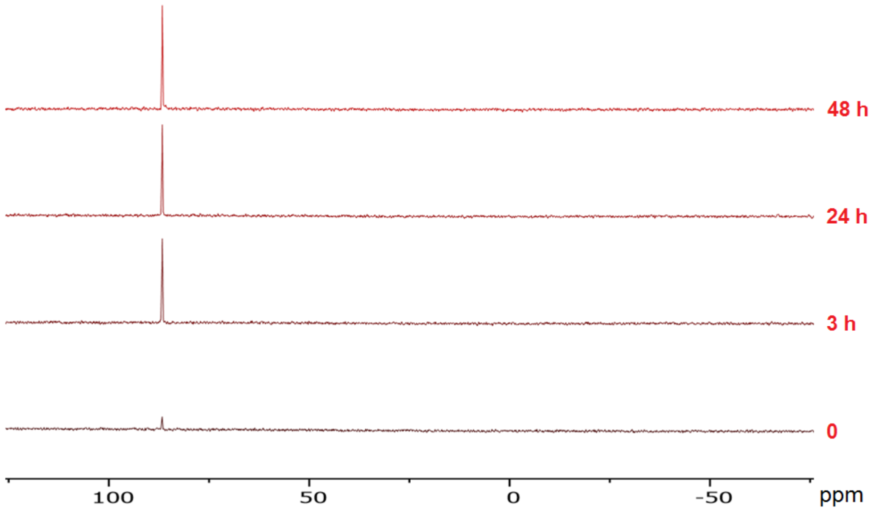

Figure S10. Time evolution of the $^{31}P\{^{1}H\}$ chemical shift of [Ru(η^6-*p*-cymene)(k^2C-diisopropyl(1-pyrenyl)phosphane)(dmso)]$^+$ for a 48 h-aged solution of complex $\mathbf{2}^{iPr}_{I_2}$ (spectra recorded in DMSO-d^6:D_2O 25:75).

Table S5. Crystal data and structure refinement for compounds $\mathbf{3}_{\mathrm{Cl}}^{iPr}$ (CCDC **2054653**) and $\mathbf{3}_{\mathrm{I}}^{iPr}$ (CCDC **2054654**).

Compound	$\mathbf{3}_{\mathrm{Cl}}^{iPr}$	$\mathbf{3}_{\mathrm{I}}^{iPr}$
Empirical formula	$C_{32}H_{36}ClPRu$	$C_{32}H_{36}IPRu$
Formula weight (g mol^{-1})	588.10	679.55
Temperature (K)	100(2)	296(2)
Crystal system	monoclinic	monoclinic
Space group	$P2_1/c$	$P2_1/c$
Crystal size (mm^3)	0.35 × 0.15 × 0.14	0.35 × 0.30 × 0.20
a (Å)	9.9414(5)	8.0137(3)
b (Å)	12.9703(7)	30.5251(11)
c (Å)	20.5363(11)	11.1355(4)
α (°)	90	90
β (°)	102.127(3)	98.013(2)
γ (°)	90	90
V (Å^3)	2588.9(2)	2697.36(17)
Z	4	4
ρ_{calcd}	1.509	1.673
μ (mm^{-1})	0.791	1.805
F(000)	1216	1360
ϑ for data collection (°)	1.87 – 27.56	2.57 – 27.51
Reflections collected / unique	23159 / 5919	24858 / 6175
Completeness to theta	0.991	0.995
Data / restraints / parameters	5919 / 0 / 375	6175 / 0 / 323
Goodness-of-fit on F^2	1.055	1.126
Final *R* indices [$I>2\sigma(I)$]	R1 = 0.0303, wR2 = 0.0755	R1 = 0.0236, wR2 = 0.0486
R indices (all data)	R1 = 0.0366, wR2 = 0.0789	R1 = 0.0257, wR2 = 0.0493
largest diff. peak and hole (*e* Å^3)	1.02 and –0.55	0.63 and –0.62

Table S6. Selected bond distances (Å) and angles (°) for compounds $\mathbf{3}_{\mathrm{Cl}}^{iPr}$ and $\mathbf{3}_{\mathrm{I}}^{iPr}$. The atom labelling is shown in **Figure 5** (Main Text).

	$\mathbf{3}_{\mathrm{Cl}}^{iPr}$		$\mathbf{3}_{\mathrm{I}}^{iPr}$
Ru−P	2.3057(7)	Ru−P	2.3132(8)
Ru−Cl1	2.4183(6)	Ru−I1	2.7156(4)
Ru−C8	2.084(2)	Ru−C11	2.088(2)
Ru−*C*	1.7432(10)	Ru−*C*	1.7635(11)
Cl1−Ru−C8	82.20(6)	I1−Ru−C11	83.78(5)
Cl1−Ru−P	88.37(2)	I1−Ru−P	91.99(2)
C8−Ru−P	81.24(6)	C11−Ru−P	80.96(7)
Cl1−Ru−*C*	123.19(4)	I1−Ru−*C*	123.44(3)
C8−Ru−*C*	132.21(7)	C11−Ru−*C*	128.71(8)
P−Ru−*C*	132.79(4)	P−Ru−*C*	132.45(4)

Table S7. Crystal data and structure refinement for compound $\mathbf{3}^{iPr}_{\mathrm{dmso}}$ (CCDC **2054655**).[a]

Compound	$\mathbf{3}^{iPr}_{\mathrm{dmso}}$
Empirical formula	$C_{34}H_{42}OPRu S, F_6P$
Formula weight (g mol^{-1})	775.74
Temperature (K)	296(2)
Crystal system	monoclinic
Space group	$P2_1/c$
Crystal size (mm^3)	0.3 × 0.2 × 0.08
a (Å)	24.6344(12)
b (Å)	21.6629(11)
c (Å)	16.6461(8)
α (°)	90
β (°)	105.780(2)
γ (°)	90
V (Å^3)	8548.5(7)
Z	8
ρ_{calcd}	1.206
μ (mm^{-1})	0.538
F(000)	3184
ϑ for data collection (°)	0.859 – 21.966
Reflections collected / unique	43524 / 10403
Completeness to theta	0.997
Data / restraints / parameters	10403 / 134 / 793
Goodness-of-fit on F^2	1.816
Final *R* indices [I>2σ(I)]	R1 = 0.1385, wR2 = 0.4187
R indices (all data)	R1 = 0.1751, wR2 = 0.4458
largest diff. peak and hole (*e* Å^3)	2.88 and −1.19

[a]The crystallographic data are of moderate quality due to poor diffraction of the tiny single crystals of $\mathbf{3}^{iPr}_{\mathrm{dmso}}$. However, these data are enough to corroborate the nature of the coordination environment of the Ru centre.

Table S8. Selected bond distances (Å) and angles (°) for compound $\mathbf{3}^{iPr}_{\mathrm{dmso}}$. The atom labelling is shown in **Figure 5** (Main Text).

	$\mathbf{3}^{iPr}_{\mathrm{dmso}}$
Ru−P	2.326(5)
Ru−S	2.243(5)
Ru−C45	2.077(19)
Ru−*C*	1.785(7)
S−Ru−P	94.82(17)
C45−Ru−S	84.8(6)
C45−Ru−P	80.1(6)
S−Ru−*C*	128.4(3)
C45−Ru−*C*	124.9(6)
P−Ru−*C*	128.1(3)

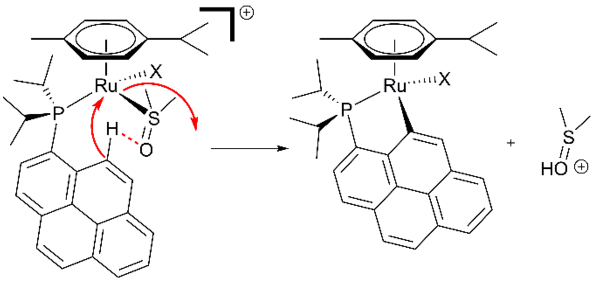

Intermediate **A** $\mathbf{3}_{\mathrm{Cl}}^{iPr}$, X = Cl; $\mathbf{3}_{\mathrm{I}}^{iPr}$, X = I

Figure S11. Proposed mechanism for the DMSO-mediated (O-donor Lewis base) cyclometallation reaction.

Table S9. Observed rate constants for the solvation of compound $\mathbf{3}_{\mathrm{Cl}}^{iPr}$ in various solvents as a function of the temperature and pressure. [Complex] = 10–50 µM.

Compound	Solvent	T (°C)	P (atm)	k_{obs} (s^{-1})
$\mathbf{3}_{\mathrm{Cl}}^{iPr}$	DMSO	40	1	$5.0 \cdot 10^{-6}$
		50	1	$1.2 \cdot 10^{-5}$
		60	1	$3.2 \cdot 10^{-5}$
		69	400	$9.2 \cdot 10^{-5}$
		69	700	$7.9 \cdot 10^{-5}$
			1000	$8.0 \cdot 10^{-5}$
			1300	$7.3 \cdot 10^{-5}$
			1600	$7.1 \cdot 10^{-5}$
			400	$9.2 \cdot 10^{-5}$
			700	$7.9 \cdot 10^{-5}$
		70	1	$9.5 \cdot 10^{-5}$
$\mathbf{3}_{\mathrm{Cl}}^{iPr}$	DMSO saturated with NaCl	70	1	$9.0 \cdot 10^{-5}$
$\mathbf{3}_{\mathrm{Cl}}^{iPr}$	*n*-Butanol	50	1	$7.3 \cdot 10^{-5}$
		55	400	$9.2 \cdot 10^{-5}$
		55	700	$7.5 \cdot 10^{-5}$
			1000	$7.5 \cdot 10^{-5}$
			1300	$5.9 \cdot 10^{-5}$
			1600	$6.0 \cdot 10^{-5}$
			1800	$4.3 \cdot 10^{-5}$
		60	1	$1.5 \cdot 10^{-4}$
		70	1	$3.0 \cdot 10^{-4}$
$\mathbf{3}_{\mathrm{Cl}}^{iPr}$	H_2O (dissolution process)[a]	60	1	$2.2 \cdot 10^{-4}$
		70	1	$3.7 \cdot 10^{-4}$
		80	1	$6.2 \cdot 10^{-4}$

[a] $\mathbf{3}_{\mathrm{Cl}}^{iPr}$ is poorly soluble in water. Thus, the parameters obtained correspond to its slow aquation (reflected by the appearance of time-resolved, definite UV-Vis spectra).

Table S10. Observed rate constants for the solvation of compounds $\mathbf{3}_{\mathrm{I}}^{iPr}$ and $\mathbf{3}_{\mathrm{dmso}}^{iPr}$ in various solvents as a function of the temperature and pressure. [Complex] = 10–50 µM.

Compound	Solvent	T (°C)	P (atm)	k_{obs} (s^{-1})
$\mathbf{3}_{\mathrm{I}}^{iPr}$	DMSO	40	1	$2.3 \cdot 10^{-5}$
		50	1	$8.3 \cdot 10^{-5}$
		60	1	$2.8 \cdot 10^{-4}$
		60	400	$2.1 \cdot 10^{-4}$
			700	$1.9 \cdot 10^{-4}$
			1000	$1.6 \cdot 10^{-4}$
			1300	$1.4 \cdot 10^{-4}$
			1600	$1.2 \cdot 10^{-4}$
		64	1	$5.2 \cdot 10^{-4}$
		75	1	$2.0 \cdot 10^{-3}$
$\mathbf{3}_{\mathrm{I}}^{iPr}$	*n*-Butanol	30	1	$7.0 \cdot 10^{-5}$
		40	1	$1.7 \cdot 10^{-4}$
		50	1	$3.5 \cdot 10^{-4}$
		50	400	$3.1 \cdot 10^{-4}$
			700	$2.4 \cdot 10^{-4}$
			1000	$2.2 \cdot 10^{-4}$
			1300	$1.6 \cdot 10^{-4}$
			1600	$1.3 \cdot 10^{-4}$
			1900	$9.5 \cdot 10^{-5}$
		60	1	$7.0 \cdot 10^{-4}$
$\mathbf{3}_{\mathrm{dmso}}^{iPr}$	H_2O	40	1	$2.6 \cdot 10^{-5}$
		48	1	$4.7 \cdot 10^{-5}$
		55	1	$1.1 \cdot 10^{-4}$
		60	1	$1.1 \cdot 10^{-4}$
		61	400	$1.7 \cdot 10^{-4}$
		61	550	$1.9 \cdot 10^{-4}$
			700	$1.9 \cdot 10^{-4}$
			1000	$2.1 \cdot 10^{-4}$
			1150	$2.7 \cdot 10^{-4}$
			1300	$2.9 \cdot 10^{-4}$
			1600	$2.8 \cdot 10^{-4}$
			1800	$3.7 \cdot 10^{-4}$
		70	1	$2.8 \cdot 10^{-4}$
		75	1	$3.7 \cdot 10^{-4}$
		80	1	$7.1 \cdot 10^{-4}$

Table S11. Crystal data and structure refinement for compounds $\mathbf{1}_{I_2}^{Me}$ (CCDC **2054656**) and $\mathbf{2}_{I_2}^{Me}$ (CCDC 2054657).

Compound	$\mathbf{1}_{I_2}^{Me}$	$\mathbf{2}_{I_2}^{Me}$
Empirical formula	$C_{26}H_{23}I_2O_2PRu$	$C_{28}H_{29}I_2O_2PRu$
Formula weight (g mol^{-1})	753.28	751.35
Temperature (K)	100(2)	100(2)
Crystal system	monoclinic	orthorhombic
Space group	$P2_1/c$	$P2_12_12_1$
Crystal size (mm^3)	0.15 × 0.10 × 0.06	0.08 × 0.04 × 0.04
a (Å)	14.1777(12)	11.9747(6)
b (Å)	12.9115(11)	26.9780(13)
c (Å)	13.8121(13)	8.1180(4)
α (°)	90	90
β (°)	111.236(4)	90
γ (°)	90	90
V (Å^3)	2356.7(4)	2622.5(2)
Z	4	4
ρ_{calcd}	2.123	1.903
µ (mm^{-1})	3.376	3.198
F(000)	1440	1448
ϑ for data collection (°)	3.02 – 26.79	1.548 – 31.367
Reflections collected / unique	21170 / 5004	45719 / 8004
Completeness to theta	0.994	0.999
Data / restraints / parameters	5004 / 0 / 292	8004 / 63 / 294
Goodness-of-fit on F^2	1.058	1.106
Final *R* indices [$I>2\sigma(I)$]	R1 = 0.0478, wR2 = 0.1385	R1 = 0.0429, wR2 = 0.1106
R indices (all data)	R1 = 0.0536, wR2 = 0.1423	R1 = 0.0432, wR2 = 0.1109
largest diff. peak and hole (*e* Å^3)	1.021 and –0.547	3.853 and –1.707

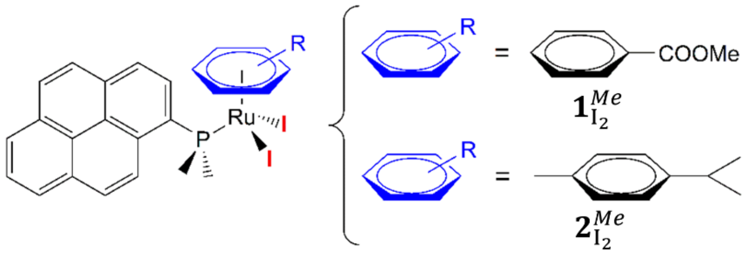

Scheme S2. Schematic representation of complexes $\mathbf{1}_{I_2}^{Me}$ and $\mathbf{2}_{I_2}^{Me}$.

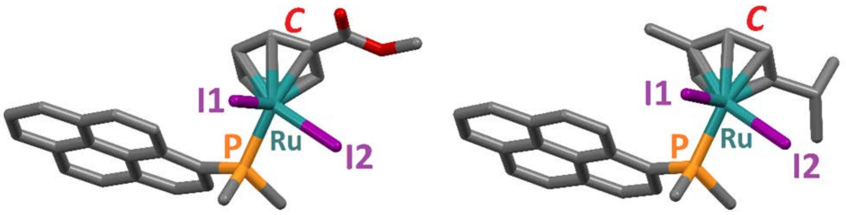

$\mathbf{1}_{I_2}^{Me}$ $\mathbf{2}_{I_2}^{Me}$

Figure S12. Representation of the crystal structures of compounds $\mathbf{1}_{I_2}^{Me}$ and $\mathbf{2}_{I_2}^{Me}$ with the atoms bonded to the Ru center labelled. ***C*** stands for the centroid of the arene ring.

Table S12. Selected bond distances (Å) and angles (°) for compounds $\mathbf{1}_{I_2}^{Me}$ and $\mathbf{2}_{I_2}^{Me}$. The atom labelling is shown in **Figure S12**.

	$\mathbf{1}_{I_2}^{Me}$	$\mathbf{2}_{I_2}^{Me}$
Ru–P	2.3403(1)	2.351(2)
Ru–I1	2.6722(1)	2.7160(7)
Ru–I2	2.7235(1)	2.7355(8)
Ru–*C*	1.6953(1)	1.698(3)
I1–Ru–I2	89.60(1)	86.51(2)
I1–Ru–P	92.11(1)	91.60(6)
I2–Ru–P	87.20(1)	86.75(6)
I1–Ru–*C*	123.05(1)	124.78(11)
I2–Ru–*C*	125.25(1)	126.20(10)
P–Ru–*C*	128.20(1)	128.25(12)

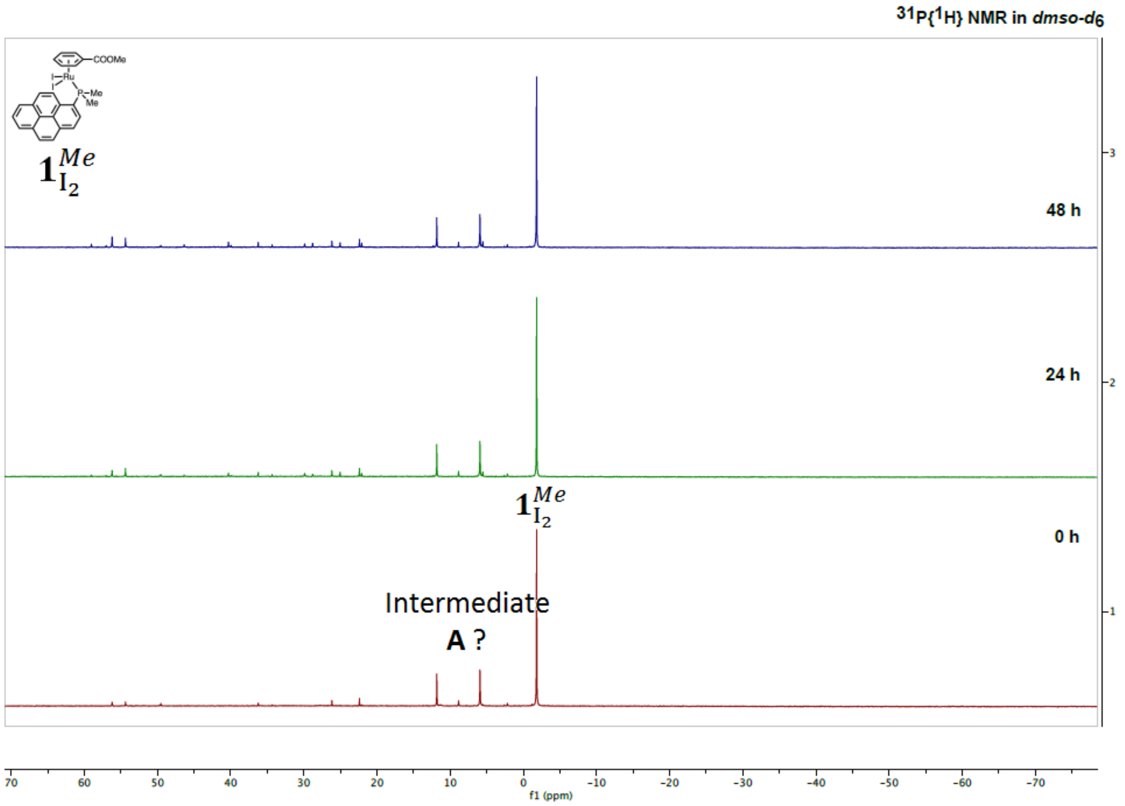

Figure S13. Time-dependent $^{31}P\{^1H\}$ NMR spectra of compound $\mathbf{1}_{I_2}^{Me}$ in DMSO-d[6], recorded for two days.

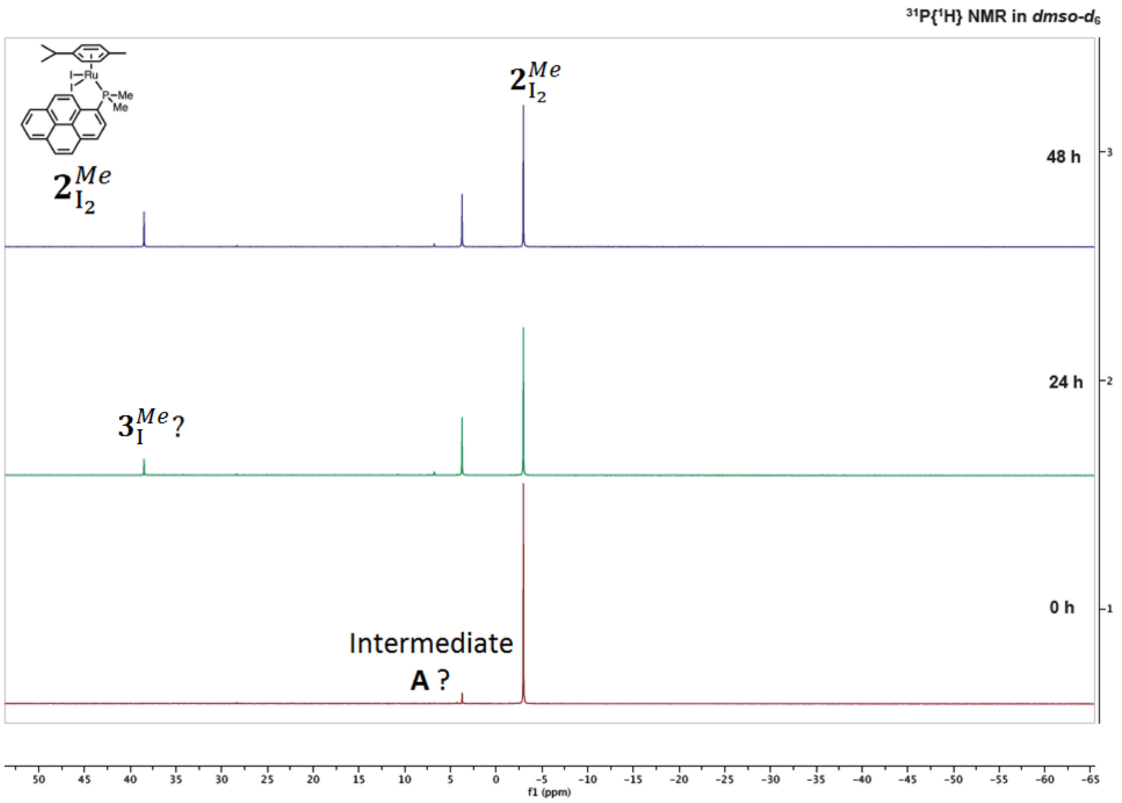

Figure S14. Time-dependent $^{31}P\{^1H\}$ NMR spectra of compound $\mathbf{2}_{I_2}^{Me}$ in DMSO-d[6], recorded for two days.

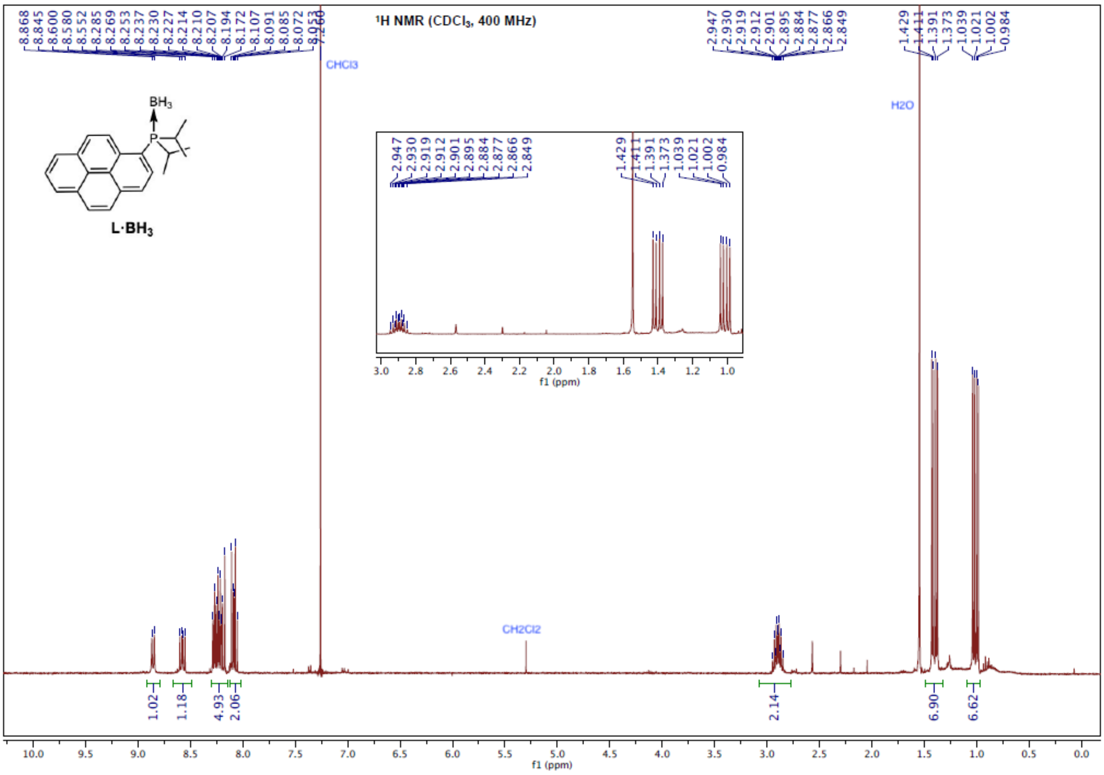

Figure S15. ^{1}H NMR spectrum of borane diisopropyl(1-pyrenyl)-phosphane complex (**L·BH₃**)

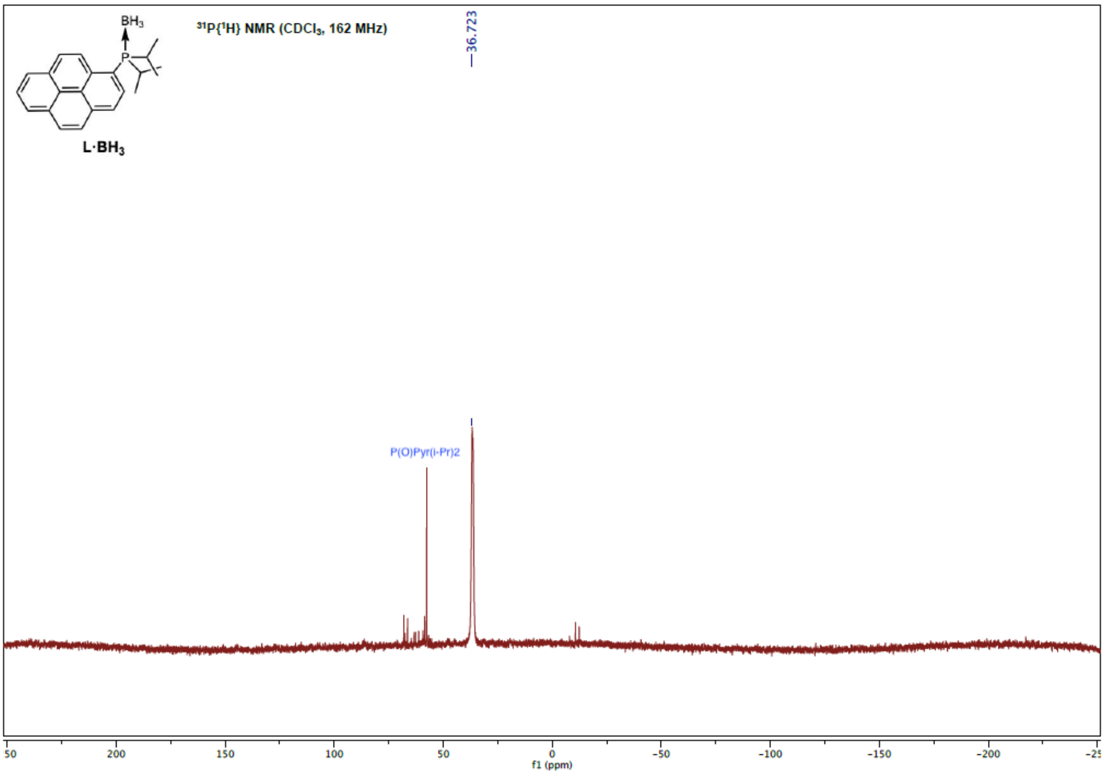

Figure S16. ^{31}P{^{1}H} NMR spectrum of borane diisopropyl(1-pyrenyl)-phosphane complex (**L·BH₃**)

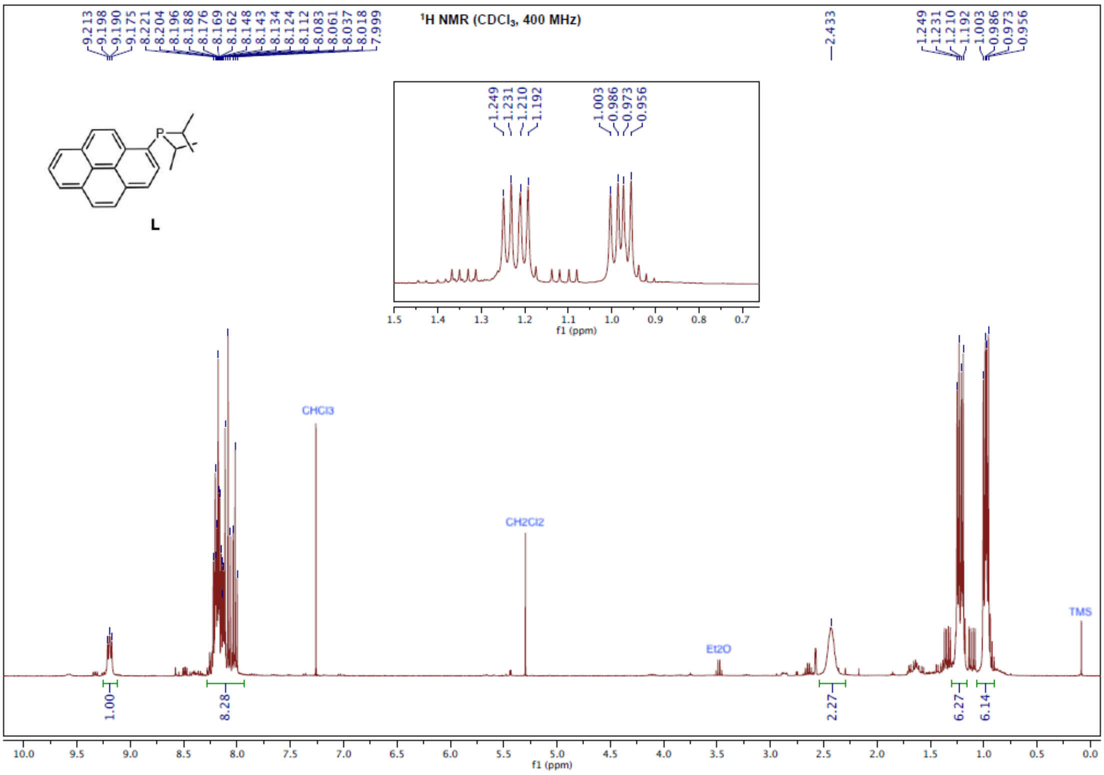

Figure S17. ^{1}H NMR spectrum of diisopropyl(1-pyrenyl)-phosphane (**L**)

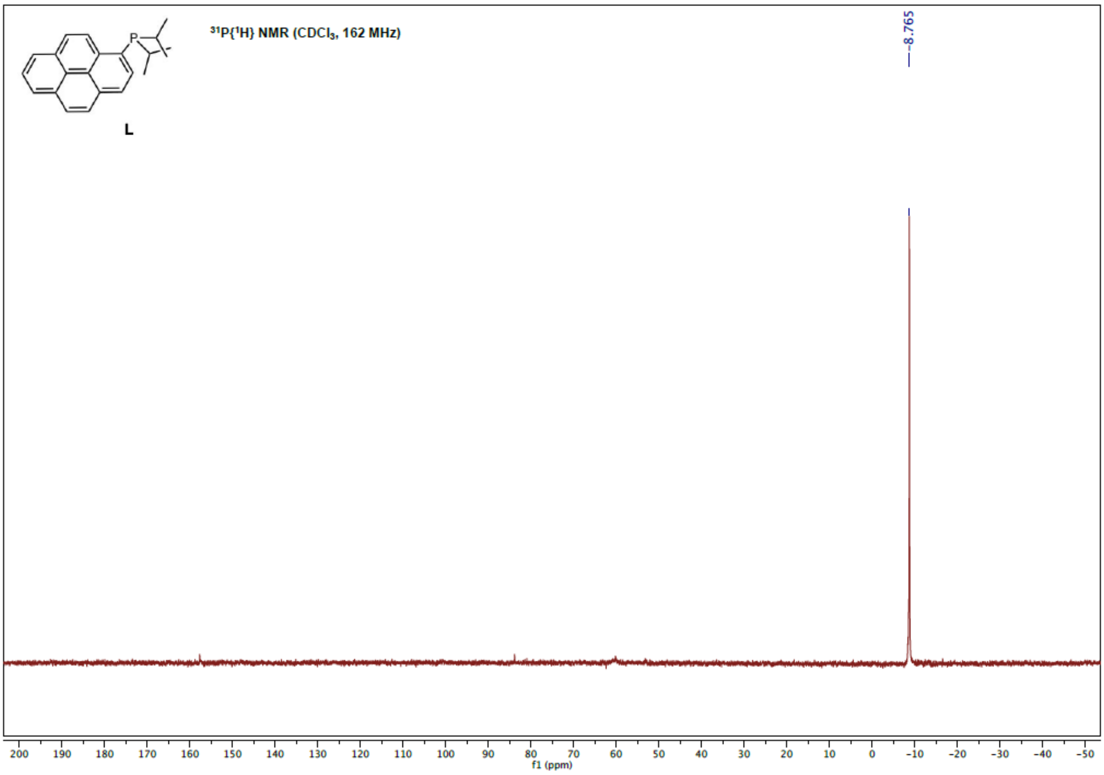

Figure S18. ^{31}P{^{1}H} NMR spectrum of diisopropyl(1-pyrenyl)-phosphane (**L**)

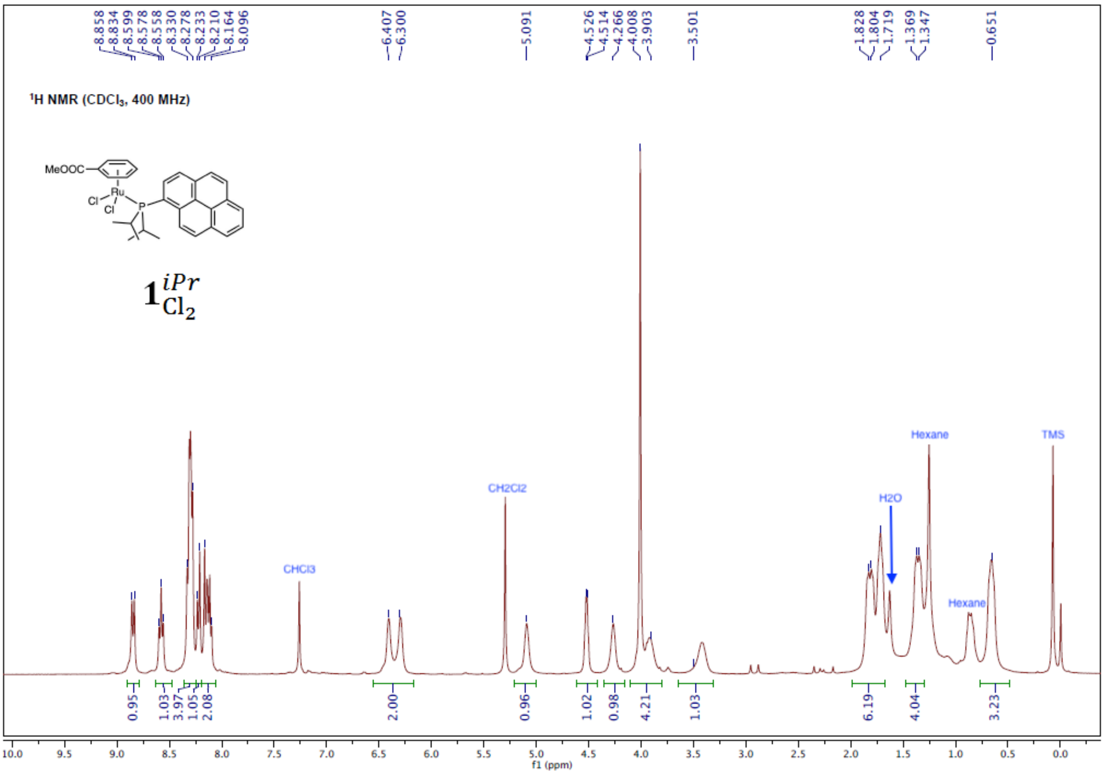

Figure S19. ^{1}H NMR spectrum of [RuCl$_2$(η^6-methylbenzoate)(diisopropyl(1-pyrenyl)phosphane)] ($\mathbf{1}^{iPr}_{Cl_2}$).

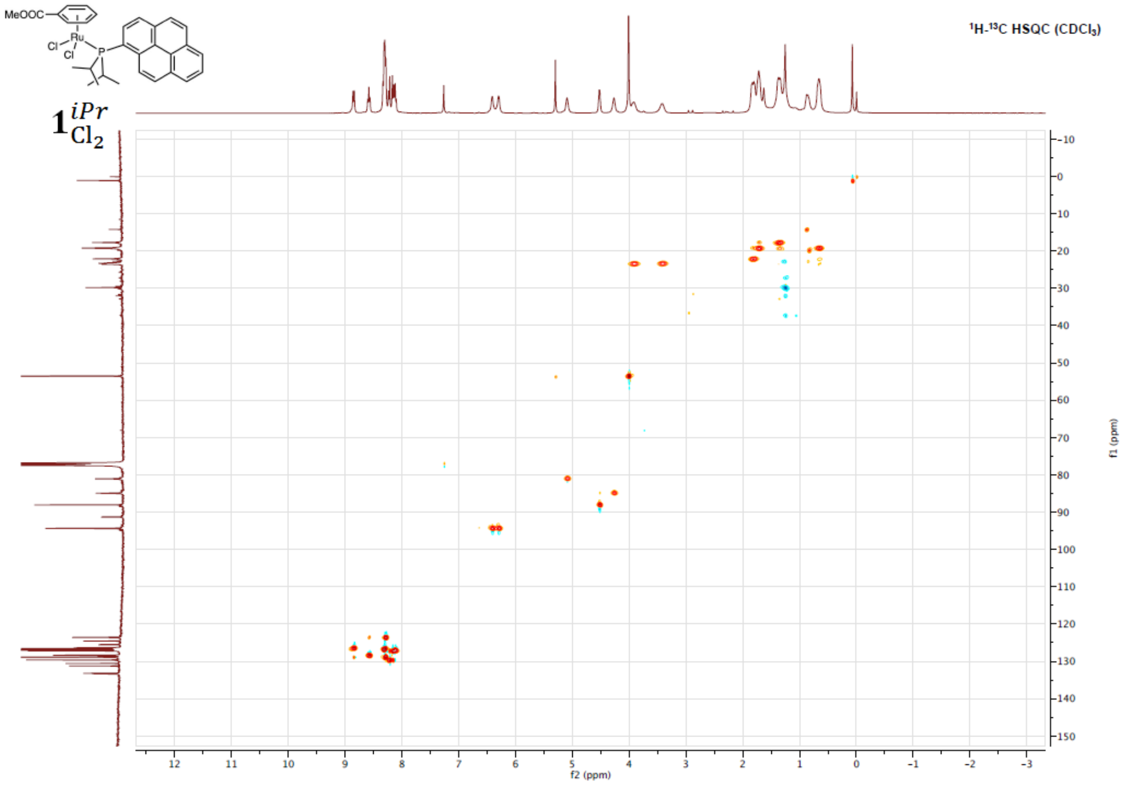

Figure S20. ^{1}H-^{13}C HSQC NMR spectrum of [RuCl$_2$(η^6-methylbenzoate)(diisopropyl(1-pyrenyl)phosphane)] ($\mathbf{1}^{iPr}_{Cl_2}$).

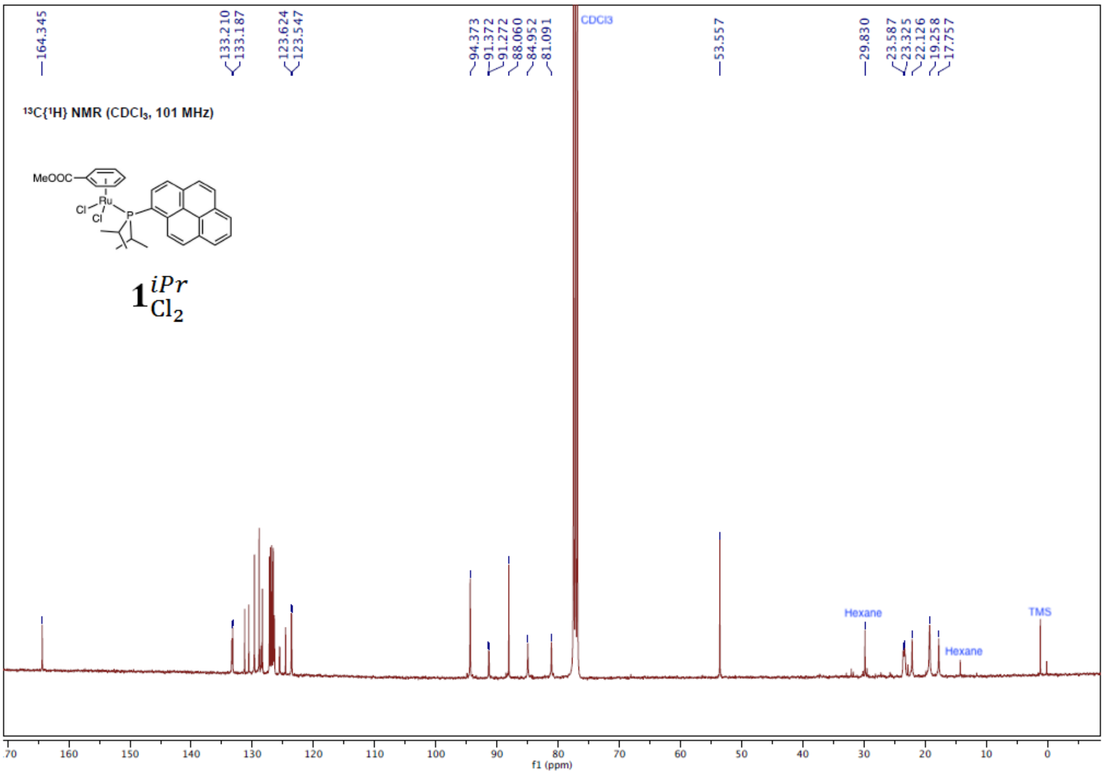

Figure S21. $^{13}C\{^1H\}$ NMR spectrum of [RuCl$_2$(η^6-methylbenzoate)(diisopropyl(1-pyrenyl)phosphane)] ($\mathbf{1}^{iPr}_{Cl_2}$).

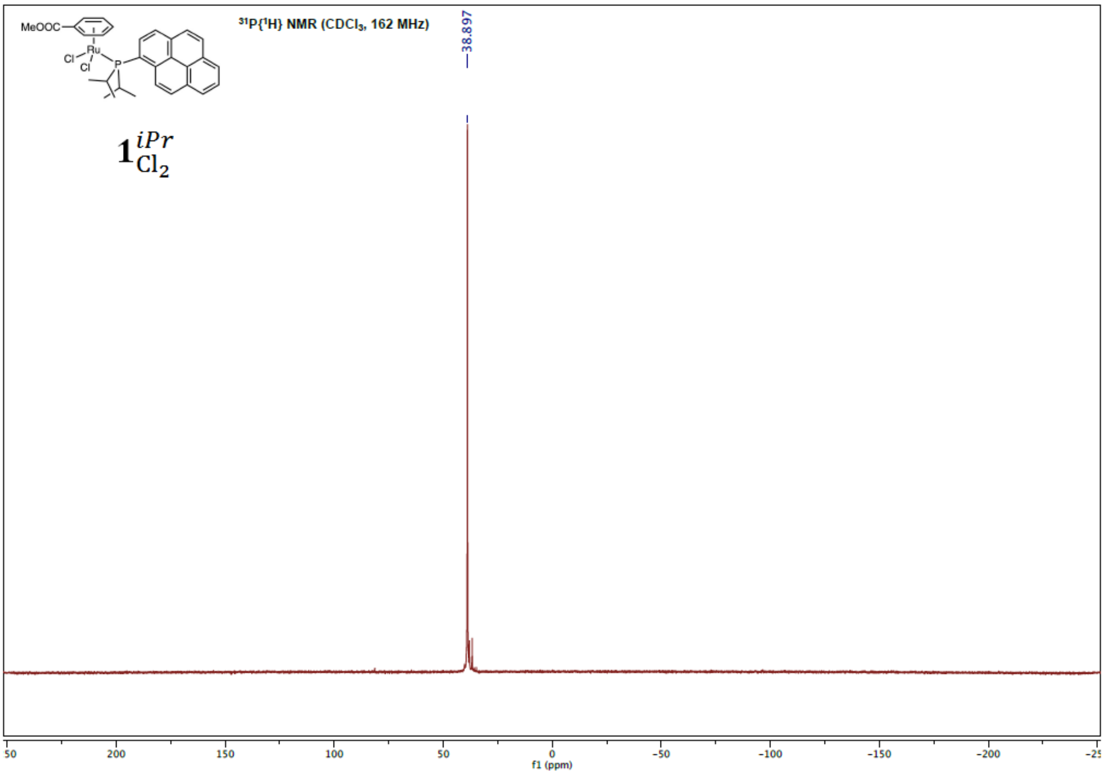

Figure S22. $^{31}P\{^1H\}$ NMR spectrum of [RuCl$_2$(η^6-methylbenzoate)(diisopropyl(1-pyrenyl)phosphane)] ($\mathbf{1}^{iPr}_{Cl_2}$).

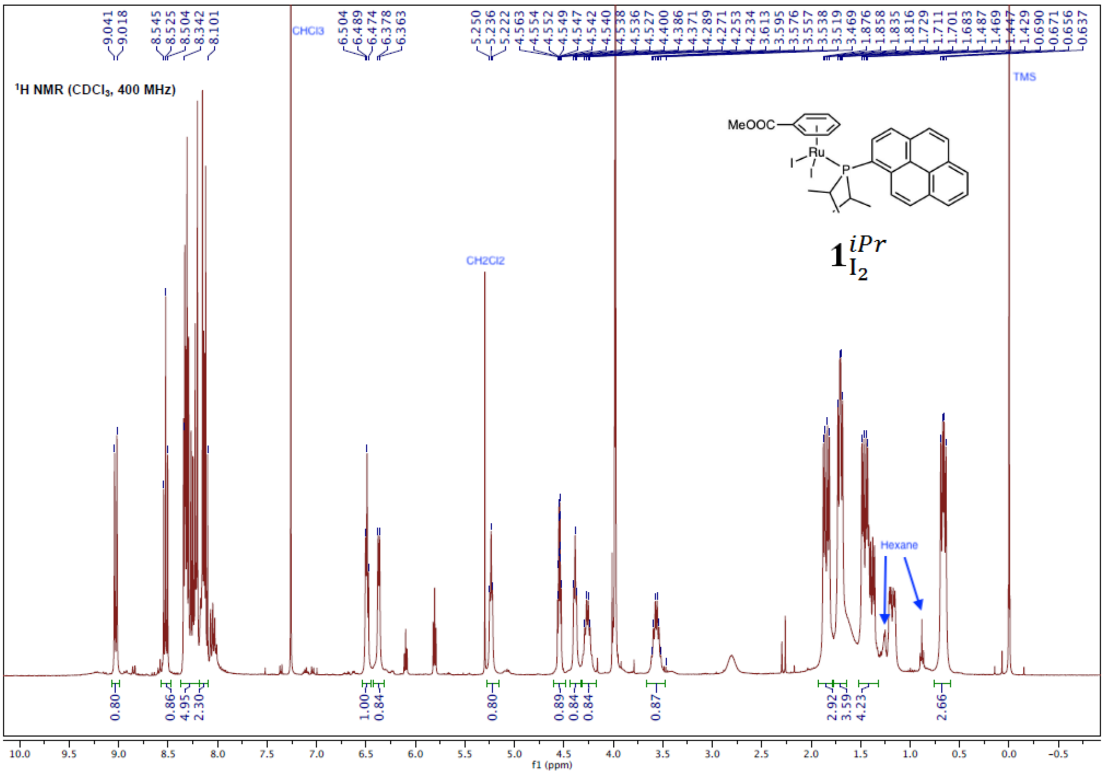

Figure S23. ^{1}H NMR spectrum of [RuI$_2$(η^6-methylbenzoate)(diisopropyl(1-pyrenyl)phosphane)] ($\mathbf{1}^{iPr}_{I_2}$).

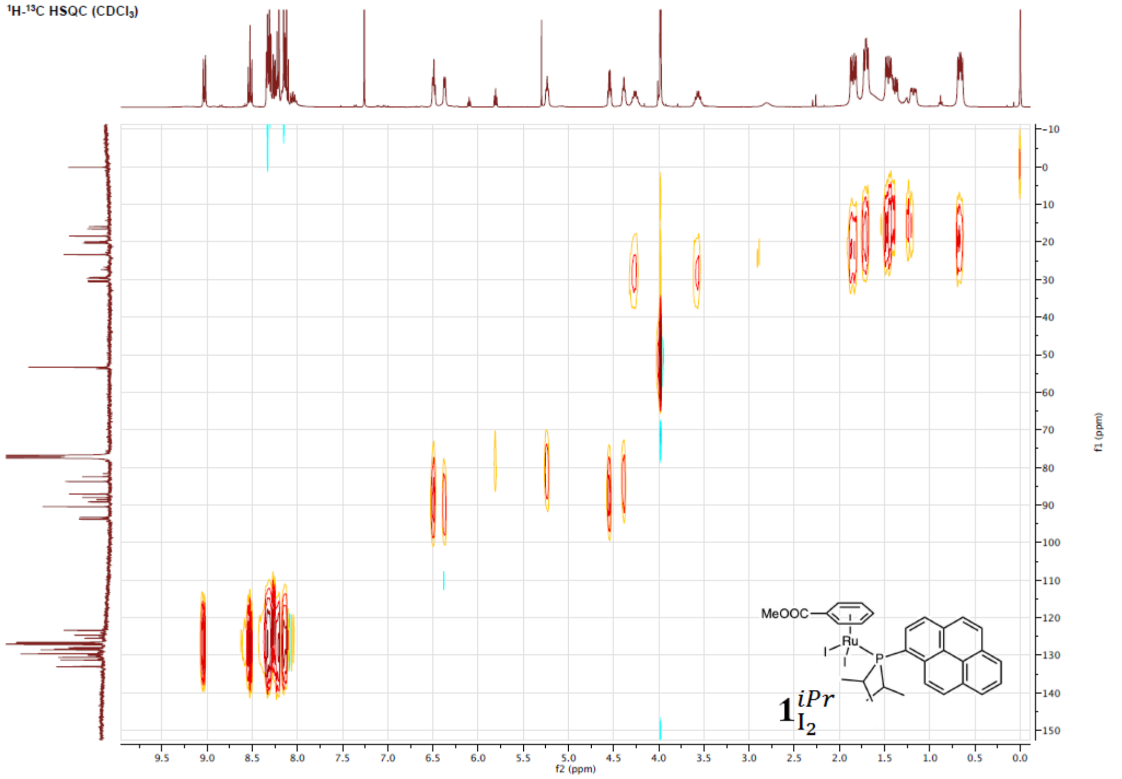

Figure S24. ^{1}H-^{13}C HSQC NMR spectrum of [RuI$_2$(η^6-methylbenzoate)(diisopropyl(1-pyrenyl)phosphane)] ($\mathbf{1}^{iPr}_{I_2}$).

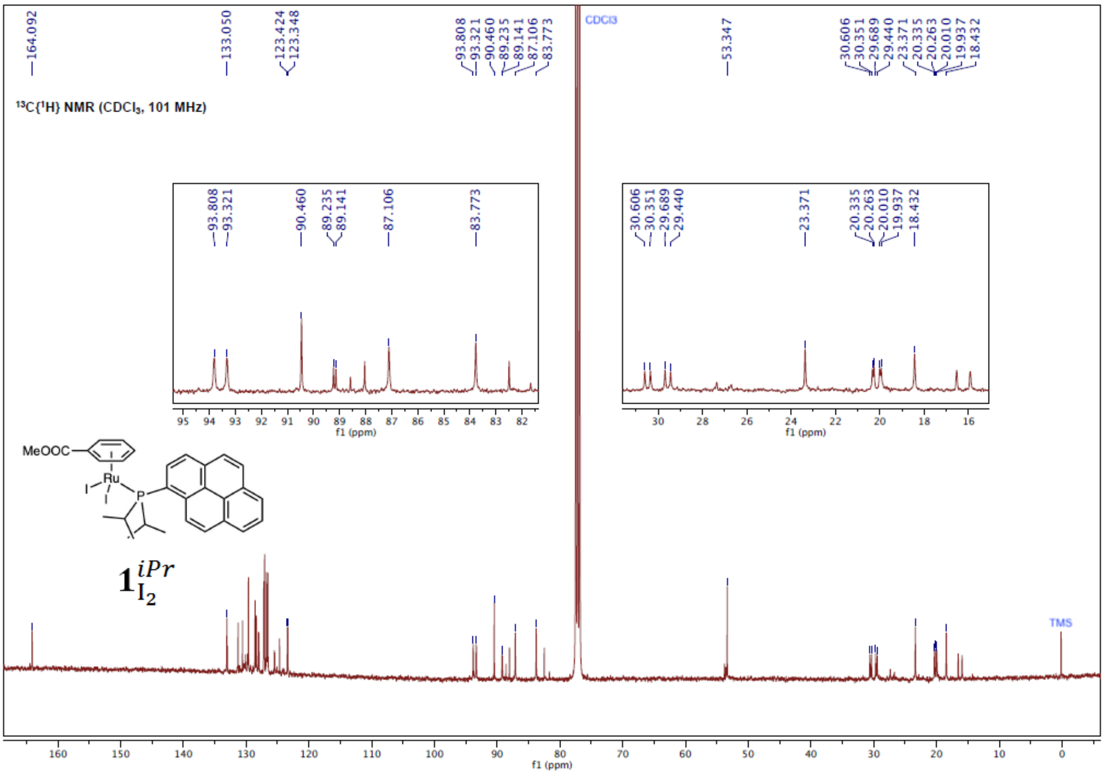

Figure S25. $^{13}C\{^{1}H\}$ NMR spectrum of [RuI_2(η^6-methylbenzoate)(diisopropyl(1-pyrenyl)phosphane)] ($\mathbf{1}^{iPr}_{I_2}$).

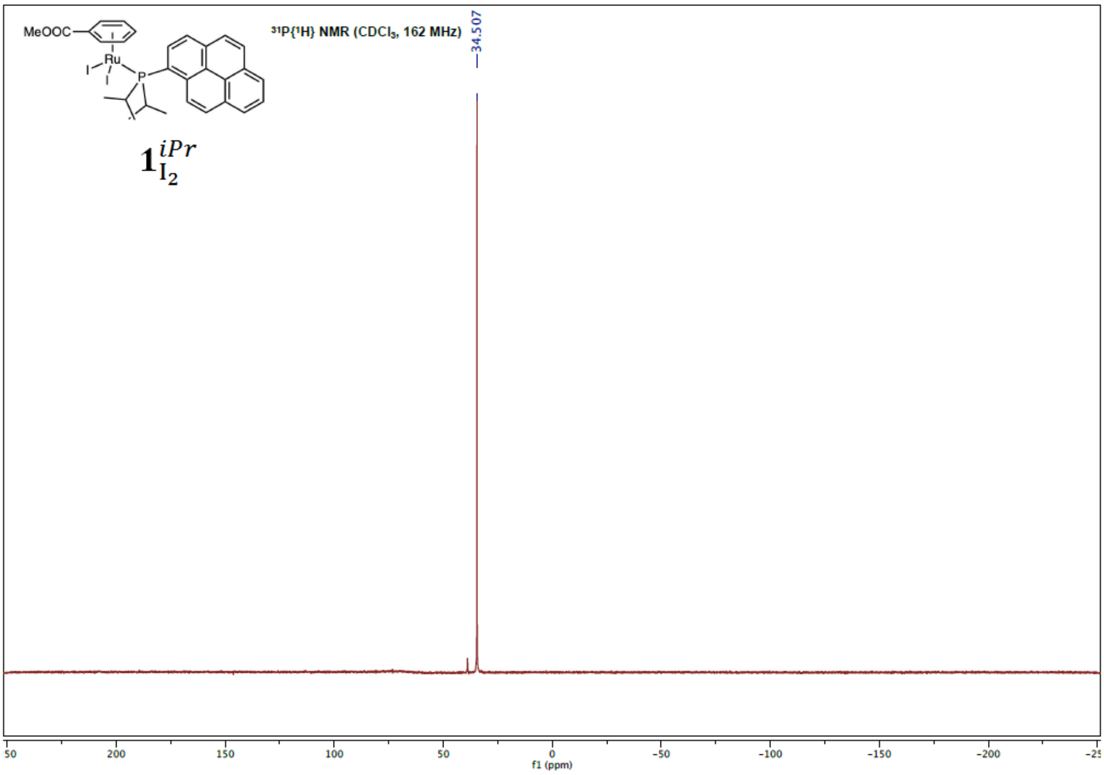

Figure S26. $^{31}P\{^{1}H\}$ NMR spectrum of [RuI_2(η^6-methylbenzoate)(diisopropyl(1-pyrenyl)phosphane)] ($\mathbf{1}^{iPr}_{I_2}$).

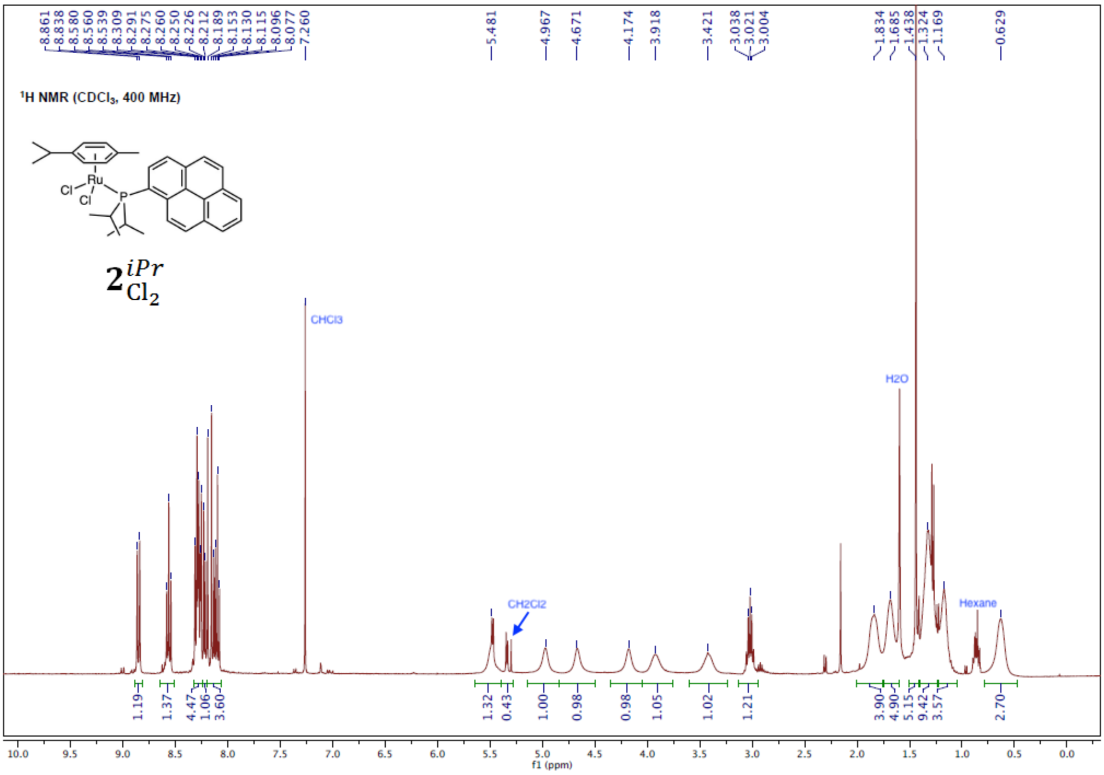

Figure S27. ^{1}H NMR spectrum of [RuCl$_2$(η^6-*p*-cymene)(diisopropyl(1-pyrenyl)phosphane)] ($\mathbf{2}^{iPr}_{\mathrm{Cl_2}}$).

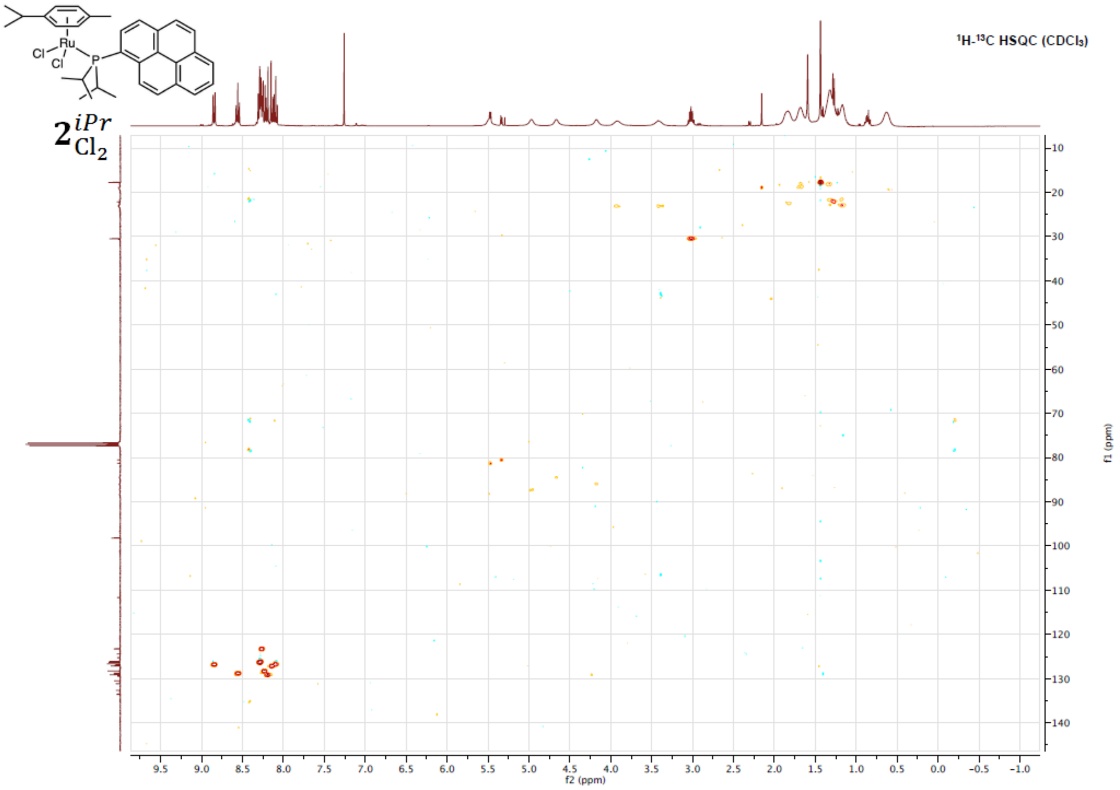

Figure S28. ^{1}H-^{13}C HSQC NMR spectrum of [RuCl$_2$(η^6-*p*-cymene)(diisopropyl(1-pyrenyl)phosphane)] ($\mathbf{2}^{iPr}_{\mathrm{Cl_2}}$).

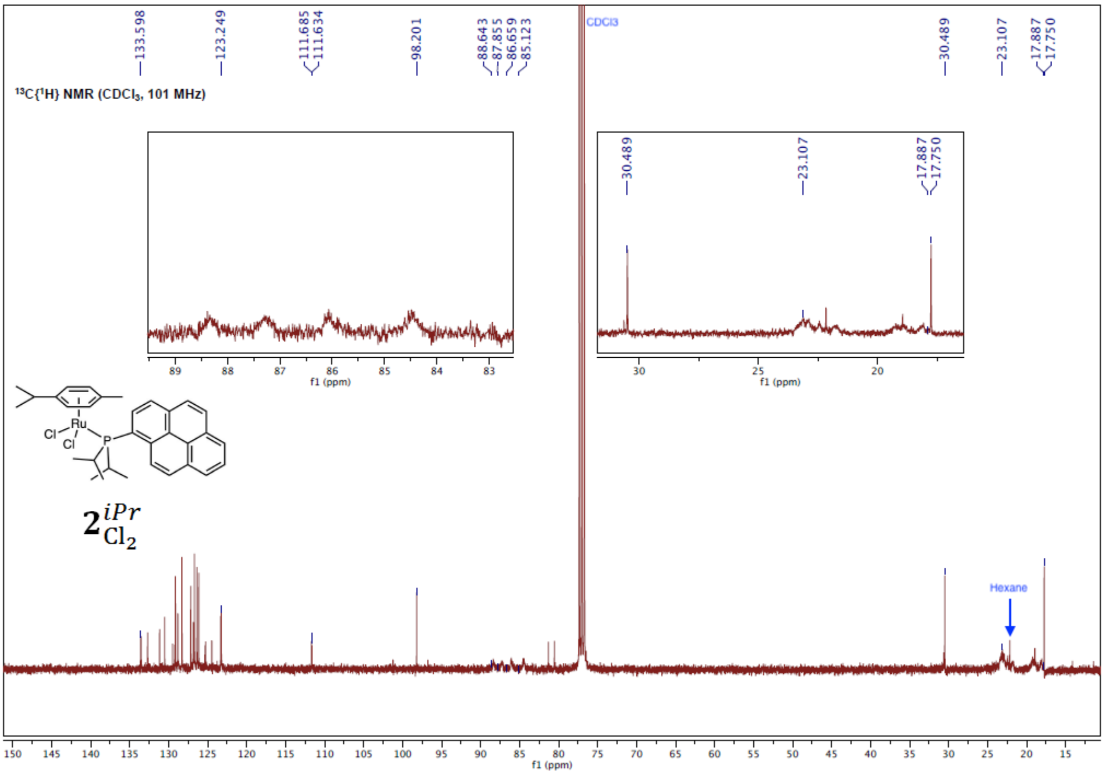

Figure S29. $^{13}C\{^1H\}$ NMR spectrum of [$RuCl_2$(η^6-*p*-cymene)(diisopropyl(1-pyrenyl)phosphane)] ($\mathbf{2}^{iPr}_{Cl_2}$).

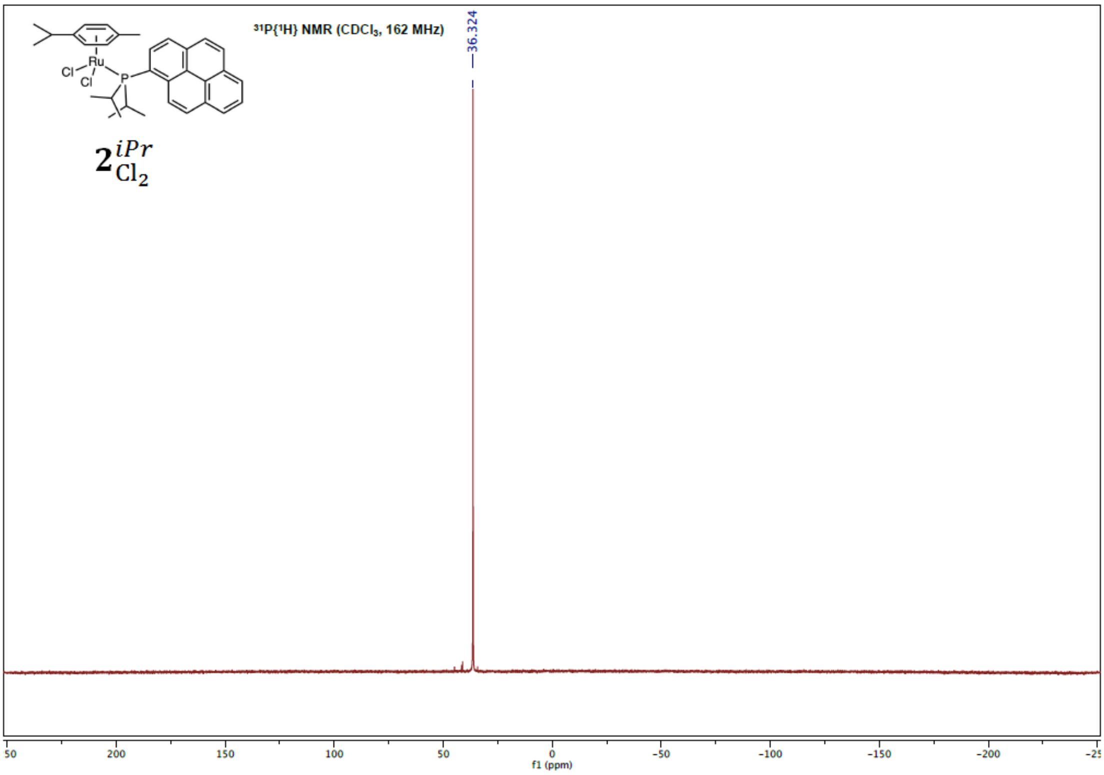

Figure S30. $^{31}P\{^1H\}$ NMR spectrum of [$RuCl_2$(η^6-*p*-cymene)(diisopropyl(1-pyrenyl)phosphane)] ($\mathbf{2}^{iPr}_{Cl_2}$).

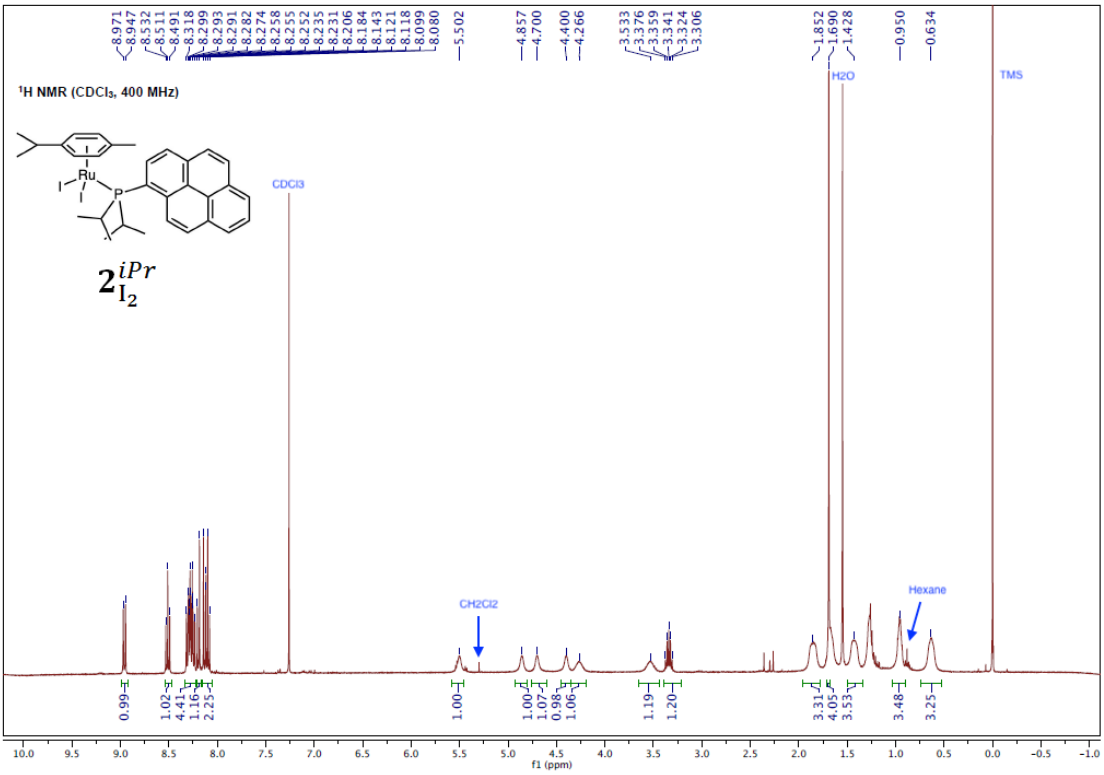

Figure S31. ^{1}H NMR spectrum of [RuI$_2$(η^6-*p*-cymene)(diisopropyl(1-pyrenyl)phosphane)] ($\mathbf{2}_{I_2}^{iPr}$).

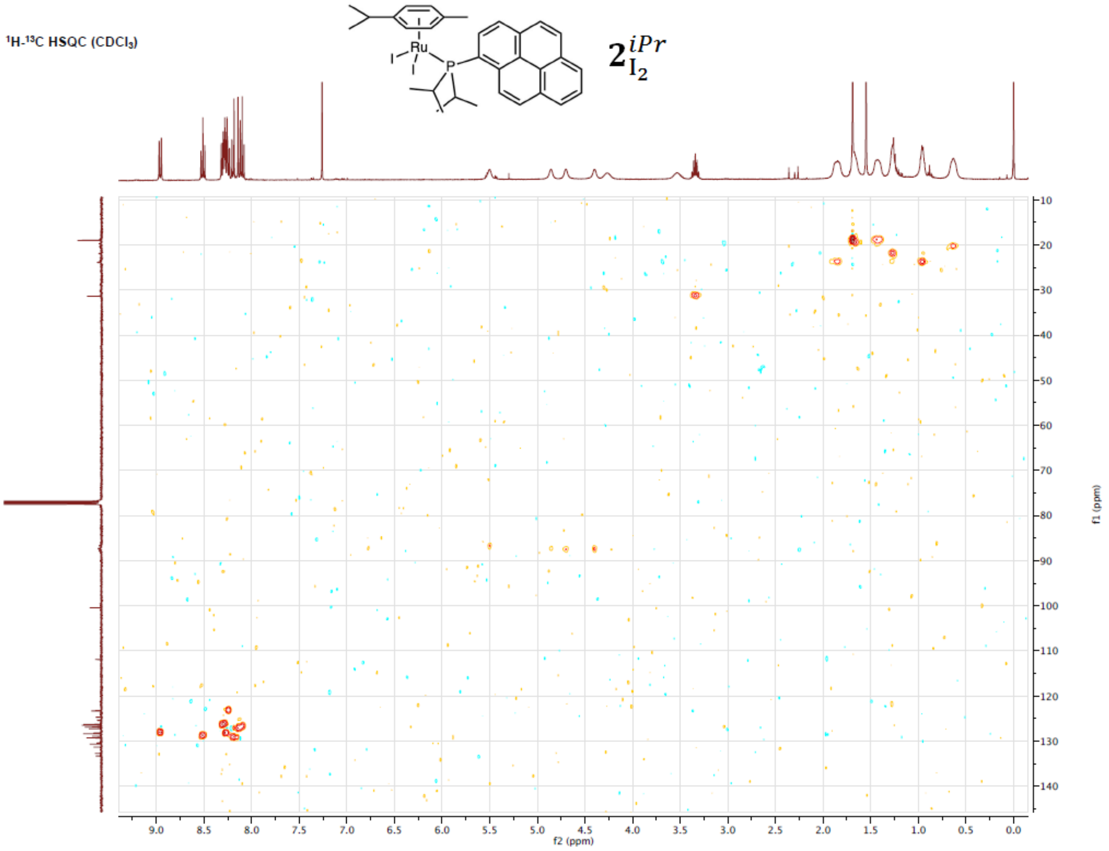

Figure S32. ^{1}H-^{13}C HSQC NMR spectrum of [RuI$_2$(η^6-*p*-cymene)(diisopropyl(1-pyrenyl)phosphane)] ($\mathbf{2}_{I_2}^{iPr}$).

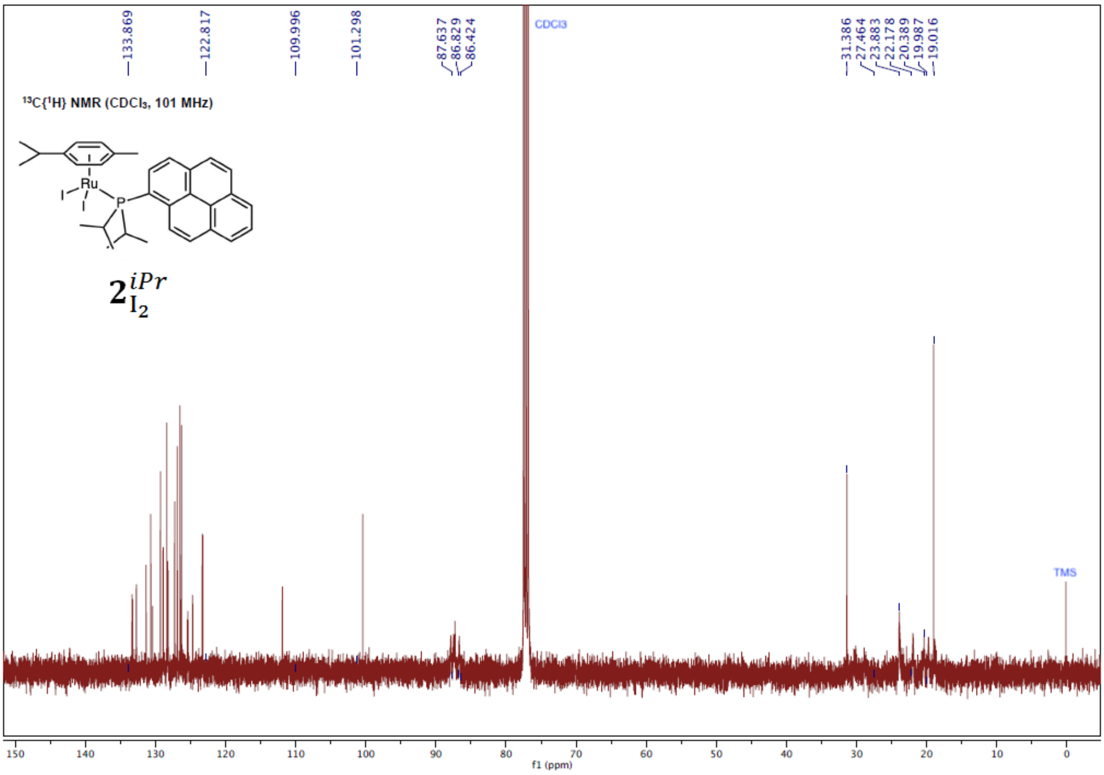

Figure S33. $^{13}C\{^1H\}$ NMR spectrum of [RuI$_2$(η^6-*p*-cymene)(diisopropyl(1-pyrenyl)phosphane)] ($\mathbf{2}^{iPr}_{I_2}$).

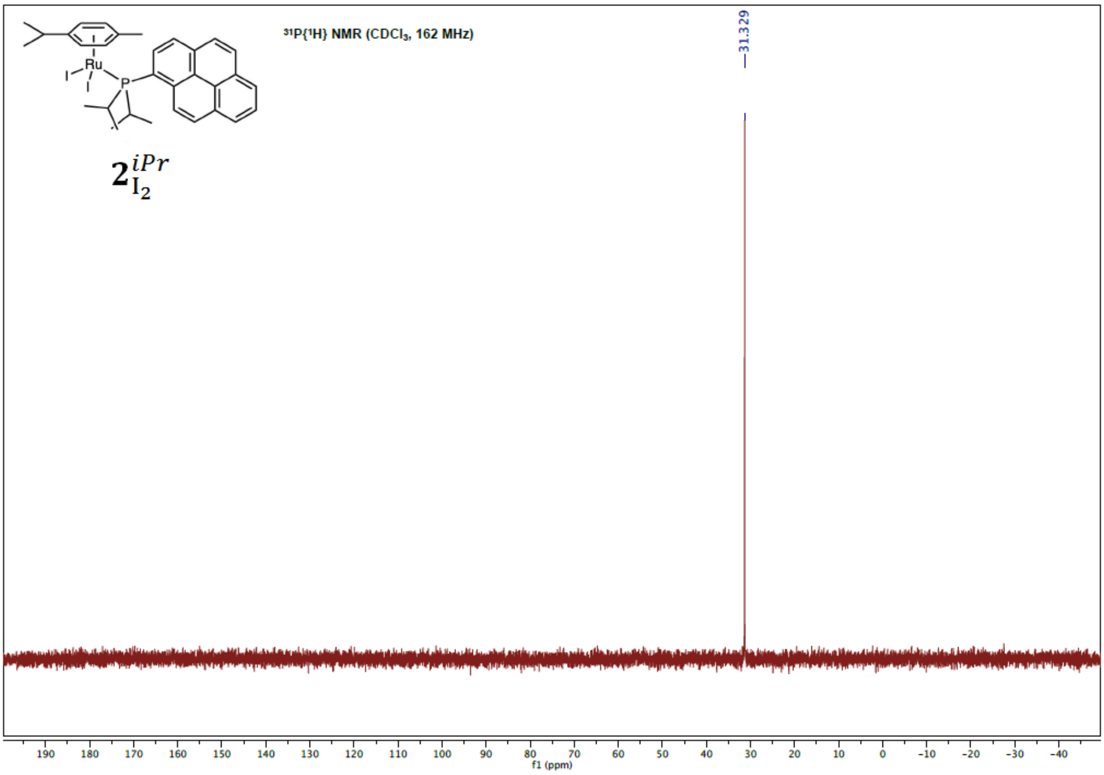

Figure S34. $^{31}P\{^1H\}$ NMR spectrum of [RuI$_2$(η^6-*p*-cymene)(diisopropyl(1-pyrenyl)phosphane)] ($\mathbf{2}^{iPr}_{I_2}$).

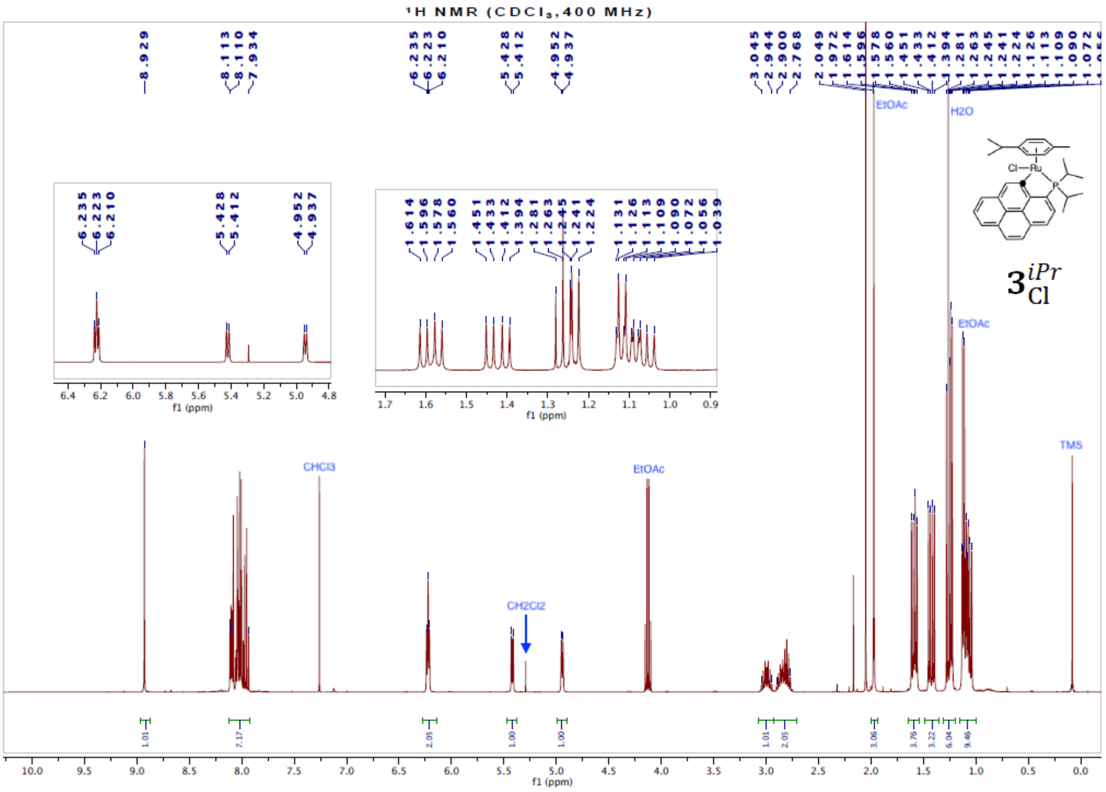

Figure S35. ^{1}H NMR spectrum of [RuCl(η^{6}-*p*-cymene)(k^{2}C-diisopropyl(1-pyrenyl)phosphane)] ($\mathbf{3}_{\mathrm{Cl}}^{iPr}$).

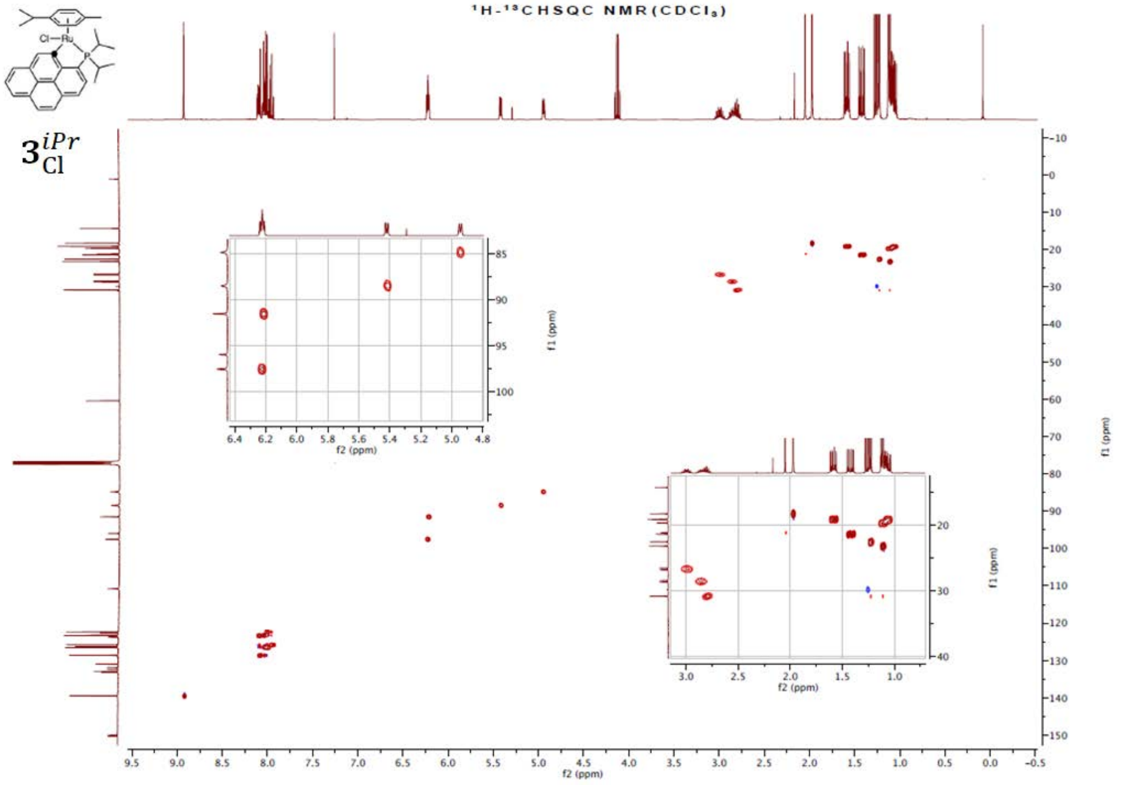

Figure S36. ^{1}H-^{13}C HSQC NMR spectrum of [RuCl(η^{6}-*p*-cymene)(k^{2}C-diisopropyl(1-pyrenyl)phosphane)] ($\mathbf{3}_{\mathrm{Cl}}^{iPr}$).

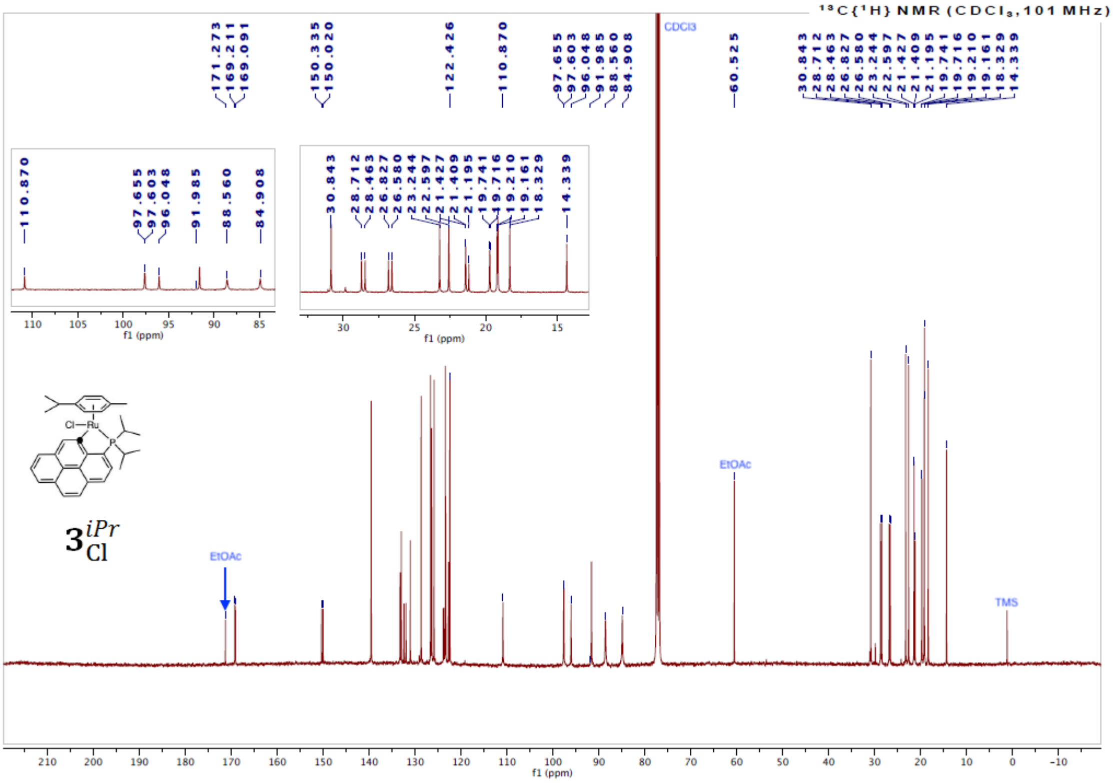

Figure S37. $^{13}C\{^{1}H\}$ NMR spectrum of [RuCl(η^6-*p*-cymene)(k^2C-diisopropyl(1-pyrenyl)phosphane)] (**3**$^{iPr}_{Cl}$).

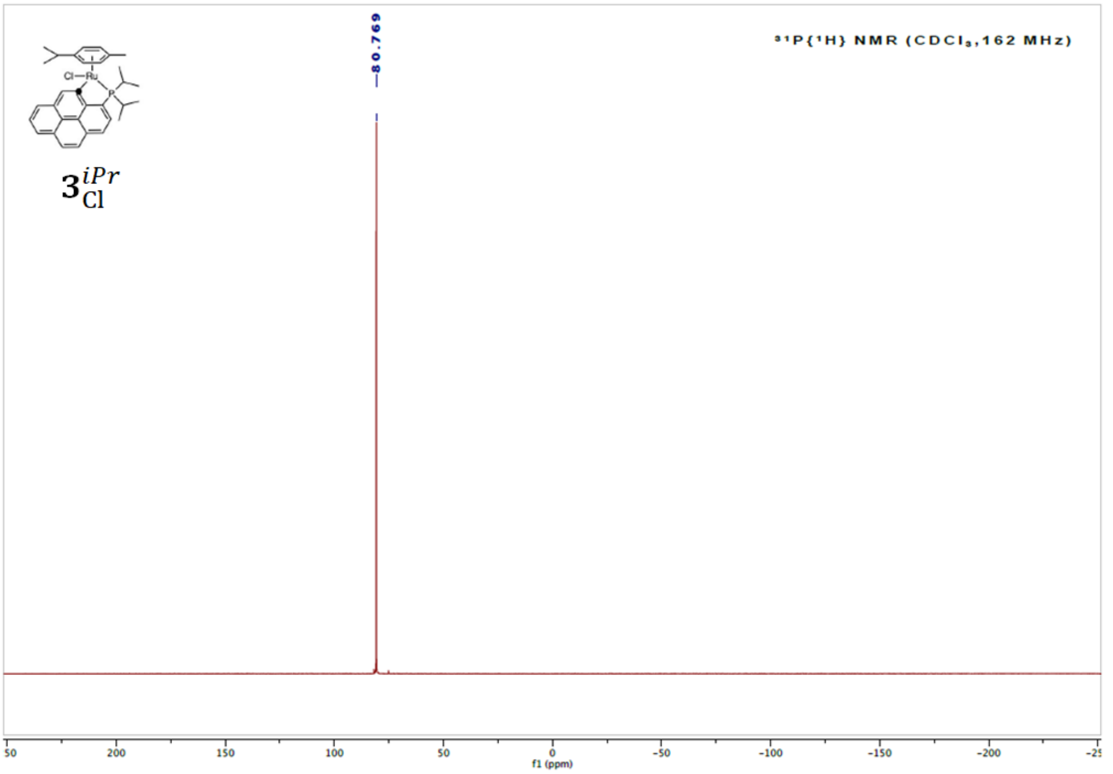

Figure S38. $^{31}P\{^{1}H\}$ NMR spectrum of [RuCl(η^6-*p*-cymene)(k^2C-diisopropyl(1-pyrenyl)phosphane)] (**3**$^{iPr}_{Cl}$).

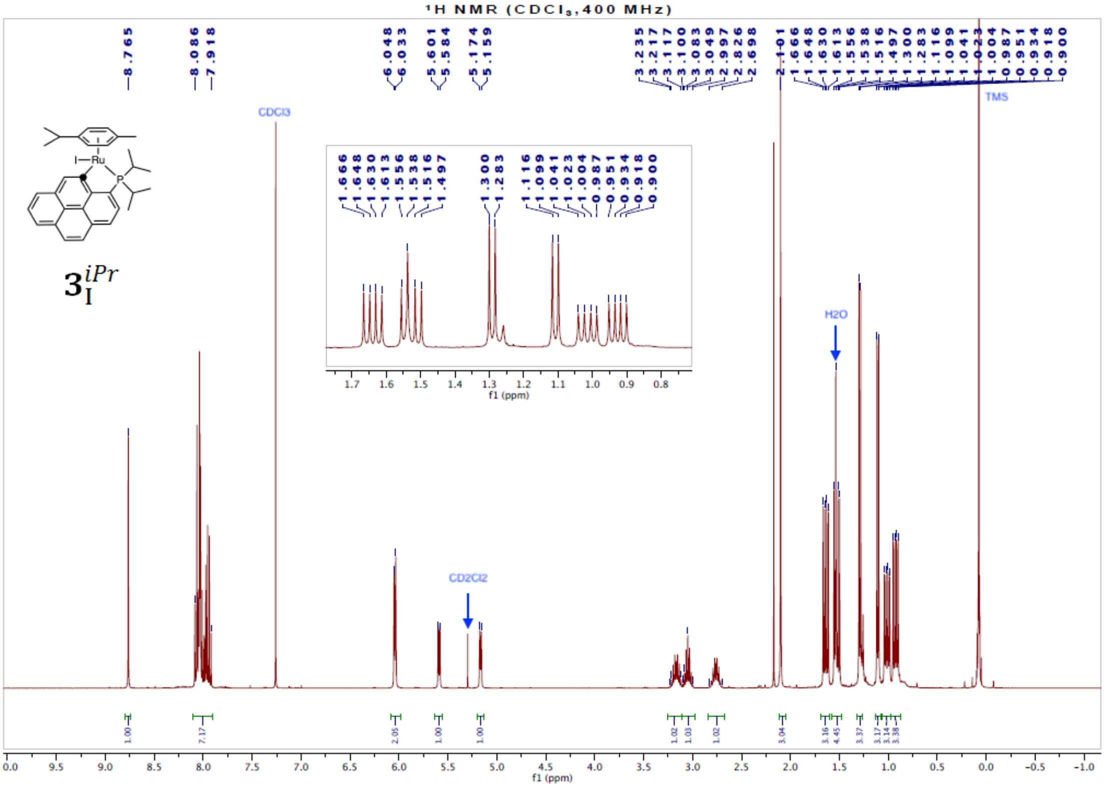

Figure S39. ^{1}H NMR spectrum of [RuI(η^6-*p*-cymene)(k^2C-diisopropyl(1-pyrenyl)phosphane)] ($\mathbf{3}_{\mathrm{I}}^{iPr}$).

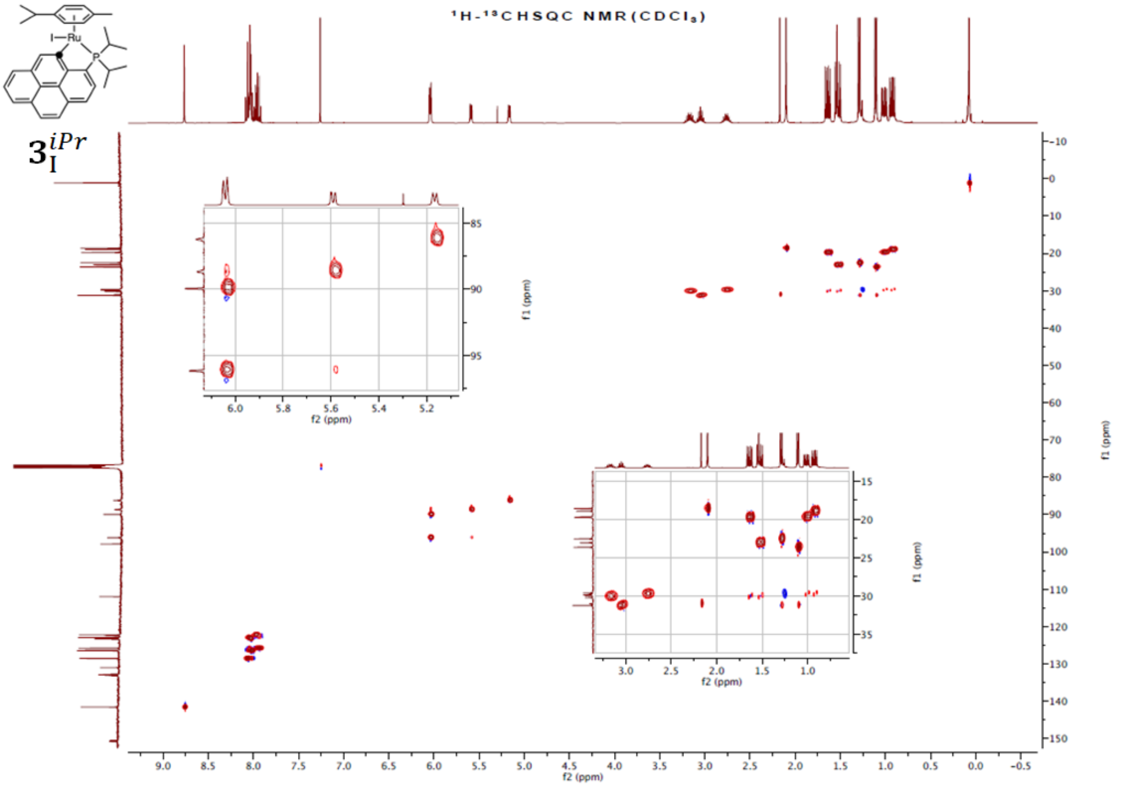

Figure S40. ^{1}H-^{13}C HSQC NMR spectrum of [RuI(η^6-*p*-cymene)(k^2C-diisopropyl(1-pyrenyl)phosphane)] ($\mathbf{3}_{\mathrm{I}}^{iPr}$).

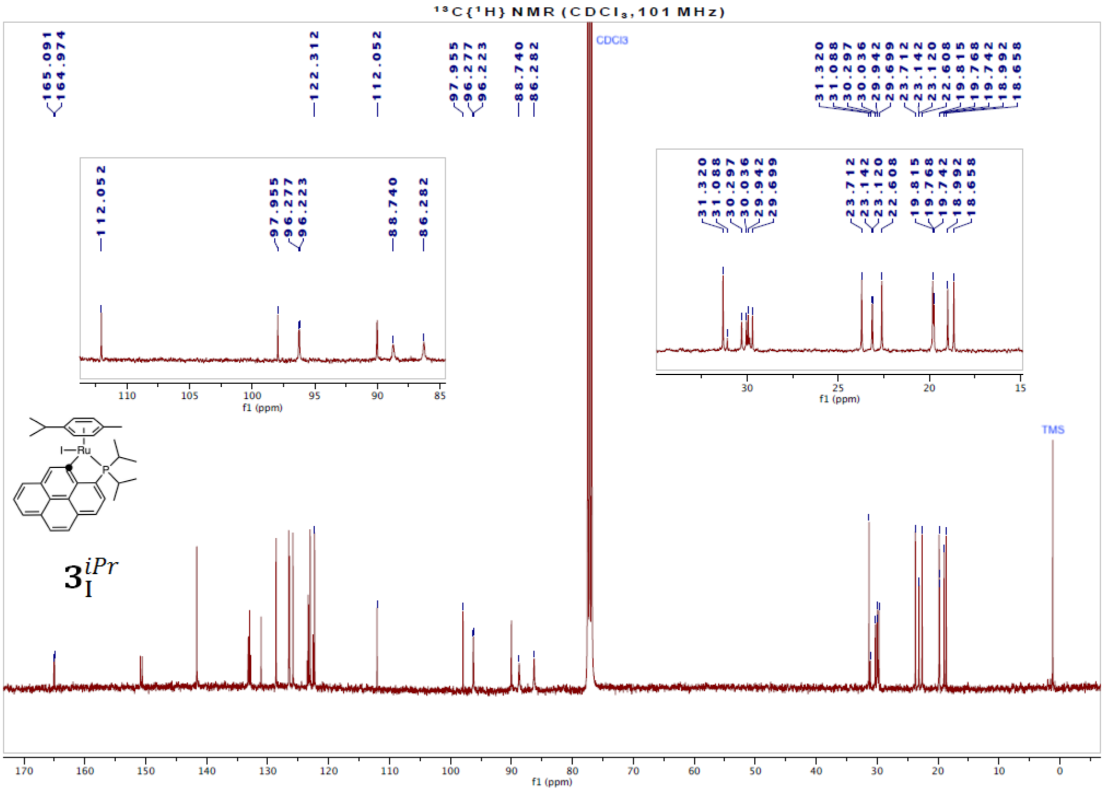

Figure S41. $^{13}C\{^1H\}$ NMR spectrum of [RuI(η^6-*p*-cymene)(k^2C-diisopropyl(1-pyrenyl)phosphane)] ($\mathbf{3}_{\mathrm{I}}^{iPr}$).

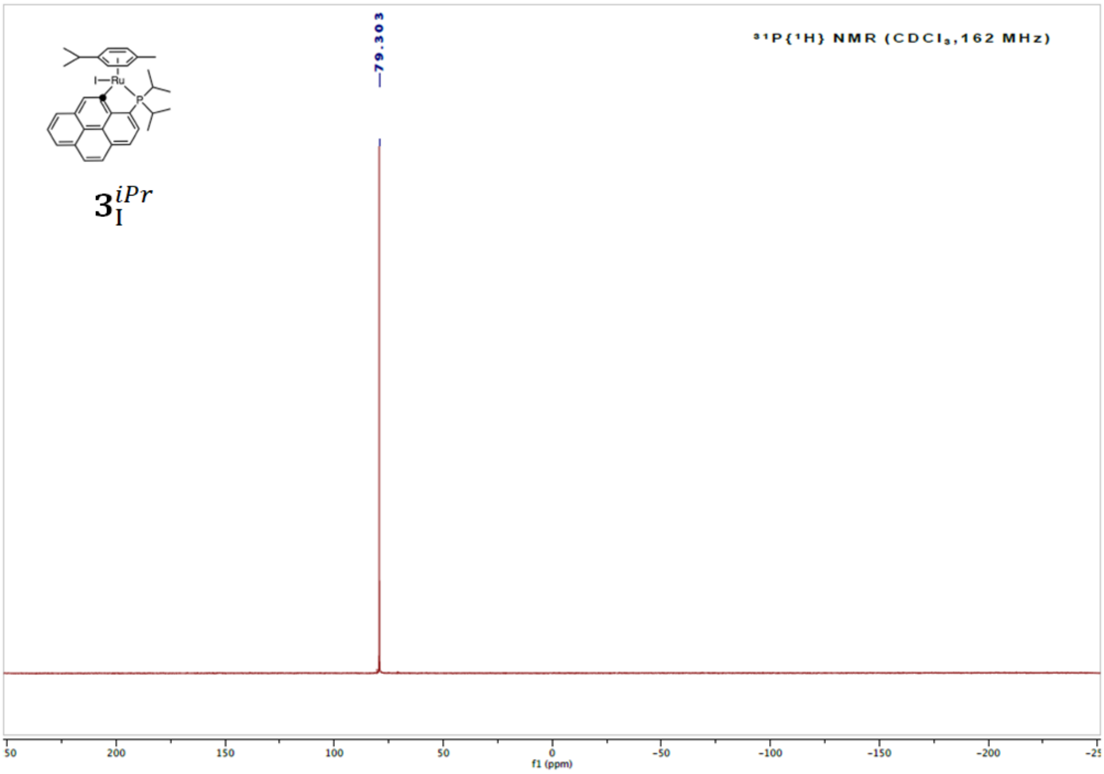

Figure S42. $^{31}P\{^1H\}$ NMR spectrum of [RuI(η^6-*p*-cymene)(k^2C-diisopropyl(1-pyrenyl)phosphane)] ($\mathbf{3}_{\mathrm{I}}^{iPr}$).

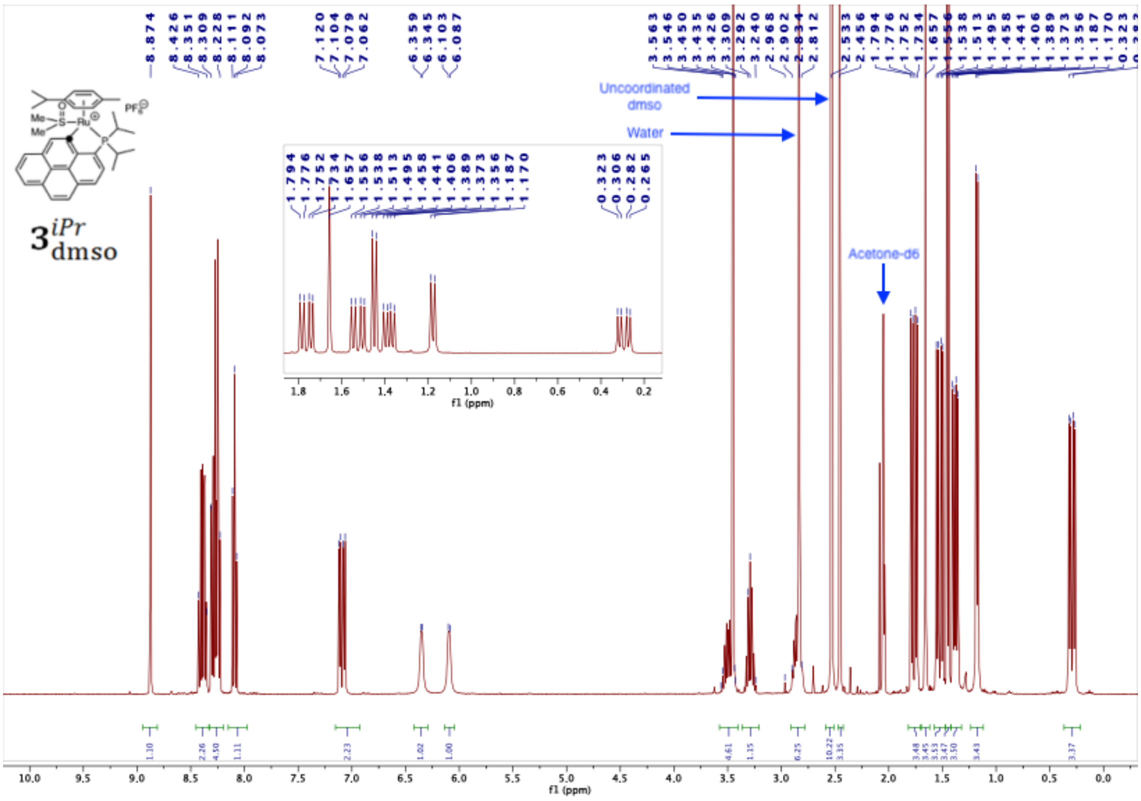

Figure S43. ^1H NMR spectrum of [Ru(η^6-*p*-cymene)(κS-dmso)(k^2C-diisopropyl(1-pyrenyl)phosphane)]PF_6 ($\mathbf{3}^{iPr}_{\mathrm{dmso}}$).

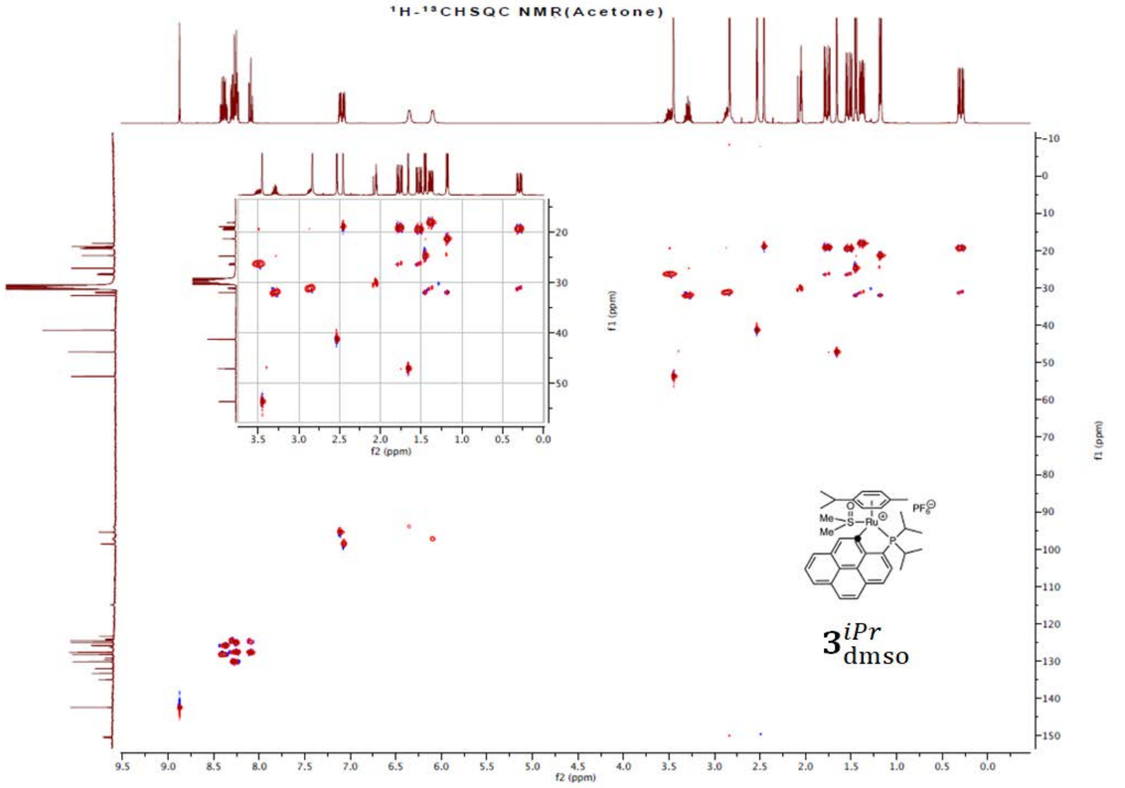

Figure S44. ^1H-^{13}C HSQC NMR spectrum of [Ru(η^6-*p*-cymene)(κS-dmso)(k^2C-diisopropyl(1-pyrenyl)phosphane)]PF_6 ($\mathbf{3}^{iPr}_{\mathrm{dmso}}$).

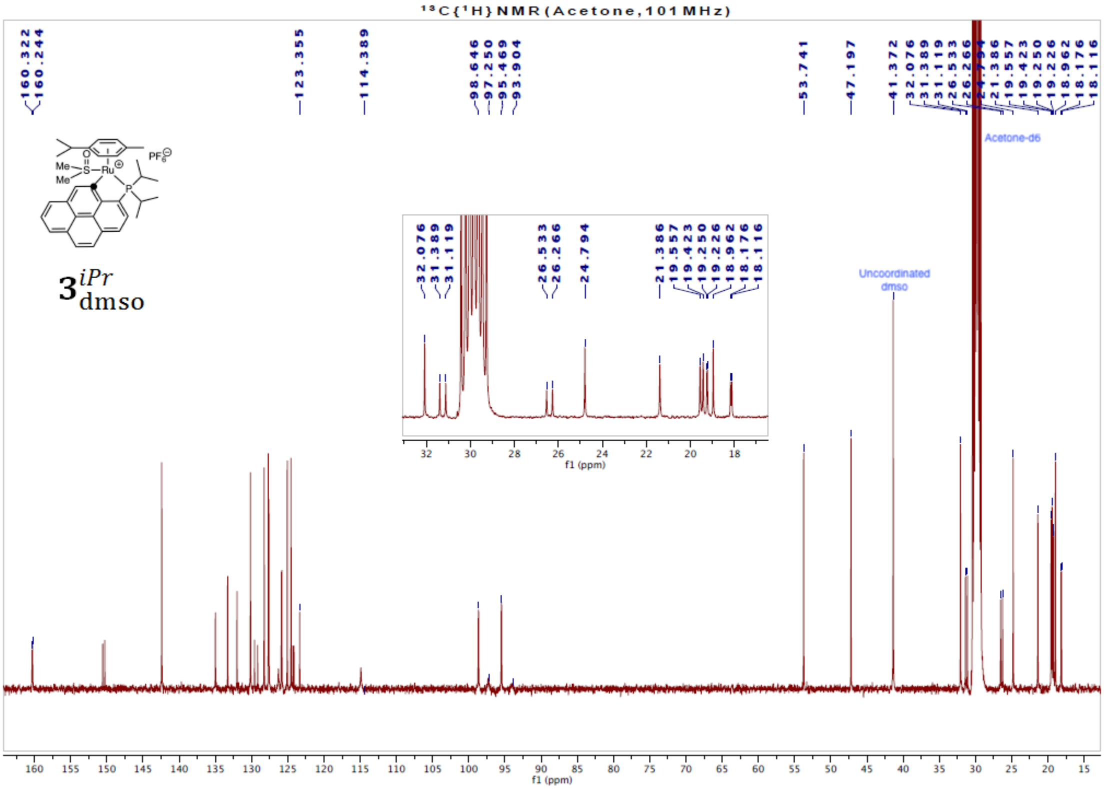

Figure S45. $^{13}C\{^1H\}$ NMR spectrum of [Ru(η^6-*p*-cymene)(κS-dmso)(k^2C-diisopropyl(1-pyrenyl)phosphane)]PF_6 ($\mathbf{3}^{iPr}_{\mathrm{dmso}}$).

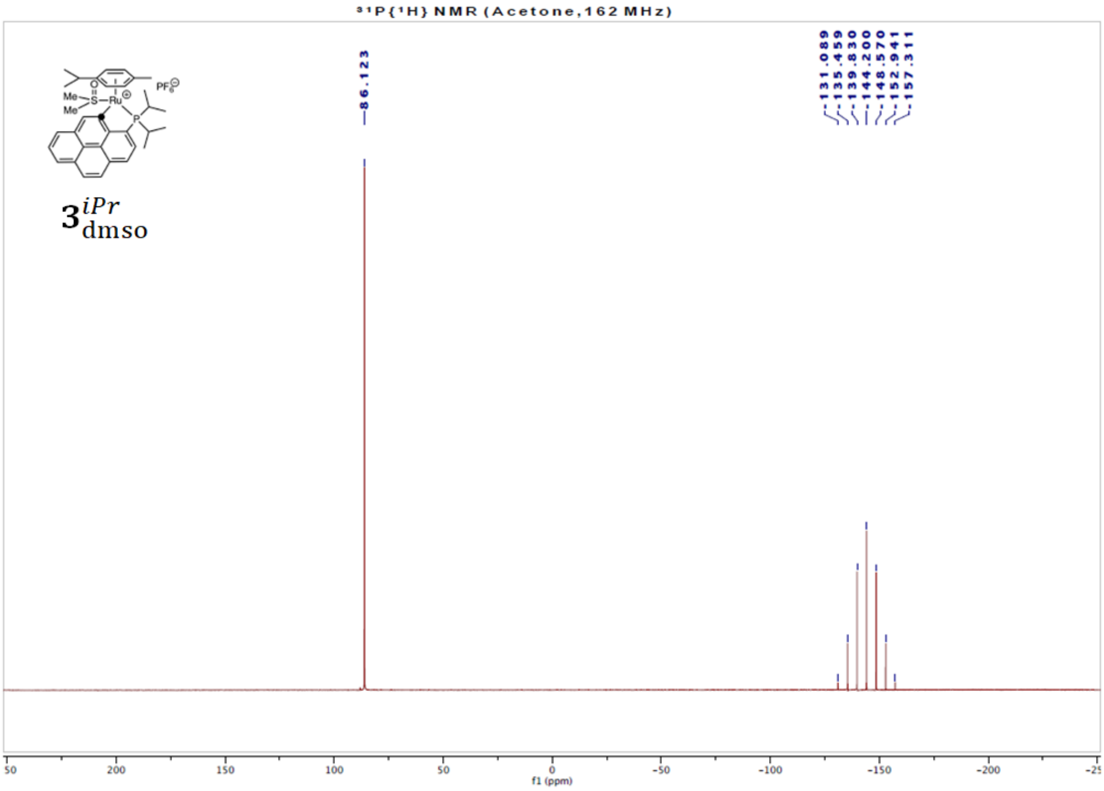

Figure S46. $^{31}P\{^1H\}$ NMR spectrum of [Ru(η^6-*p*-cymene)(κS-dmso)(k^2C-diisopropyl(1-pyrenyl)phosphane)]PF_6 ($\mathbf{3}^{iPr}_{\mathrm{dmso}}$).

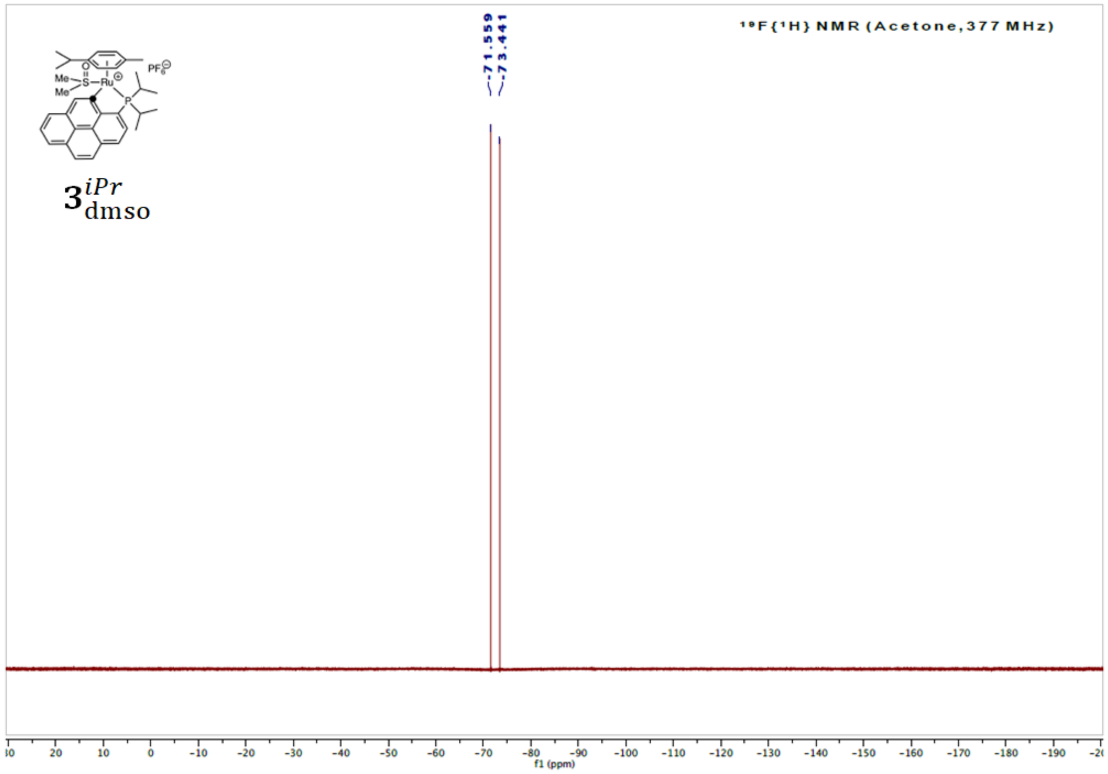

Figure S47. $^{19}F\{^1H\}$ NMR spectrum of [Ru(η^6-*p*-cymene)(κS-dmso)(k^2C-diisopropyl(1-pyrenyl)phosphane)]PF_6 ($\mathbf{3}^{iPr}_{dmso}$).

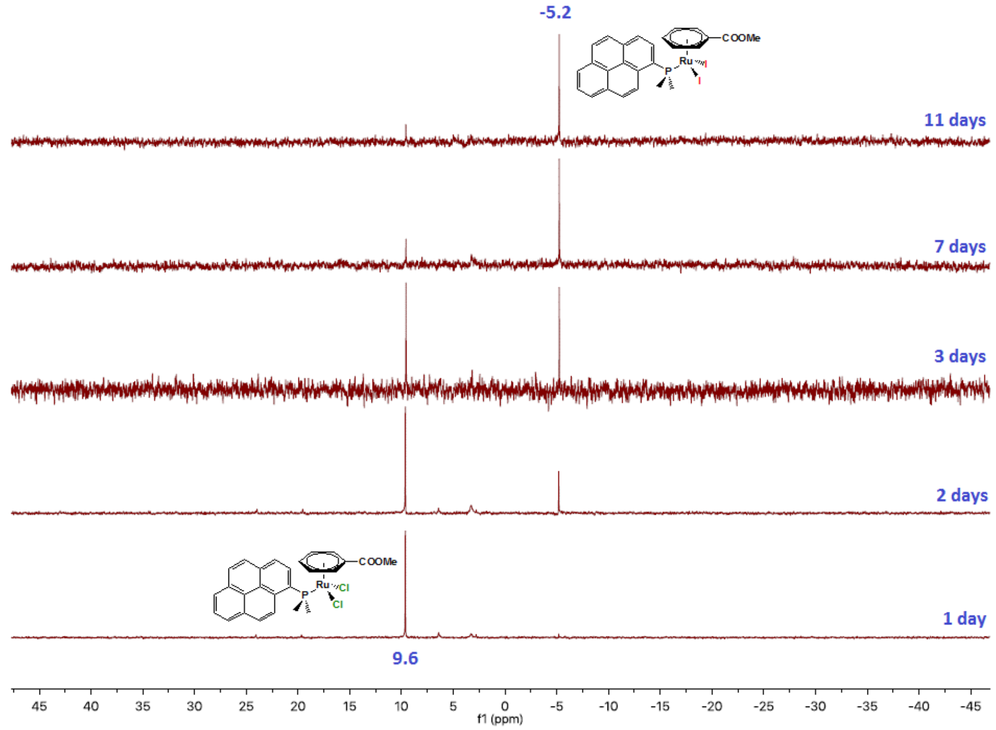

Figure S48. Time-dependent ^{31}P NMR spectra illustrating the progressive conversion of chlorido [$RuCl_2$(η^6-methylbenzoate)(dimethyl(1-pyrenyl)phosphane)] ($\mathbf{1}^{Me}_{Cl_2}$) to iodido complex [RuI_2(η^6-methylbenzoate)(dimethyl(1-pyrenyl)phosphane)] ($\mathbf{1}^{Me}_{I_2}$). The total conversion to $\mathbf{1}^{Me}_{I_2}$ is achieved after more than 11 days under reflux in acetone (see **Experimental Section**).

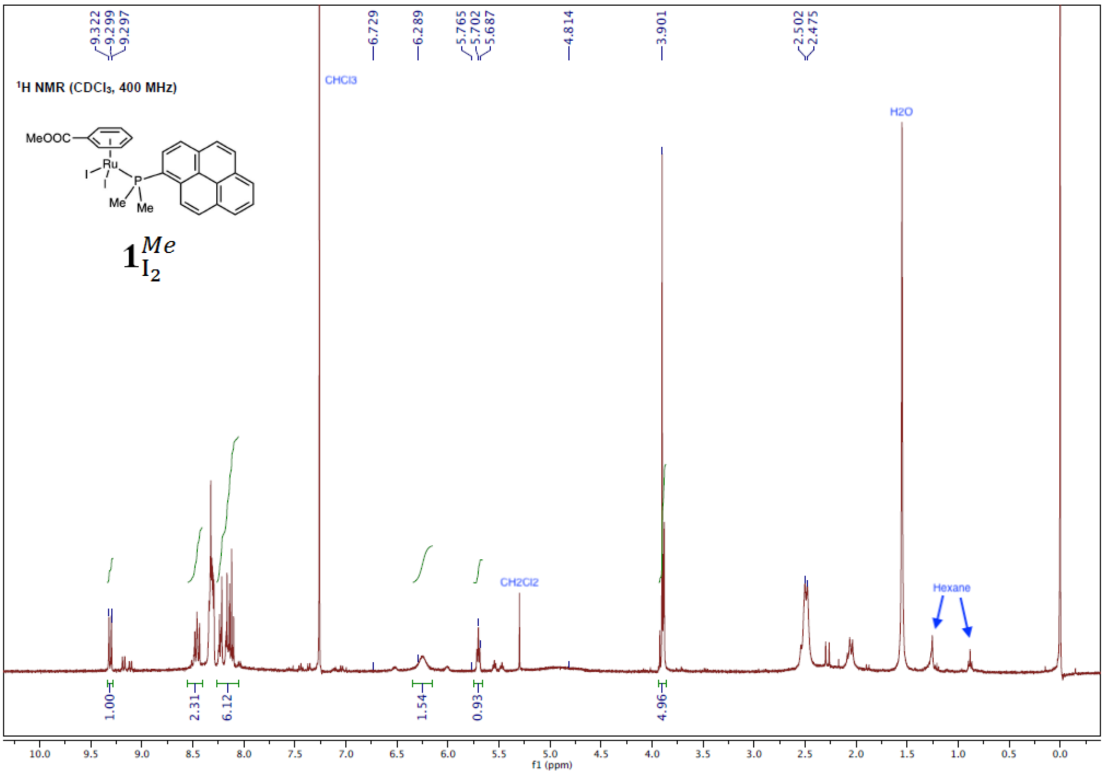

Figure S49. ^{1}H NMR spectrum of [RuI_2(η^6-methylbenzoate)(dimethyl(1-pyrenyl)phosphane)] ($\mathbf{1}^{Me}_{I_2}$).

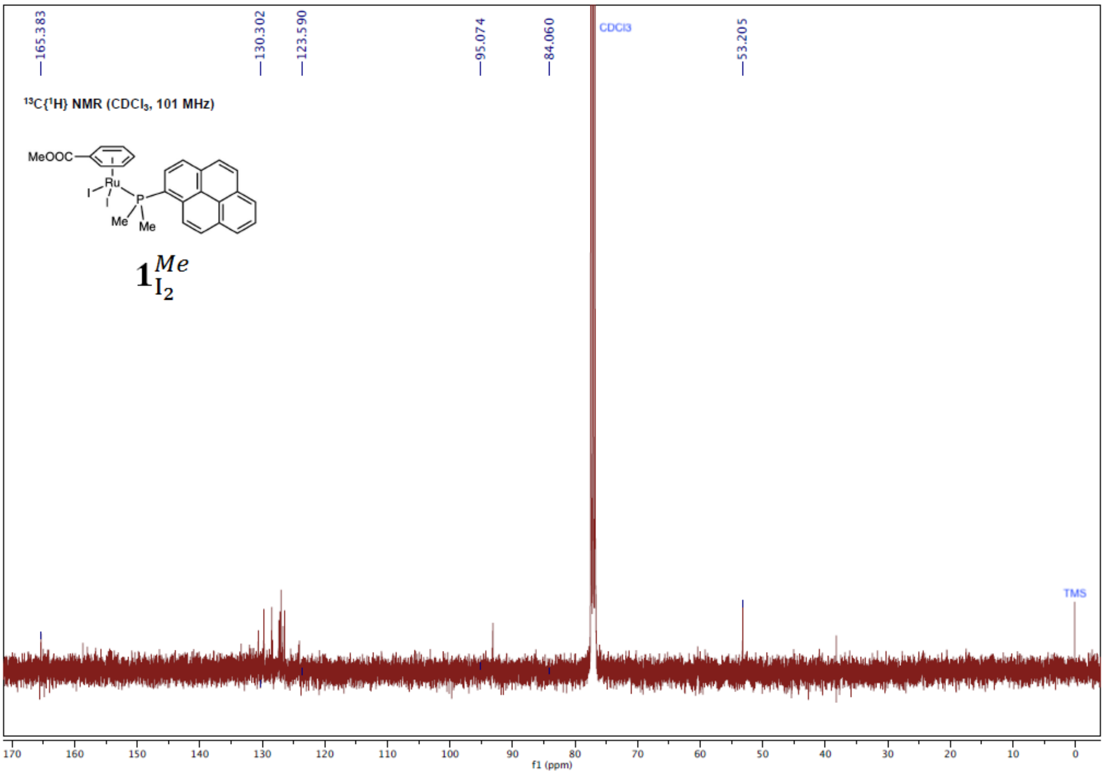

Figure S50. ^{13}C{^{1}H} NMR spectrum of [RuI_2(η^6-methylbenzoate)(dimethyl(1-pyrenyl)phosphane)] ($\mathbf{1}^{Me}_{I_2}$).

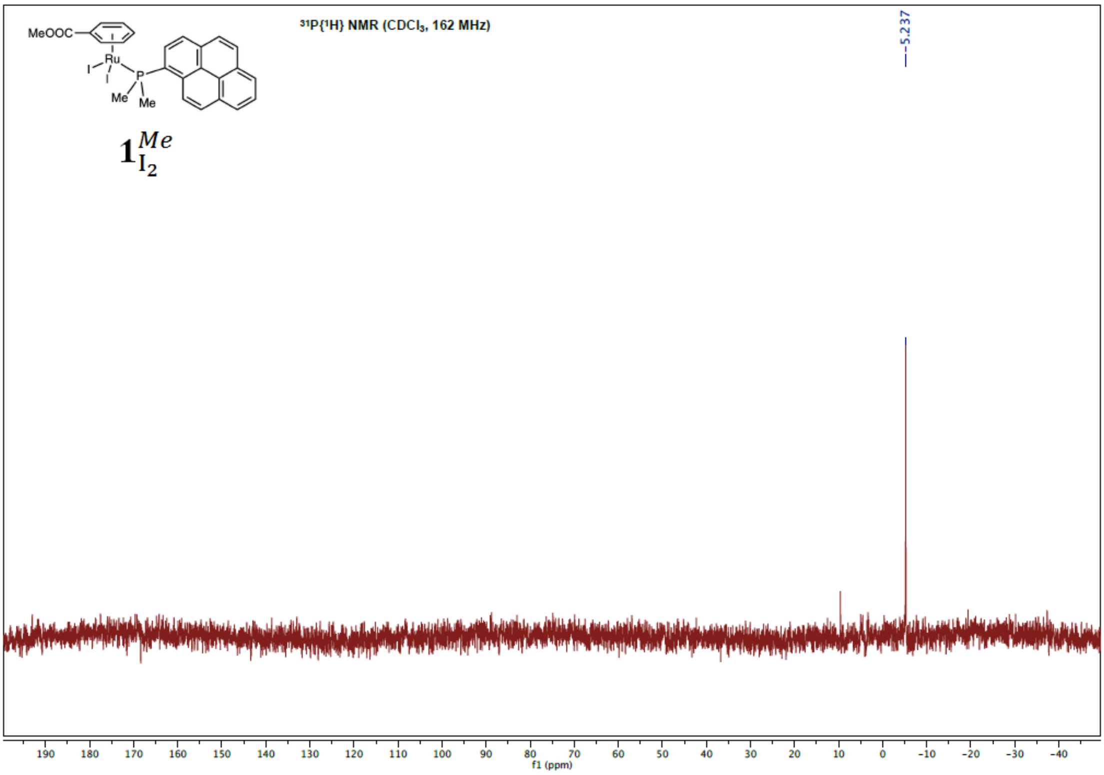

Figure S51. $^{31}P\{^1H\}$ NMR spectrum of [RuI_2(η^6-methylbenzoate)(dimethyl(1-pyrenyl)phosphane)] ($\mathbf{1}^{Me}_{I_2}$).

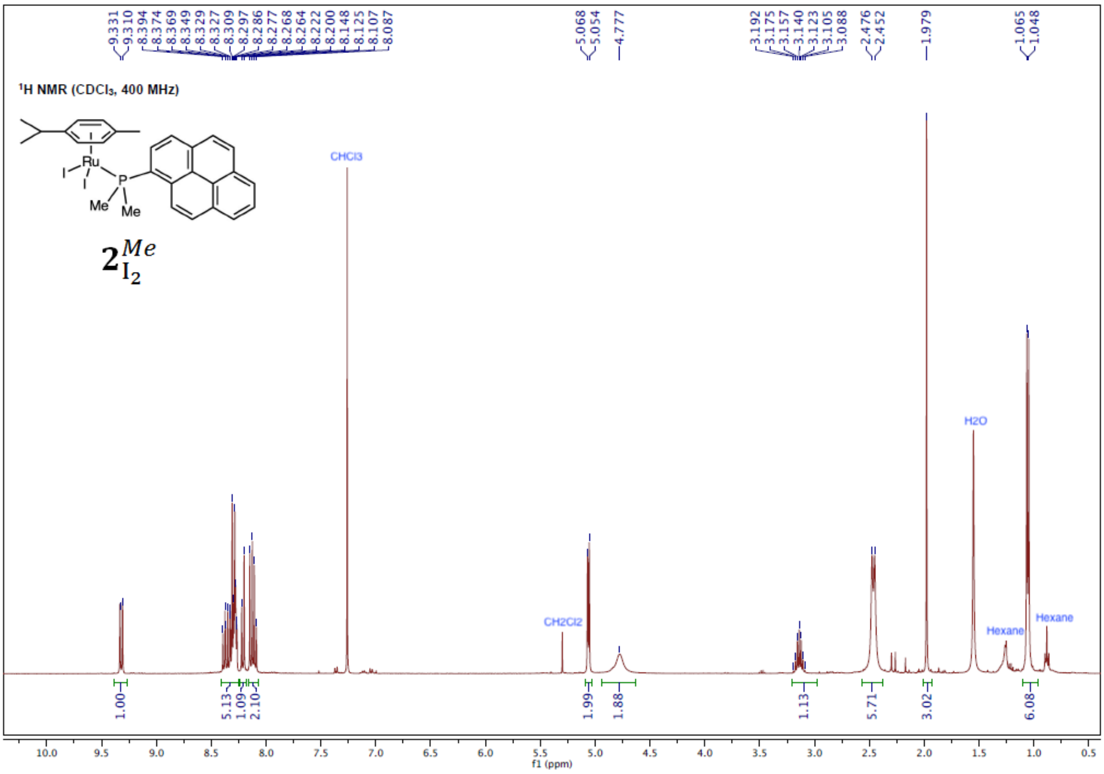

Figure S52. ^1H NMR spectrum of [RuI_2(η^6-*p*-cymene)(dimethyl(1-pyrenyl)phosphane)] ($\mathbf{2}^{Me}_{I_2}$).

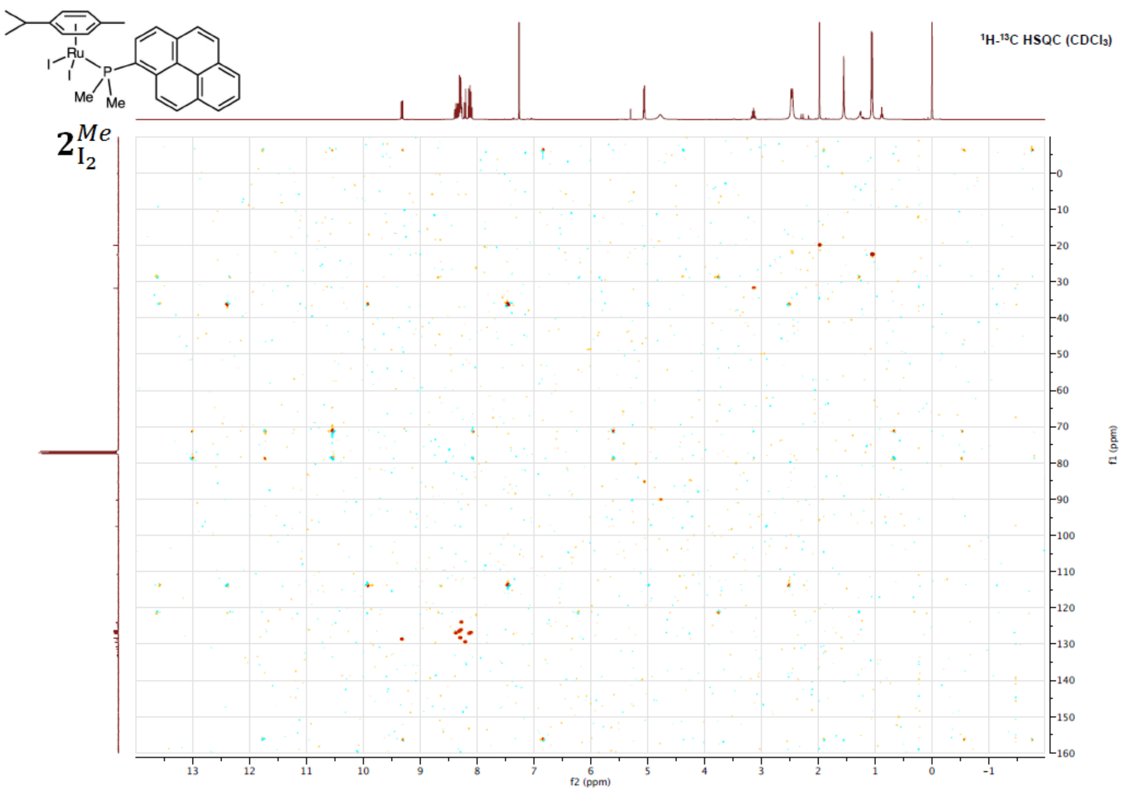

Figure S53. ^{1}H-^{13}C HSQC NMR spectrum of [RuI$_2$(η^6-*p*-cymene)(dimethyl(1-pyrenyl)phosphane)] ($\mathbf{2}^{Me}_{I_2}$)

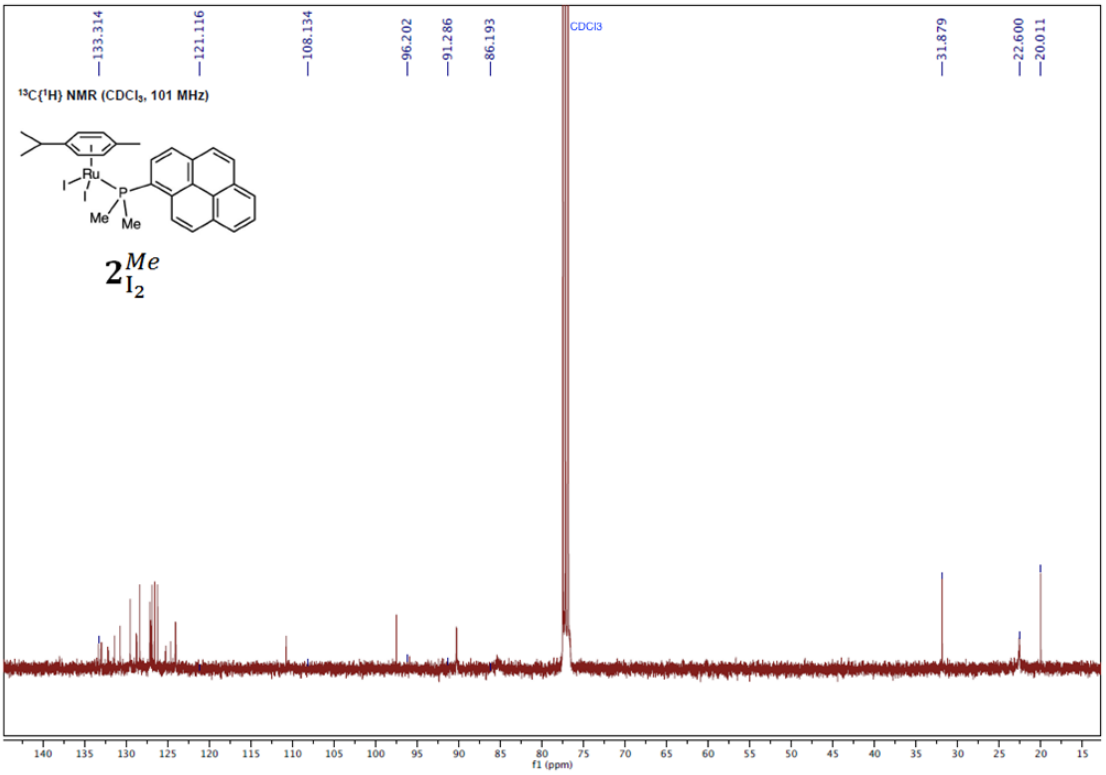

Figure S54. ^{13}C{^{1}H} NMR spectrum of [RuI$_2$(η^6-*p*-cymene)(dimethyl(1-pyrenyl)phosphane)] ($\mathbf{2}^{Me}_{I_2}$).

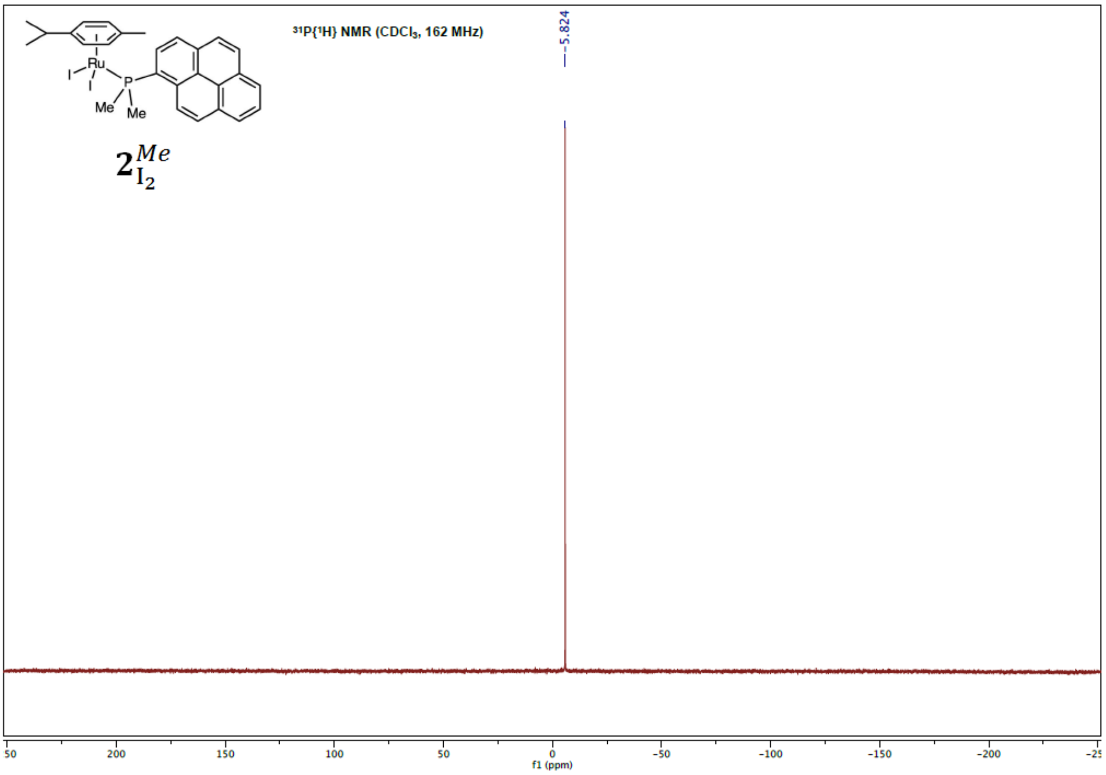

Figure S55. $^{31}P\{^1H\}$ NMR spectrum of [RuI_2(η^6-*p*-cymene)(dimethyl(1-pyrenyl)phosphane)] ($\mathbf{2}_{I_2}^{Me}$).

Cartesian Coordinates

[$RuCl_2$(*p*-cym)PR_3], E = -2596.89005773 au, $\square_1$= 17.1444 cm^{-1}

Ru	-0.638488095730	-0.104215792844	2.094982881275
Cl	-1.104549494155	-2.491296225141	1.747360745745
Cl	0.513552182500	-0.822132980065	4.150449299640
P	1.486872676880	-0.370733629180	0.988495293667
C	2.060066308730	-2.162690990159	0.667584168171
H	1.107045974785	-2.679632150759	0.488581498564
C	2.658167059418	-2.797381886689	1.927414892126
H	2.749990386692	-3.883125138069	1.762199738640
H	2.017056529232	-2.639668748635	2.805845781340
H	3.664752231194	-2.414415734867	2.151867210270
C	2.961159168721	-2.364338063422	-0.553929113659
H	3.137371210795	-3.445373661881	-0.680652179614
H	3.942067387401	-1.882953700534	-0.443523883957
H	2.507550129072	-1.996885825703	-1.483238956988
C	2.925011622310	0.367554407233	1.982076762032
H	2.939983534781	-0.360673602263	2.809257580364
C	2.629138331431	1.721710158203	2.630215934758
H	3.463283564893	1.976355893324	3.303970366658
H	1.716662595858	1.670211542018	3.233798504251
H	2.527725394876	2.545576491274	1.910060061998
C	4.300342067544	0.333828204899	1.303891582602
H	5.046684181966	0.721129394638	2.016668069847
H	4.355596290928	0.956380889791	0.400700176596
H	4.610221322223	-0.682153557468	1.032439410874
C	1.449350227854	0.442762582486	-0.684790742591
C	2.099962453832	1.673489054949	-0.866155908202

H	2.642854036913	2.130607320342	-0.042286900793
C	2.075415471510	2.350020340751	-2.083307963959
H	2.599324382642	3.303551714959	-2.184473554059
C	1.396593373785	1.816092761369	-3.190646462415
C	1.380351209982	2.475795782629	-4.464797244898
H	1.921310188453	3.420087972540	-4.564653865373
C	0.710458879246	1.942255411641	-5.530073710666
H	0.706924453646	2.452707140376	-6.496406957675
C	-0.006303537296	0.704645423036	-5.409088133556
C	-0.705250461368	0.138960451744	-6.492892632906
H	-0.697899857373	0.657274820442	-7.454873952452
C	-1.401660410483	-1.063037817736	-6.346791878062
H	-1.938559709276	-1.484624905287	-7.199272667977
C	-1.419006022113	-1.727534813380	-5.119584717702
H	-1.969777740471	-2.664477860275	-5.006039288646
C	-0.732053507990	-1.200539072019	-4.008273548255
C	-0.747854760576	-1.835989371089	-2.725789683797
H	-1.334728601897	-2.749667055827	-2.602773300742
C	-0.078333659692	-1.305804750833	-1.658197902741
H	-0.174621022013	-1.794200610408	-0.688105961605
C	0.700612564690	-0.103604578887	-1.767343922749
C	0.694242569067	0.578275685213	-3.031568300490
C	-0.010096521094	0.026958474036	-4.146178614669
C	-0.731523644412	2.362969008987	4.473257403477
H	-1.486584315006	3.059328617885	4.874136040264
H	0.195781651031	2.925291507833	4.305354684554
H	-0.548054020088	1.590474473500	5.232873508656
C	-1.250779245515	1.757750685078	3.206995160173
C	-0.725572705280	2.118761386232	1.938122565312
H	0.107721258349	2.818582195947	1.877484229450
C	-2.334562511121	0.809322922422	3.254306360413

H	-2.709306181337	0.474485070075	4.222560456148
C	-1.248415086982	1.526754668815	0.748162898574
H	-0.799931637719	1.778922050902	-0.213673130991
C	-2.854639258938	0.253252227664	2.070353075294
H	-3.625655686505	-0.513889522937	2.143524105906
C	-2.339062863638	0.614252891624	0.778170515692
C	-2.984053732500	0.181533452701	-0.522561670039
H	-2.180035995491	0.134096126473	-1.272663999876
C	-3.964290901882	1.286540085029	-0.962961584522
H	-4.408264554166	1.027898197791	-1.936584559371
H	-3.459009549264	2.258723845906	-1.065390196141
H	-4.782997607031	1.400886219172	-0.234552353857
C	-3.681865437938	-1.178649197801	-0.475424484895
H	-3.009901924485	-1.962483531921	-0.100101090490
H	-4.017635368985	-1.457254799029	-1.485676866571
H	-4.573768243468	-1.154933976801	0.170701924606

$[RuCl(DMSO)(\textit{p}\text{-cym})PR_3]^+$, E = -2689.57907932 au, $\square_1$= 18.9427 cm^{-1}

Ru	-0.5662339351	-0.1390197286	2.0607682074
P	1.6147487828	-0.3674431906	0.9722861242
C	2.1844640806	-2.1176042219	0.4555752253
H	1.2436794418	-2.6016668435	0.162406823
C	2.7450127736	-2.9291738426	1.6254160401
H	2.8728951958	-3.9708445378	1.2889962698
H	2.07330711	-2.9232467398	2.4913302457
H	3.731243348	-2.5683690278	1.9521627975
C	3.131343439	-2.1387929903	-0.7505047223
H	3.3566561759	-3.1921604052	-0.984396236
H	4.086491502	-1.6342457003	-0.554135821
H	2.6894863578	-1.6917801751	-1.6497073034

C	3.0223386367	0.2814949165	2.0560053432
H	2.955405662	-0.4651675272	2.8666070704
C	2.7362521995	1.6496850885	2.6803442
H	3.4009350538	1.7953975882	3.5459231722
H	1.7008664152	1.7293877957	3.0281291227
H	2.9191185155	2.481399556	1.986061988
C	4.4379323178	0.2129805254	1.4700668266
H	5.1422088361	0.5876733396	2.2306062601
H	4.5619074277	0.8351456559	0.5736536418
H	4.7439237861	-0.809436654	1.2213130965
C	1.5995331207	0.5583368659	-0.6439520802
C	2.3532790973	1.7347840711	-0.7748760437
H	2.9271156207	2.1141710979	0.0664779463
C	2.395342766	2.4559282789	-1.9651268633
H	2.9998003591	3.3638328268	-2.0274520049
C	1.6709674282	2.0315474213	-3.090479266
C	1.7048300603	2.750654049	-4.3316296822
H	2.327789199	3.6463841848	-4.3928668212
C	0.9781243212	2.3336577817	-5.411356313
H	1.011508592	2.8893215198	-6.3517913588
C	0.150287298	1.1635905746	-5.3388289571
C	-0.6166856486	0.7245825829	-6.4354202281
H	-0.5756123313	1.2897573152	-7.3697139976
C	-1.4213254522	-0.4127402789	-6.3372514528
H	-2.0095266888	-0.7357676883	-7.1988013082
C	-1.4811115221	-1.1376616573	-5.1462223599
H	-2.1137104098	-2.0253392946	-5.0701610101
C	-0.7289205337	-0.7373128609	-4.0248698388
C	-0.7743061766	-1.4439801858	-2.7812550298
H	-1.4285084463	-2.3155281623	-2.6983047939
C	-0.0372220235	-1.0397213759	-1.7034760363

H	-0.1482119093	-1.5852950679	-0.7663934052
C	0.8240872779	0.1099763043	-1.7535210502
C	0.8672287064	0.8510673774	-2.9833192261
C	0.1002489415	0.4251459681	-4.1117957872
C	-0.4379954584	3.2005507468	3.2890408807
H	-1.1622413715	4.0326459668	3.3068405716
H	0.4775372667	3.5685291599	2.8103336791
H	-0.2258973577	2.9321233558	4.3311311502
C	-1.0549917851	2.0754256015	2.5193118836
C	-0.7876900656	1.8794051615	1.1392315703
H	-0.0031345259	2.4656375087	0.6592253599
C	-2.0325480888	1.2109018637	3.1096275956
H	-2.2179054813	1.2821740375	4.1826358484
C	-1.5584291193	0.96886619	0.355772699
H	-1.3492356788	0.8778994446	-0.7069583981
C	-2.7754237835	0.2757826286	2.3516509921
H	-3.4915222159	-0.3675013477	2.8595792518
C	-2.5738735442	0.1676094133	0.9383224113
C	-3.3728609212	-0.7756250656	0.060926731
H	-2.6339625652	-1.4254669087	-0.4367478138
C	-4.1004512534	0.0293662211	-1.0326370264
H	-4.6446446948	-0.6605232042	-1.694412353
H	-3.4034410472	0.610106612	-1.6537891982
H	-4.8304693509	0.7258259213	-0.5914748218
C	-4.3507320059	-1.6644231562	0.8294815009
H	-3.835708913	-2.2716035392	1.5871008374
H	-4.8473697614	-2.3514993734	0.1290186625
H	-5.134534518	-1.0708692354	1.3260680033
Cl	-0.9137203422	-2.5631829549	1.6322544277
S	0.2127614543	-0.8993902141	4.1518999953
C	-1.1640025286	-1.7893210753	4.9549180936

H -2.0242160718 -1.1134916503 5.0389760948

H -1.4033722654 -2.6445108669 4.3135765662

H -0.8095947677 -2.1110053327 5.9428956961

C 0.4442054783 0.4130543399 5.3998622208

H 1.2516807272 1.0675774772 5.056151455

H -0.4879348871 0.9693781273 5.5455614818

H 0.7347865098 -0.1063463185 6.3224464481

O 1.4519501646 -1.7867050636 4.2576481001

[RuCCl(*p*-cym)], E = -2136.22968643 au, □$_1$= 12.8155 cm^{-1}

Ru -1.4046106805 -0.2119970992 -0.4323990271

P -0.1706099016 -0.7037412376 1.4903324196

C -3.7231379963 -0.3324138331 -0.7733408058

C -3.0515773481 0.0549126887 -1.9944980257

H -3.3326816981 0.9932741509 -2.4754949639

C -2.0227144656 -0.7218984326 -2.5515209406

H -1.5323770839 -0.3914115408 -3.4682858137

C -1.5345771694 -1.8913171663 -1.8729538685

C -2.1929565333 -2.2532709642 -0.6589539876

H -1.8647438171 -3.146981571 -0.1323249666

C -3.3135218249 -1.5202261962 -0.1464038453

H -3.7891090657 -1.850764689 0.775569167

C -4.8359755963 0.545122316 -0.2418948805

H -4.5005004184 1.5842695406 -0.4002177152

C -6.1117748067 0.3222332162 -1.0747137442

H -6.9042931796 1.0130515777 -0.7488124504

H -6.4842280323 -0.7074124478 -0.9533972157

H -5.9314409302 0.4953088964 -2.1464998846

C -5.1062217674 0.3618991997 1.2523768304

H -5.8401430056 1.1069064239 1.5939483135

H	-4.1875020649	0.4939936764	1.8434417607
H	-5.5208881488	-0.6336132982	1.4754865477
C	-0.4286148481	-2.725194364	-2.4474375563
H	0.1032680409	-3.2704350584	-1.65538451
H	0.2952671832	-2.102604274	-2.9918952971
H	-0.8363514047	-3.4648685022	-3.15624221
C	0.4822206382	0.3203010144	-1.0894470178
C	0.667575596	1.0182551009	-2.3042757158
H	-0.204837004	1.3587834378	-2.8661293685
C	1.9308053655	1.2953792914	-2.8333844441
H	2.0143045969	1.8278393415	-3.7857071293
C	2.9729433689	0.2415985192	-0.9067779569
C	4.1353771512	-0.1435716324	-0.1734730106
C	6.5716577306	-0.2879968331	0.0251533656
H	7.5665377785	-0.099511073	-0.3863320941
C	6.4318617479	-0.9094036437	1.2691093074
H	7.3230718291	-1.2053416051	1.827362695
C	5.167024916	-1.1526079982	1.8084660548
H	5.0656764598	-1.6337691273	2.7844955774
C	4.0034399452	-0.779197388	1.1051481435
C	2.6870448267	-0.9912288784	1.6352948553
H	2.5955527333	-1.437019972	2.6293446622
C	1.5762589766	-0.6288181429	0.9171267815
C	1.6710260585	-0.0256828939	-0.3792912604
C	-0.2397254601	0.5978918243	2.8883811462
C	-0.2815072217	-2.3590725583	2.3939789286
H	0.3641880624	-2.1874424631	3.2700867896
C	3.1080834218	0.9034572859	-2.167078728
C	5.4394047975	0.1071834463	-0.7152605624
C	5.5414695835	0.7601150391	-1.9911201337
C	4.4234090946	1.1452084012	-2.6830525828

H 4.5190268814 1.6493728333 -3.6486839659

H 6.5380624597 0.9489488769 -2.3984058021

C 0.3150753533 -3.5407203049 1.6244298487

H 0.3128232376 -4.428911204 2.2772019408

H -0.2571638688 -3.7977375379 0.7236103585

H 1.3538881795 -3.3545714297 1.3203637873

C -1.7016832085 -2.6496359362 2.8921055239

H -2.3436778243 -3.0205029739 2.0817995354

H -1.6681388459 -3.4315021243 3.6674454728

H -2.1908189722 -1.7667024239 3.3292332177

H -1.2806781563 0.9524385731 2.8304710349

C 0.0425911742 0.0732004805 4.2979987094

H -0.0294857467 0.9159129166 5.0055574798

H -0.6708835313 -0.6926351556 4.6302055587

H 1.0613749845 -0.3356907639 4.385845095

C 0.6920870177 1.7738873818 2.5727214022

H 0.4523559754 2.6147421356 3.2438964939

H 1.7439432891 1.4977353785 2.7411358645

H 0.5815678188 2.1295491798 1.5411328476

Cl -1.7201189065 2.1504806639 0.217673194

[RuC(*p*-cym)(DMSO)]$^{+}$, E = -2228.95855568au, $\square_1$= 18.2253 cm^{-1}

Ru -1.3840726365 -0.0890930469 -0.5521893854

P -0.1957785866 -0.5309124913 1.4368243697

C -3.7664911798 -0.2656358539 -0.4312050302

C -3.321898254 0.2567967504 -1.6902808362

H -3.708326054 1.223793924 -2.0211136954

C -2.4431613597 -0.4535710006 -2.544510196

H -2.1851449721 -0.0393865578 -3.5190647377

C -1.9069086268 -1.7125423043 -2.1325434157

C	-2.2414072484	-2.1745583895	-0.8360003029
H	-1.8118608686	-3.1125048432	-0.4878300054
C	-3.1577056509	-1.4612949134	0.0026595199
H	-3.41165594	-1.8775860418	0.9764184731
C	-4.9072608014	0.4016271307	0.3095454176
H	-4.9301480905	1.4545504371	-0.0125765036
C	-6.2236968805	-0.2536507362	-0.1536444865
H	-7.0827209841	0.2633893649	0.3000951285
H	-6.2589566524	-1.3116303082	0.1503812167
H	-6.3354127631	-0.2083849705	-1.2474113439
C	-4.7837772867	0.3644146123	1.8326392549
H	-5.6242537316	0.908837046	2.2880675663
H	-3.8517625305	0.835431903	2.1727849791
H	-4.8110868998	-0.6652457671	2.2207585967
C	-1.0490454368	-2.5367172205	-3.040063432
H	-0.3457767522	-3.15869625	-2.4701590558
H	-0.4883716525	-1.9150091974	-3.7490827523
H	-1.6979689825	-3.2088866347	-3.6256253292
C	0.5576512907	0.0210563286	-1.3138461153
C	0.7968313247	0.2283190374	-2.6884235131
H	-0.0434493552	0.3254538649	-3.3764512238
C	2.0803227562	0.349573687	-3.2238655255
H	2.2048058989	0.5140871906	-4.2980331371
C	3.0281844088	0.082939147	-1.00166084
C	4.1608699944	0.0070528349	-0.1372737273
C	6.5846301835	0.0566326488	0.2069959322
H	7.5947667341	0.1584277566	-0.1976584067
C	6.3939590042	-0.1536367242	1.575404448
H	7.2601590124	-0.2154554197	2.2380122275
C	5.1089082072	-0.2860814382	2.1042678685
H	4.9655713864	-0.451974468	3.1748753609

C	3.9777571945	-0.2060871035	1.2663907284
C	2.6446204706	-0.345390084	1.7721098578
H	2.5259400091	-0.5537534914	2.8363557597
C	1.5571714829	-0.2454854028	0.9419581126
C	1.7036056429	-0.0441734468	-0.4704219266
C	-0.6137187291	0.5912410851	2.9235665165
C	-0.0485571464	-2.3000582036	2.1212264673
H	0.521505114	-2.1174008702	3.0502939916
C	3.2208474207	0.2807761024	-2.4047585839
C	5.4854797006	0.1383824401	-0.6707933479
C	5.642315167	0.3442117338	-2.0835765986
C	4.5554991643	0.4116865099	-2.9130048517
H	4.6883869598	0.5676242023	-3.9869575133
H	6.6539258455	0.4447112068	-2.4849119359
C	0.7912649387	-3.1911554884	1.1973695565
H	0.8353609006	-4.2054344748	1.6251226232
H	0.3409879376	-3.2745754756	0.1968179085
H	1.8209087236	-2.8317464725	1.079721469
C	-1.3555885244	-3.0184844093	2.4789859586
H	-1.8211076619	-3.4602791772	1.5885895196
H	-1.1186447749	-3.8529260231	3.1581852184
H	-2.0985344239	-2.3889281277	2.9779376563
H	-1.1140120817	1.4346504263	2.4289944581
C	-1.6047788725	-0.0470075276	3.8996441499
H	-1.9158221515	0.7180563872	4.6287514776
H	-2.5122037077	-0.4169485834	3.4068694411
H	-1.1474200714	-0.8742443423	4.4620212422
C	0.5908296948	1.1548266674	3.677537747
H	0.2220381353	1.8820487779	4.4194334468
H	1.1352494505	0.3739398612	4.2289381034
H	1.2903503297	1.676699404	3.0134178286

S -1.028486234 2.1781100696 -0.2765955245

C -0.9009101137 3.0215715806 -1.8908020504

H 0.042710553 2.7011809959 -2.3453429385

H -1.7528328698 2.7294107373 -2.5172110221

H -0.8979094649 4.1019198178 -1.6962372122

C -2.5083703987 3.0526055369 0.3435516446

H -3.3197579663 2.9546690004 -0.3872767954

H -2.7869268993 2.6045079654 1.3034277354

H -2.2204965556 4.1043188781 0.4712833274

O 0.1501195879 2.6618159808 0.5685058626

[RuI_2(*p*-cym)PR_3], E = -1699.70901319 au, $\square_1$= 14.7539 cm^{-1}

Ru -0.6157355306 -0.1292294302 2.1700644773

P 1.5076767912 -0.4077306474 1.0039475034

C 2.126198125 -2.1864201967 0.6633466559

H 1.1865033059 -2.7282192483 0.4856127536

C 2.7576301619 -2.8227932594 1.9048754366

H 2.8992671011 -3.8984697736 1.7122615771

H 2.1190951054 -2.7233211444 2.7933304213

H 3.7452743765 -2.3984830084 2.1373447245

C 3.0153109802 -2.3617192218 -0.5719295119

H 3.1814941132 -3.4407058135 -0.725962951

H 4.0008886696 -1.8919656286 -0.4572398755

H 2.5578653198 -1.9679745361 -1.4881687486

C 2.9665577168 0.3700423117 1.9459845122

H 3.0188417566 -0.3454115169 2.7835048554

C 2.6846105787 1.7352907071 2.5796130075

H 3.5168860174 1.9807775722 3.2587431291

H 1.7645654524 1.7207109493 3.175943703

H	2.6107081712	2.5515008816	1.8477417905
C	4.3229894183	0.3419473426	1.2299646124
H	5.0805419648	0.7677121252	1.9080603435
H	4.3397810659	0.9348989744	0.3055576257
H	4.6469915709	-0.6757457811	0.9841733851
C	1.4267087313	0.3959719076	-0.6759949236
C	2.0425512183	1.6441013004	-0.861879093
H	2.5817602813	2.1140130075	-0.0434278913
C	1.9824665142	2.3267953476	-2.0737586881
H	2.480467544	3.2939417476	-2.1763584646
C	1.2958756426	1.7848899888	-3.1720397103
C	1.2334775118	2.457919507	-4.4376097529
H	1.7482733256	3.4165487147	-4.5389101302
C	0.5508178003	1.9201155581	-5.4924038715
H	0.5099453252	2.4415240804	-6.4519822259
C	-0.1310295	0.6633974118	-5.3685275233
C	-0.8452437091	0.0948235514	-6.4407552219
H	-0.8780081898	0.626653896	-7.3948145868
C	-1.5061097456	-1.1267845248	-6.293223323
H	-2.0562857741	-1.5498403375	-7.1364356003
C	-1.4706414727	-1.8092818281	-5.0763240933
H	-1.9926194875	-2.7624309289	-4.9618417623
C	-0.7667831454	-1.2799855563	-3.9768409376
C	-0.7271949685	-1.9355953915	-2.7052088643
H	-1.2792794153	-2.8704412934	-2.5813002061
C	-0.0487205198	-1.3986414017	-1.64726048
H	-0.10098073	-1.9062938342	-0.6849120845
C	0.6841684839	-0.1679060022	-1.7535536535
C	0.6313056619	0.5269726522	-3.0103867132
C	-0.0840763828	-0.0303472674	-4.1150242513
C	-0.9944899117	2.3592743479	4.5914521741

H -1.5969055978 3.2774990614 4.7018538577

H 0.0642246491 2.6497409649 4.6099988857

H -1.2068302297 1.7085562912 5.4483437563

C -1.3677918115 1.7145704706 3.2941006166

C -0.7258959292 2.0992351846 2.0853256759

H 0.0935650366 2.8166785885 2.1111583032

C -2.4142590101 0.7360709239 3.2290771613

H -2.8647722463 0.3700394648 4.1521861131

C -1.144725732 1.5412787411 0.8425950519

H -0.6294304541 1.8352882119 -0.0724661653

C -2.8257066648 0.2056745994 1.985909207

H -3.5936727202 -0.5677551722 1.9740731478

C -2.239226741 0.634526692 0.7502170873

C -2.8172528718 0.3031875103 -0.6099188252

H -1.973220738 0.2543261831 -1.3138826195

C -3.7060881586 1.4883062003 -1.0406039739

H -4.1037832813 1.3092021042 -2.0513078516

H -3.1406547079 2.4321057448 -1.0585400955

H -4.5590489377 1.6118584754 -0.3545221204

C -3.5958042784 -1.0093288887 -0.6862734132

H -2.9928717762 -1.8646328598 -0.3524973886

H -3.9060299896 -1.1932404907 -1.7257757998

H -4.5099059172 -0.975514555 -0.0726157355

I -1.2657405414 -2.9068943111 1.9516687721

I 0.6611372192 -0.8349546157 4.6360506345

[RuI(DMSO)(*p*-cym)PR_3]$^+$, E = -2241.01344299 au, $\square_1$= 18.2956 cm^{-1}

Ru -0.5945417767 -0.1973635118 2.1813445416

P 1.5364814595 -0.4207900557 0.9968113442

C 2.2055883962 -2.1592958437 0.5453232703

H	1.282855359	-2.7237071879	0.3502990459
C	2.9001594211	-2.8531297363	1.7196489242
H	3.0646609229	-3.9075390482	1.4443314586
H	2.297046625	-2.8285449841	2.6353301928
H	3.886632822	-2.4193138895	1.9390909487
C	3.0741670634	-2.2075371437	-0.7178158938
H	3.339142712	-3.2614585883	-0.9024333811
H	4.0117294785	-1.6463809465	-0.6116359923
H	2.5573074274	-1.8401377819	-1.6121646622
C	2.9629047701	0.3597495892	1.9784598718
H	3.0098705518	-0.3685701308	2.8073554819
C	2.6613633274	1.7327998205	2.5912508954
H	3.3980176152	1.9325024589	3.3851956147
H	1.6622349838	1.7888678297	3.0374324543
H	2.7499761863	2.5492875485	1.8620179137
C	4.3396830868	0.3574589808	1.3001962456
H	5.0721278666	0.7726705617	2.0112515008
H	4.3689457172	0.9800585436	0.3960566841
H	4.6831442332	-0.6475324093	1.0323797488
C	1.4242039492	0.4400564319	-0.6512567793
C	2.049157007	1.6856140661	-0.8129898138
H	2.590575918	2.1378315168	0.0130379244
C	1.9994386309	2.3870821551	-2.0147025718
H	2.5037322814	3.3525621756	-2.0992985089
C	1.3203902356	1.8636286032	-3.1261687839
C	1.2751357533	2.5534736346	-4.3833708263
H	1.7921981389	3.5126537223	-4.4652711168
C	0.607982358	2.0287386582	-5.4544045431
H	0.5813395418	2.5618875674	-6.4079856234
C	-0.0743518222	0.7699872989	-5.3566639596
C	-0.7678363392	0.212466036	-6.4479317402

H	-0.7842245153	0.7550835163	-7.3962823979
C	-1.4283828406	-1.0122918692	-6.3266381507
H	-1.9616102816	-1.4268169259	-7.1847830561
C	-1.4136166284	-1.7086339765	-5.1174013827
H	-1.9354487	-2.6640174336	-5.0232269507
C	-0.7307421553	-1.1905110249	-3.999386943
C	-0.7123686986	-1.8603696509	-2.7351006715
H	-1.2667429864	-2.7962607321	-2.6306143894
C	-0.050837593	-1.3356238068	-1.6600191473
H	-0.1177363872	-1.8564228332	-0.7053534236
C	0.6865083368	-0.1065355065	-1.7418543539
C	0.6511228438	0.6049002543	-2.989400452
C	-0.0470242901	0.0609586977	-4.1113739148
C	-1.0349208121	2.6289835921	4.2795242363
H	-1.6667384888	3.5320923083	4.2244351429
H	0.0099338359	2.9658198898	4.2751080254
H	-1.2747533891	2.1270691186	5.225998049
C	-1.3625508231	1.7858925469	3.0896386797
C	-0.7381511628	2.019463116	1.8353961136
H	0.0740076696	2.7412185225	1.7563386227
C	-2.4062350778	0.8037676563	3.1485493334
H	-2.8655952819	0.5655738148	4.1074808914
C	-1.2412136485	1.3898284625	0.661128935
H	-0.7846565254	1.6238965022	-0.2995665853
C	-2.8397796813	0.1347804475	1.9891125051
H	-3.6099742252	-0.630858351	2.076680558
C	-2.3228021223	0.4850926017	0.6954211124
C	-2.9800894853	0.0718084337	-0.6045014882
H	-2.1892806713	0.0380949693	-1.3678547309
C	-3.9493420955	1.2095171623	-0.9940494125
H	-4.4072700324	0.9847476102	-1.9691701141

H	-3.4287626889	2.1754218222	-1.0739897644
H	-4.7564819468	1.3129676842	-0.2518060326
C	-3.7046962713	-1.2728416617	-0.5900196207
H	-3.034408091	-2.0966969502	-0.3124541363
H	-4.10599528	-1.4793322389	-1.5933259801
H	-4.5538158665	-1.2723013397	0.110933759
I	-1.1768331907	-2.950556635	1.8809587338
S	0.3280367945	-0.9421765635	4.2520098431
C	-1.0628226668	-1.2597570604	5.3950588122
H	-1.5189901238	-0.3063223346	5.687178149
H	-1.7771152287	-1.9069259983	4.874003667
H	-0.6294033223	-1.7668575505	6.2673220012
C	1.2242297178	0.2695120866	5.2862748856
H	2.2194343707	0.4019074334	4.8528288709
H	0.6893679142	1.2201603053	5.3441728963
H	1.3034655665	-0.1965240715	6.2770348293
O	1.2178423254	-2.1829999813	4.2948645812

[RuCl(*p*-cym)], E = -1687.64047630 au, $\square_{1}$ = 16.4000 cm^{-1}

Ru	-1.379093751	-0.1633586756	-0.5236981118
P	-0.188006594	-0.6422025838	1.4404134134
C	-3.7280620073	-0.2979468215	-0.7324913553
C	-3.1277030949	0.1185212886	-1.9795074471
H	-3.4535406147	1.0589523317	-2.4274739143
C	-2.1294517696	-0.6343067287	-2.6208921251
H	-1.7123383522	-0.2879807334	-3.5670212178
C	-1.5851116295	-1.8041495169	-1.9893735291
C	-2.1659897248	-2.2010617971	-0.746094255
H	-1.7985948913	-3.1041378139	-0.2632482193
C	-3.2561804342	-1.4850859124	-0.1509026244

H	-3.6746470821	-1.8386485793	0.789711573
C	-4.8442312198	0.5282449991	-0.1272834097
H	-4.6565616676	1.5725217538	-0.4293028952
C	-6.1899277049	0.0940175148	-0.7369297775
H	-7.0023282972	0.7369220602	-0.3649025608
H	-6.4246208966	-0.9471948119	-0.4642532117
H	-6.1765841122	0.1630945048	-1.8353283969
C	-4.8819144772	0.4802777939	1.4023825885
H	-5.6401513007	1.1818033097	1.7810257734
H	-3.9094866092	0.7643155072	1.8331692911
H	-5.145810925	-0.5212170462	1.7763810168
C	-0.5154110364	-2.6230323389	-2.6457217105
H	0.0801820813	-3.1649103801	-1.8979091629
H	0.1562644275	-1.9927799054	-3.2446902265
H	-0.9720547002	-3.3658520603	-3.3204965997
C	0.539266218	0.2531279029	-1.1872555524
C	0.7673392574	0.8275951368	-2.4593873416
H	-0.0841678762	1.1244620504	-3.0746107942
C	2.0459968738	1.0506465281	-2.973932536
H	2.1594108667	1.4932193141	-3.9682232605
C	3.0246362404	0.1753482143	-0.9290092815
C	4.1643498159	-0.1539602035	-0.1352408707
C	6.593522366	-0.2890647973	0.1439087576
H	7.600457657	-0.1381849322	-0.2534677587
C	6.4157893579	-0.8071691975	1.4295565868
H	7.2894551814	-1.0610088494	2.0343158897
C	5.1350505135	-1.0010961499	1.9509835784
H	5.0040882346	-1.401651518	2.9593561062
C	3.9937215171	-0.681061321	1.1873059645
C	2.6620921096	-0.8537420356	1.6911666034
H	2.5394329031	-1.2320146287	2.7096298998

C	1.5732755982	-0.5460161783	0.915960794
C	1.7055458604	-0.0425430742	-0.4184345506
C	-0.3334644222	0.6277727123	2.86864973
C	-0.2775588584	-2.3239134826	2.302461066
H	0.3265396571	-2.1470397903	3.2069038343
C	3.2009386806	0.7199755988	-2.23924976
C	5.4847173849	0.048493358	-0.6578393085
C	5.6272465131	0.5911841635	-1.9802467833
C	4.5311861276	0.915817649	-2.7352274071
H	4.6560110275	1.3336409919	-3.7380118351
H	6.6360294786	0.7434774309	-2.3724629882
C	0.3963129158	-3.4551102954	1.5205905662
H	0.3116029969	-4.38948262	2.0989647652
H	-0.0699223263	-3.6299824328	0.541581182
H	1.4639029408	-3.2624251898	1.3511787303
C	-1.6997828302	-2.6992636138	2.7369771144
H	-2.2589674184	-3.1619212515	1.9132458484
H	-1.651111898	-3.4402875723	3.5504642037
H	-2.2835463598	-1.8418422765	3.1009204278
H	-1.341326369	1.0428371539	2.708744841
C	-0.2500503115	0.0557629753	4.2865219329
H	-0.3825752205	0.8834850678	5.0027059406
H	-1.0253472019	-0.6906368262	4.5035682279
H	0.7345750378	-0.3931318242	4.4887765658
C	0.6937569737	1.7543017919	2.7037279895
H	0.4051605139	2.6035847896	3.3439827835
H	1.6942782074	1.4193743303	3.0152832952
H	0.7639598258	2.1241792034	1.6729287678
I	-1.6194756363	2.6952864092	-0.0564856398

PR_3, E = -1192.61374049 au, □$_1$= 26.5662 cm^{-1}

P 0.564690691 2.5345342528 0.6391409194

C -0.2650580697 1.0775171626 -1.7073777941

H -0.4123159741 2.0404084267 -2.1916684311

C -0.4012113813 -1.3485528909 -1.8757759349

C -0.9791897176 2.6423468161 1.7518386469

C 0.8548312585 -0.4262141391 1.5657292267

H 1.1321707503 0.463817743 2.1349553402

C -0.1021037362 -3.9186957387 -0.6760929173

C 0.0540714737 -5.1780077605 -0.0648931844

H -0.1871496629 -6.0772168951 -0.6372118273

C -0.9892363794 3.9886751076 2.4843477295

H -0.0233304438 4.1983074785 2.970239335

H -1.7633574471 3.9830537488 3.268726501

H -1.2155402975 4.8243775193 1.805160344

C -2.3262354446 2.3155200538 1.1042669134

H -2.607283758 3.0448096832 0.3321851768

H -3.1176292513 2.3237962506 1.8729808548

H -2.3243450196 1.3178883842 0.6417639804

C 0.5103536811 -5.2829276898 1.2511035043

H 0.6256906636 -6.2688756144 1.7067400665

C 0.1764103299 1.0226509334 -0.3744976926

C 1.0034054876 -1.6602718681 2.1365424849

H 1.3837140419 -1.75129961 3.1572506515

C 0.679055196 -2.8578419287 1.4195606248

C -0.5643281643 -3.7803857302 -2.0280821298

H -0.8008738981 -4.6863932016 -2.5917788456

C 0.3675830583 -0.2595362396 0.2233208371

C -0.7059932388 -2.5474911942 -2.6022287399

H -1.0572695522 -2.4524176016 -3.632926191

C 0.8209613784 -4.1389872216 1.9883232937

H 1.1798877715 -4.22558283 3.0168561127

C 0.21275177 -2.7365590684 0.0717683344

C -0.5423018124 -0.0714139766 -2.4450586824

H -0.8900538375 0.0165358603 -3.4776285182

C 0.0586606561 -1.4465952343 -0.523400885

C -0.7851700338 4.5275556384 -1.1571262918

H -1.1979087247 3.812575398 -1.881437805

H -0.5880900359 5.4652027221 -1.7046159904

H -1.5652626265 4.7427116158 -0.4156139566

C 1.6743665002 3.9650023664 -1.5061069957

H 2.6161912077 3.6660096911 -1.0202171372

H 1.835778127 4.9481671843 -1.9770862584

H 1.4700951628 3.2439816724 -2.311389582

C 0.5166302767 4.0523236529 -0.5000574461

H -0.7706744841 1.858469244 2.5006573486

H 0.7987384192 4.8242325971 0.2389939605

DMSO, E = -552.910920382 au, $\square_1$= 192.6239 cm^{-1}

C 0.0724732948 0.7715706133 -1.3682389328

H 0.1744845135 0.2087554737 -2.3051101725

H 0.7902179799 1.6040197219 -1.3557881211

H -0.9570375047 1.1357011325 -1.2477134738

C 0.0721881175 0.7715763143 1.3682403287

H -0.9568976619 1.1366450451 1.2469393484

H 0.1728860383 0.208370062 2.3050196921

H 0.7906930445 1.6033796374 1.3566613647

S 0.4689930068 -0.3890331953 0.0000293182

O -0.6337165787 -1.4775541251 -0.000109022
